# Differential Gene Expression between Leaf and Rhizome in *Atractylodes lancea*: A Comparative Transcriptome Analysis

**DOI:** 10.3389/fpls.2016.00348

**Published:** 2016-03-30

**Authors:** Qianqian Huang, Xiao Huang, Juan Deng, Hegang Liu, Yanwen Liu, Kun Yu, Bisheng Huang

**Affiliations:** College of Pharmacy, Hubei University of Chinese MedicineWuhan, China

**Keywords:** differentially expressed gene, Illumina sequencing, rhizome formation, rhizomatous plants, tissue-specific genes, transcription factor

## Abstract

The rhizome of *Atractylodes lancea* is extensively used in the practice of Traditional Chinese Medicine because of its broad pharmacological activities. This study was designed to characterize the transcriptome profiling of the rhizome and leaf of *Atractylodes lancea* in an attempt to uncover the molecular mechanisms regulating rhizome formation and growth. Over 270 million clean reads were assembled into 92,366 unigenes, 58% of which are homologous with sequences in public protein databases (NR, Swiss-Prot, GO, and KEGG). Analysis of expression levels showed that genes involved in photosynthesis, stress response, and translation were the most abundant transcripts in the leaf, while transcripts involved in stress response, transcription regulation, translation, and metabolism were dominant in the rhizome. Tissue-specific gene analysis identified distinct gene families active in the leaf and rhizome. Differential gene expression analysis revealed a clear difference in gene expression pattern, identifying 1518 up-regulated genes and 3464 down-regulated genes in the rhizome compared with the leaf, including a series of genes related to signal transduction, primary and secondary metabolism. Transcription factor (TF) analysis identified 42 TF families, with 67 and 60 TFs up-regulated in the rhizome and leaf, respectively. A total of 104 unigenes were identified as candidates for regulating rhizome formation and development. These data offer an overview of the gene expression pattern of the rhizome and leaf and provide essential information for future studies on the molecular mechanisms of controlling rhizome formation and growth. The extensive transcriptome data generated in this study will be a valuable resource for further functional genomics studies of *A. lancea*.

## Introduction

Rhizomatous plants comprise a large group, and many of them contribute ecosystem services (e.g., prevention of soil erosion) or have high economic value (e.g., ginger) or significant medicinal uses, such as *Paris polyphylla* and other rhizomatous medicinal plants (Glover et al., 2010; Yu et al., 2013). Leaf and rhizome are the two most important vegetative organs in rhizomatous plants. It is well known that the main role of leaves is to capture light energy, perform photosynthesis, and accumulate assimilates, while rhizomes primarily store energy reserves (e.g., starch) and allocate nutrients for overwintering and regrowth. The botanical, physiological, and genetic processes in the leaf and rhizome have always drawn intense attention (Bell and Tomlinson, 1980; Yu et al., 2013; Kong et al., 2015). However, the relationship of growth and development between leaf and rhizome and the molecular mechanisms underlying rhizome formation are largely not understood, due to the complexity of the developmental connections and the physiological coordination between the two organs.

Recently developed functional genomics approaches offer an efficient way to dissect complex physiological processes (Baginsky et al., 2010). The large scale of genomic and transcriptomic data have greatly enhanced our understanding of plant growth and development, especially in model plants, such as Arabidopsis and rice. The ability to sequence cDNA libraries has been exploited in functional genomics research in recent years (Fang et al., 2015). The advent of next-generation sequencing technologies has revolutionized functional genomics due to its high-throughput, sensitivity, and accuracy. In particular, RNA sequencing (RNA-Seq), has been widely used to obtain transcriptome data, profile global gene expression, and identify novel genes in both model and non-model plant species, including Arabidopsis (Begara-Morales et al., 2014), rice (Wakasa et al., 2014), *Salvia miltiorrhiza* (Gao et al., 2014), and *Medicago truncatula* (Cabeza et al., 2014). With advances in sequencing technology, RNA sequencing has become an effective and powerful tool for transcriptome analysis, especially in non-model species where limited genetic and genomic resources are available (Dillies et al., 2013).

*Atractylodes lancea* (Thunb.) DC. (Compositae), also called Cangzhu in Chinese, is a well-known and widely prescribed traditional Chinese herb. The rhizome of *A. lancea* has been used for the treatment of digestive disorders, rheumatic diseases, night blindness, and other conditions which is explained by eliminating dampness, invigorating spleen, and expelling wind according to the theory of traditional Chinese medicine (Committee, 2010). Modern pharmacological studies show that *A. lancea* has broad pharmacological effects on the nervous, gastrointestinal, and cardiovascular systems (Koonrungsesomboon et al., 2014). Anticancer, antimicrobial, and anti-inflammatory activities have also been demonstrated for the crude extracts of the *A. lancea* rhizome and its major constituents, such as atractylodin, β-eudesmol, hinesol, and atractylone (Resch et al., 2001; Wang et al., 2009; Zhao et al., 2014). *A. lancea* is widely distributed in East Asia, especially in China (Shi, 1987). However, natural populations of *A. lancea* have been rapidly depleted due to intense predatory exploitation. Artificial cultivation is thus imperative to protect the natural resources and achieve a sustainable supply. A crucial question is how to ensure or even improve rhizome quality. Though the phytochemistry, pharmacology, botany, and cultivation of *A. lancea* have been extensively studied, the molecular mechanisms of the plant's growth and development are poorly understood, largely due to the lack of genomic information (Deng et al., 2014; Koonrungsesomboon et al., 2014).

In this study, we performed high-throughput Illumina sequencing to comprehensively characterize the transcriptome of *A. lancea*, and reveal differential gene expression profiles between rhizome and leaf, which would facilitate uncovering the molecular mechanisms of regulating rhizome formation and growth of the most important medicinal plants in genus *Atractylodes*.

## Materials and methods

### Plant materials

Leaves and rhizomes of *A. lancea* were collected from Mao Mountain, Jiangsu Province, China (31°39′N, 119°19′E). All samples were harvested, washed, surface dried, and then immediately frozen in liquid nitrogen and stored at −80°C until RNA extraction. Two biological replicates were used for RNA extraction and transcriptome sequencing of the leaf and three replicates for the rhizome.

### RNA sequencing and *de novo* assembly

Total RNA from each tissue was isolated using the RNAprep Pure Plant Kit (Tiangen, Beijing, China) following the manufacturer's instructions. cDNA library construction and normalization were performed according to published protocols (Zhang et al., 2015). Five cDNA libraries (2 for leaves and 3 for rhizomes) were finally sequenced using an Illumina HiSeq2000 platform, and paired-end reads were generated. Clean reads were obtained by removing the adapter sequences, low quality sequences, and sequences shorter than 35 bases. The remaining high-quality reads were de novo assembled into candidate unigenes using the Trinity program (Grabherr et al., 2011).

### Functional annotation of unigenes

Assembled unigenes were annotated using BLAST alignment against public databases, including the NCBI non-redundant protein database (NR, http://www.ncbi.nlm.nih.gov), Swiss-Prot (http://www.expasy.ch/sprot), TrEMBL (http://www.ebi.ac.uk/trembl), Gene Ontology (GO, http://www.geneontology.org), and the Kyoto Encyclopedia of Genes and Genomes (KEGG, http://www.genome.jp/kegg) with an E value cutoff of 10^−5^. Blast2GO and WEGO program was carried out to perform GO annotation and to obtain GO classifications according to molecular function, biological process, and cellular component (Conesa et al., 2005; Ye et al., 2006). The transcription factor (TF) families were identified by Using known plant transcription factors identified in PlnTFDB (http://plntfdb.bio.uni-potsdam.de/v3.0) based on the annotation.

### Sequence analysis

Analysis of codon usage bias was performed by CodonW (http://codonw.sourceforge.net/). Putative SSR markers were detected using the MISA software package (http://pgrc.ipk-gatersleben.de/misa/). The minimum repeat number was 10 for mono-nucleotides, 6 for di-nucleotide, and 5 for tri-, tetra-, penta-, and hexa-nucleotide.

### Determination of unigene expression level

Since no reference genome was available for *A. lancea*, the clean reads from each sequencing library were mapped back to the assembled unigenes using Bowtie with a maximum mismatch of 2 nucleotides (Langmead et al., 2009). The expression level of each unigene were normalized and calculated as the value of fragments per transcript kilobase per million fragments mapped (FPKM), which eliminates the influence of different gene lengths and sequencing discrepancies (Trapnell et al., 2010).

### Analysis of unigene differential expression

The differential gene expression analysis of two assigned libraries was performed using the edgeR package (Robinson et al., 2010). The differentially expressed genes (DEGs) were screened with the threshold false discovery rate (FDR) < 0.05 and the absolute value of log2FoldChange > 1. Subsequently, GO functional enrichment analysis and KEGG pathway analysis of the DEGs were performed using GOseq and KOBAS, respectively (Young et al., 2010; Xie et al., 2011).

### Quantitative real-time PCR

Twenty unigenes (c40786_g1, c53153_g2, c45414_g1, c40381_g1, c33812_g1, c37348_g1, c29120_g1, c36168_g1, c41101_g1, c44073_g2, c41696_g3, c45627_g1, c37834_g1, c39104_g1,c47165_g4, c49171_g1,c43275_g1, c38241_g1, c51805_g3, and c45003_g1) were selected for verification of the sequencing and computational results by quantitative real-time PCR (qPCR). All reactions were carried out in 96-well plates in the StepOne Real-Time PCR System (Applied Biosystems, Foster City, CA, USA) using the SYBR Premix Ex Taq II (TaKaRa, Dalian, China) kit with four replicates. Cycling conditions were 95°C for 10 min followed by 45 cycles of 94°C for 30 s and 60°C for 45 s. The relative expression levels of the selected unigenes were normalized to the internal control gene Tubulin (c50304_g2), and determined by the ΔΔCt-method. All primers used are shown in Supplementary Table 1.

## Results

### Sequence analysis and assembly

**Figure 1:**
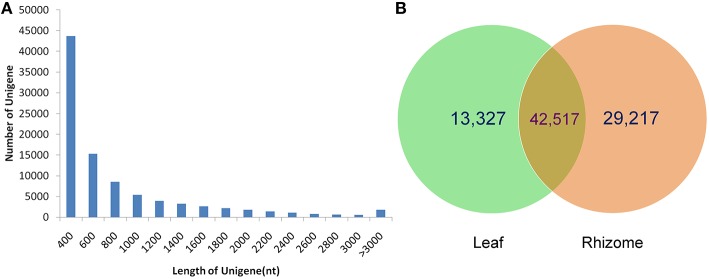
**Length distribution of unigenes from samples of leaf and rhizome (A)**. The Venn diagram shows the number of expressed genes (FPKM >1) in samples of leaf and rhizome **(B)**.

To obtain a comprehensive overview of the *A. lancea* transcriptome, RNAseq libraries were constructed from leaves and rhizomes and sequenced using Illumina paired-end sequencing technology. After the removal of adaptor sequences and low-quality reads, approximately 118.4 and 152.7 million clean reads were acquired for the leaf and rhizome transcriptomes, respectively. Thus, a total of 33,885 Mb valid data were acquired with an average length of 125 bp. An overview of the sequencing statistics is shown in Table 1. All clean reads were subsequently subjected to de novo assembly with the Trinity program resulting in 185,544 transcripts. A total of 92,366 unigenes with an average length of 721 bp, a maximum size of 15.9 kb, and an N50 of 1.1 kb (i.e., 50% of the assembled bases were incorporated into unigenes of 1.1 kb or longer) were obtained (Table 2). The GC content of the reads and unigenes distributed within 41–45% (Tables 1, 2). The size distribution of the *A. lancea* unigenes is given in Figure 1A, with 27% of all unigenes showing lengths longer than 1 kb. A Venn diagram of the expressed unigenes with FPKM ≥1 is shown in Figure 1B. A total of 42,517 unigenes were found to be both expressed in leaf and rhizome samples of *A. lancea*. All reads generated in this study have been deposited in the National Center for Biotechnology Information (NCBI) and can be accessed in the Short Read Archive (SRA) Sequence Database under accession number SRP068251.

**Table 1:** **Summary of transcriptomes from leaf and rhizome in *A. lancea***.

**Item**	**Sample**	**Number (n)**	**Total nucleotides (bp)**	**GC percentage (%)**	**Q20 percentage (%)**
Raw read	Leaf	135,809,414	16,976,176,750	44.83	89.81
	Rhizome	176,759,032	22,094,879,000	44.50	89.61
Clean read	Leaf	118,397,448	14,799,681,000	44.66	91.73
	Rhizome	152,688,850	19,086,106,250	44.31	91.57

**Table 2:** **Assembly results of clean reads**.

	**Number (n)**	**Total nucleotides (bp)**	**GC percentage (%)**	**Average length**	**N50 (bp)**	**Max length (bp)**	**Min length (bp)**
Transcript	185,544	149,559,536	41.05	806	1,183	15,933	400
Unigene	92,366	66,627,502	40.94	721	1,141	15,933	400

### Functional annotation and classification

The unigenes were aligned against public protein databases (NR, Swiss-Prot, GO, and KEGG) using BLAST with a cut-off E-value of 1.0e^−5^. A total of 39,664 unigenes (42.90% of the total assembled unigenes) had a match in the NR database, and 38,699 (41.19%), 26,159 (28.32%), and 10,508 (11.38%) unigenes showed significant similarity to sequences in the Swiss-Prot, GO, and KEGG databases, respectively (Table 3).

**Table 3:** **Statistics of annotations for assembled unigenes**.

**Category**	**Account**	**Percentage^c^ (%)**
Nr^a^	39,664	42.90
Blast-hit^b^	38,699	41.90
eggnog classified unigenes	12,602	13.64
GO classified unigenes	26,159	28.32
KEGG classified unigenes	10,508	11.38
All annotated unigenes	53,894	58.35

a *NCBI non-redundant database*.

b *SWISSPROT and TREMBLE database*.

c *Percentage of annotated unigenes in total 92,366 assembled unigenes*.

**Figure 2:**
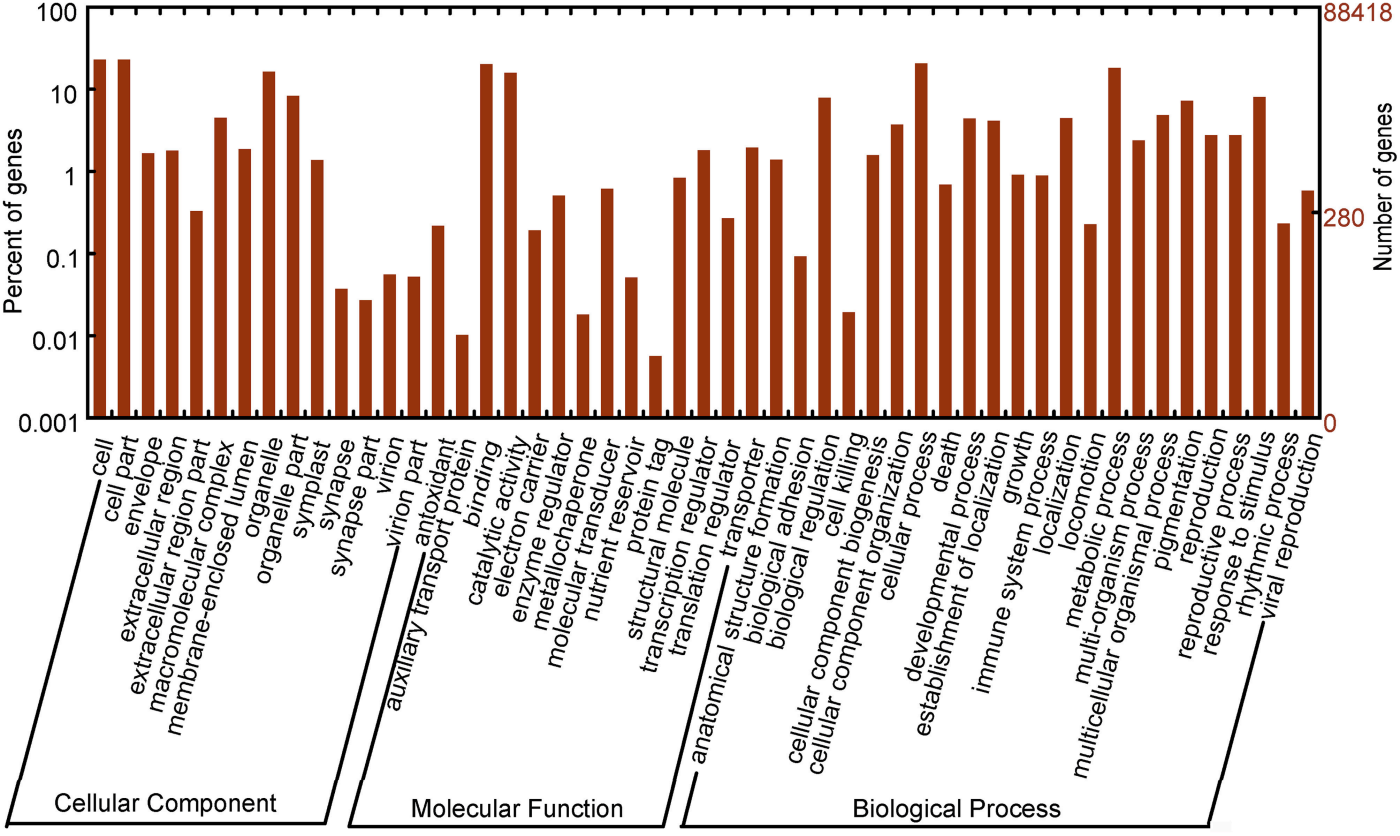
**Gene ontology classification of assembled unigenes**.

GO classification was used to classify unigene functions based on the Nr annotation, and 26,159 (28.32%) unigenes were assigned to one or more GO terms (Figure 2). Within the “biological process” domain, the assignments were mostly enriched in the terms “cellular process” (21,094, 22.84%), “metabolic process” (16,157, 17.49%), “response to stimulus” (7133, 7.72%), and “biological regulation” (6762, 7.32%). In the “molecular function” domain, the terms “catalytic activity” (14,221, 15.40%) and “binding” (17,944, 19.43%) were mostly assigned. For the “cellular component” domain, the most evident matches were to the terms “cell” (19,140, 20.72%), “cell part” (19,098, 20.68%), “organelle” (14,672, 15.88%), and “organelle part” (7317, 7.92%).

**Figure 3:**
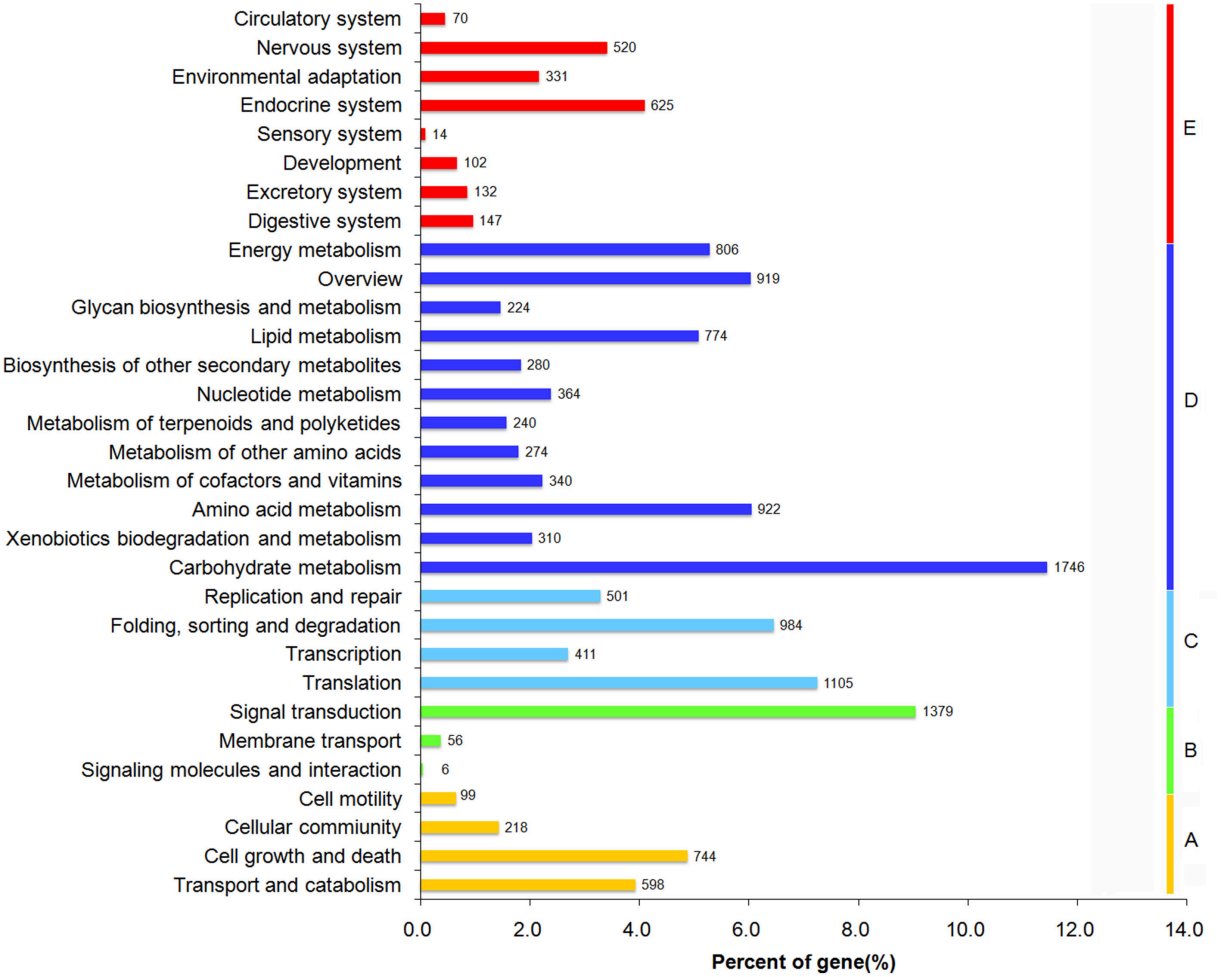
**Functional classification and pathway assignment of assembled unigenes by KEGG**.

KEGG pathway analysis was performed to identify the biochemical pathways active in the leaf and rhizome of *A. lancea*. A total of 10,504 unigenes were annotated and assigned to 289 KEGG pathways. Unigenes classified to the five main KEGG biochemical pathways, metabolism, genetic information processing, environmental information processing, cellular processes, and organismal systems pathways are presented in Figure 3, with unigenes associated with Human Diseases filtered out. The three most highly represented pathways are “carbon metabolism” (ko01200), “Ribosome” (ko03010), and “Biosynthesis of amino acids” (ko01230).

Cytochromes P450 (CYP450s) form by far the largest superfamily of plant enzymes and take part in numerous primary and secondary metabolic processes (Weitzel and Simonsen, 2015). In the *A. lancea* transcriptome data, 161 unigenes were functionally annotated as CYP450s; the genes belong to 71 CYP family categories, with the majority in the CYP72A219 family (Supplementary Table 2).

### Characterization of codon usage and SSR markers

Codon usage analysis was based on 1556 full-length sequences with ORF ≥600 bp. The codon usage Table was created from 9.2 million codons (Supplementary Table 3). GAT was the most frequently used codon with the occurrence frequency 3.81% followed by GAA (3.54%) and AAG (3.05%).

A total of 10,103 SSRs were identified in 92,366 unigenes, 1074 of which contained more than one SSR, and 406 SSRs were present in compound form (Table 4). The most abundant repeat motifs were mono-nucleotides (4440, 43.94%), followed by di-nucleotides (3604, 35.67%) and tri-nucleotides (1892, 19.62%).

**Table 4:** **Summary of SSR searching results**.

**Item**	**Number**
Total number of sequences examined	92,366
Total size of examined sequences (bp)	66,627,502
Total number of identified SSRs	10,103
Number of SSR containing sequences	8873
Number of sequences containing more than one SSR	1074
Number of SSRs present in compound formation	406
Mono-nucleotides	4440
Di-nucleotides	3604
Tri-nucleotides	1892
Tetra-nucleotides	114
Penta-nucleotides	16
Hexa- nucleotides	37

### Highly expressed and tissue-specific genes

We identified 227 transcripts in the leaf and 105 in the rhizome with an FPKM value greater than 1000, of these, 49 were in both tissues. Most of the highly expressed genes in the leaf were predominantly involved in photosynthesis, stress response, and translation; in the rhizome, transcripts involved in stress response were dominant, followed by those related to transcription regulation, translation, and metabolism (Supplementary Table 4). The 10 most abundant transcripts in leaf and rhizome are listed in Table 5.

**Table 5:** **Top 10 abundant transcripts in leaf and rhizome of *A. lancea***.

	**GeneID**	**FPKM value**	**Annotation**
Leaf	c32981_g1	24990.4	Ribulose bisphosphate carboxylase small chain
	c32959_g1	22189.3	Photosystem II 10 kDa polypeptide
	c52112_g4	13016.9	Metallothionein-like protein
	c42290_g2	11063.1	Chlorophyll a-b binding protein
	c49898_g1	10120.0	Catalase isozyme
	c49821_g5	9472.1	Photosystem II reaction center W protein
	c35432_g1	9291.8	Uncharacterized protein
	c40816_g1	8862.8	Calvin cycle protein CP12-1
	c45468_g1	7859.4	Glutamine synthetase nodule isozyme
	c30221_g1	7685.7	Photosystem I reaction center subunit XI,
Rhizome	c47723_g1	75468.6	Root allergen protein
	c46420_g1	40983.1	Acidic endochitinase SE2
	c47723_g3	27786.8	Root allergen protein
	c36255_g1	15480.5	Defensin SD2
	c47665_g3	13686.4	Translationally-controlled tumor protein homolog
	c35232_g1	6453.7	Metallothionein-like protein
	c34062_g1	5635.3	Uncharacterized protein
	c47113_g5	5517.8	Peroxidase 42
	c51076_g1	4891.0	Manganese transport protein mntH
	c52915_g5	4691.7	Heat shock cognate 70 kDa protein

Genes that are represented by more than 10 reads in one tissue and no more than one read in another are considered tissue specific (Zhang et al., 2014). According to these criteria, we identified 697 leaf-specific genes from several gene families, including UDP-glycosyl transferases, CYP450s, and ethylene responsive transcription factors (Supplementary Table 5). Chlorophyll binding proteins and photosystem proteins were shown to be highly expressed in the leaf, as expected. Several transcription factors were also specifically expressed in leaf, such as bHLH87-like, WRKY, MYB, and TCP5-like TFs. Of the 469 rhizome-specific genes we identified, we recorded high FPKM values for zinc finger genes, non-specific lipid transfer genes, and many secondary metabolism-related genes (e.g., isocomene synthase, vinorine synthase-like protein, N-benzoyltransferase, etc.). Several gene families were also enriched in the rhizome-specific set, such as leucine-rich repeat receptor-like kinases, heat shock proteins, and transcription factors (Supplementary Table 6).

### DEGs between rhizomes and leaves

**Figure 4:**
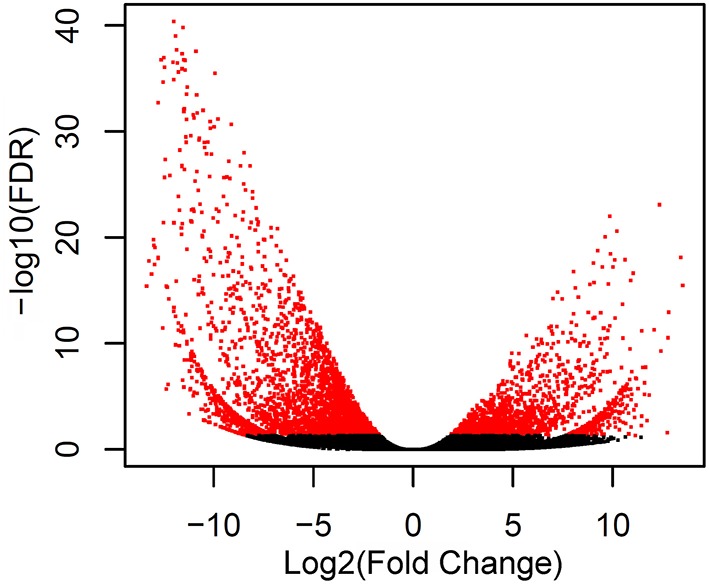
**Volcano plot of the transcriptome in leaf and rhizome**. The horizontal line and vertical lines indicate the significance threshold (FDR < 0.05) and two-fold change threshold (|log2FoldChange|>1), respectively. The DEGs are shown with blue dots while non-DEGs are in black.

To identify different expression levels of genes between rhizomes and leaves of *A. lancea*, we calculated the FPKM values of assembled unigenes. We found 4982 differentially expressed unigenes between the two tissues, including 1518 genes and 3464 genes down-regulated in the rhizome compared with the leaf. A volcano plot was constructed to illustrate the distribution of significantly regulated genes (Figure 4).

In order to further understand the biological functions of the DEGs, enrichment analyses based on GO and KEGG pathways were performed. When the 4982 DEGs were checked against the GO database, 262 GO terms were significantly enriched (Supplementary Figure S1). In the KEGG analysis, the 1518 up-regulated unigenes were linked to 159 KEGG pathways. The pathway assigned the largest number of unigenes (29) was “plant hormone signal transduction” (ko04075), followed by “starch and sucrose metabolism” (ko00500), “protein processing in endoplasmic reticulum” (ko04141), and “biosynthesis of amino acids” (ko01230; Table 6). 39 unigenes were mapped to secondary metabolism pathways, including 18 unigenes which might be involved in “metabolism of terpenoids and polyketides” (Supplementary Table 7).

**Table 6:** **List of KEGG Pathway with more than 10 DEGs annotated**.

**Pathway**	**Pathway ID**	**DEGs with pathway annotation**	**All genes with pathway annotation**	**Percentage (%)**
Plant hormone signal transduction	ko04075	29	269	10.787
Starch and sucrose metabolism	ko00500	18	256	7.03
Biosynthesis of amino acids	ko01230	15	373	4.02
Protein processing in endoplasmic reticulum	ko04141	15	352	4.26
Galactose metabolism	ko00052	12	94	12.77
Carbon metabolism	ko01200	12	433	2.77
Amino sugar and nucleotide sugar metabolism	ko00520	11	173	6.36
Terpenoid backbone biosynthesis	ko00900	10	75	13.33
Cell cycle	ko04110	10	199	5.03
Phagosome	ko04145	10	123	8.13
Gap junction	ko04540	10	43	23.26

**Figure 5:**
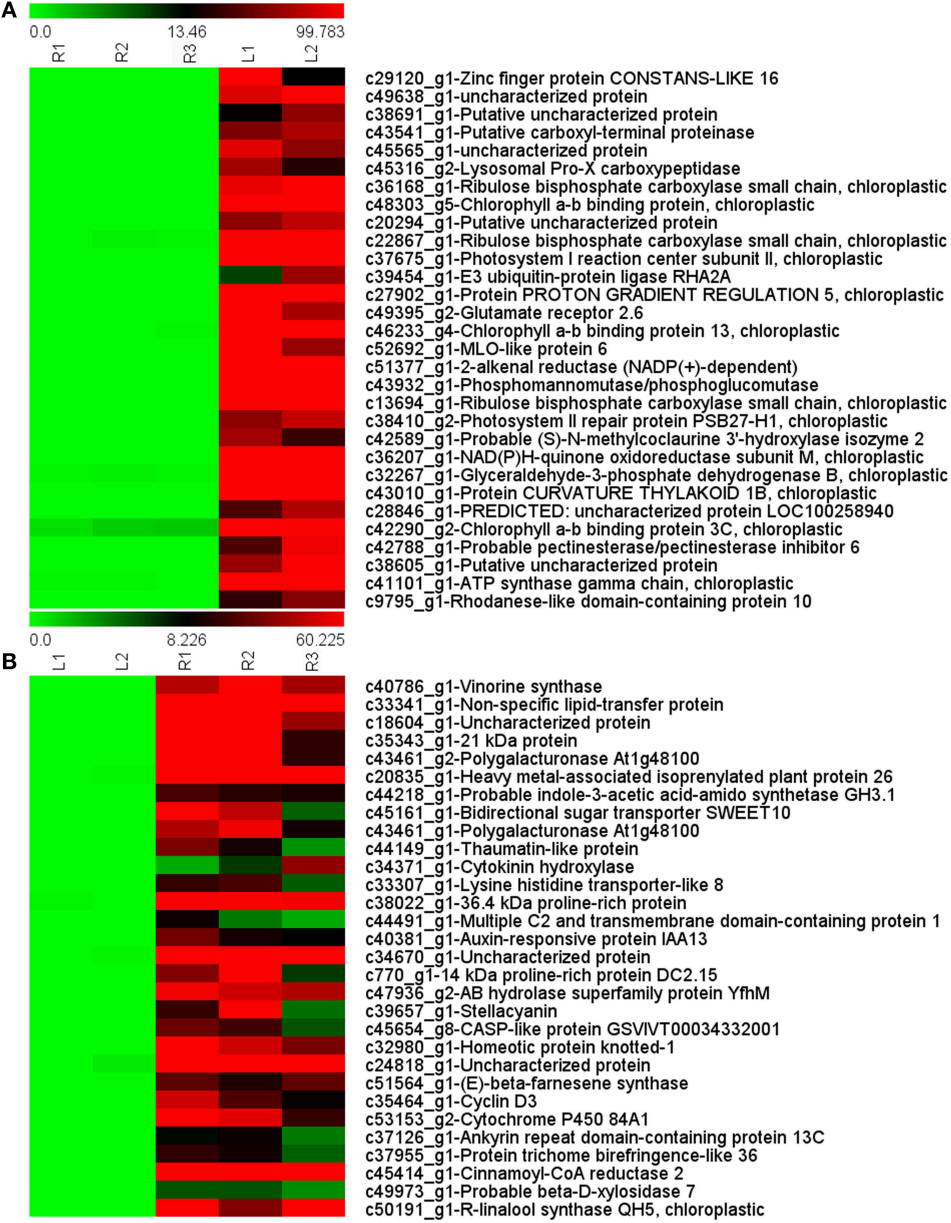
**List of top 30 up-regulated transcripts in leaf (A) and rhizome (B)**. L, leaf; R, rhizome.

The top 30 up-regulated unigenes in the leaf and rhizome are shown in Figure 5. Unigenes involved in photosynthesis (e.g., ribulose bisphosphate carboxylase, chlorophyll a-b binding protein) the main up-regulated genes in the leaf, while unigenes associated with plant hormones (e.g., cytokinin hydroxylase and auxin-responsive proteins) and secondary metabolism (such as farnesene synthase and cinnamoyl-CoA reductase) were up-regulated in the rhizome.

### Validation of DEGs by qPCR

The RNA-Seq and computational results were verified by qPCR using 20 selected DEGs. The expression patterns of all the selected genes show the same trend in the transcriptome analysis and the qRT-PCR (Supplementary Figure S2). We also tested the correlations of these genes and found a significant positive correlation between them, with the correlation coefficient reaching 0.81.

### Identification of TF families

**Figure 6:**
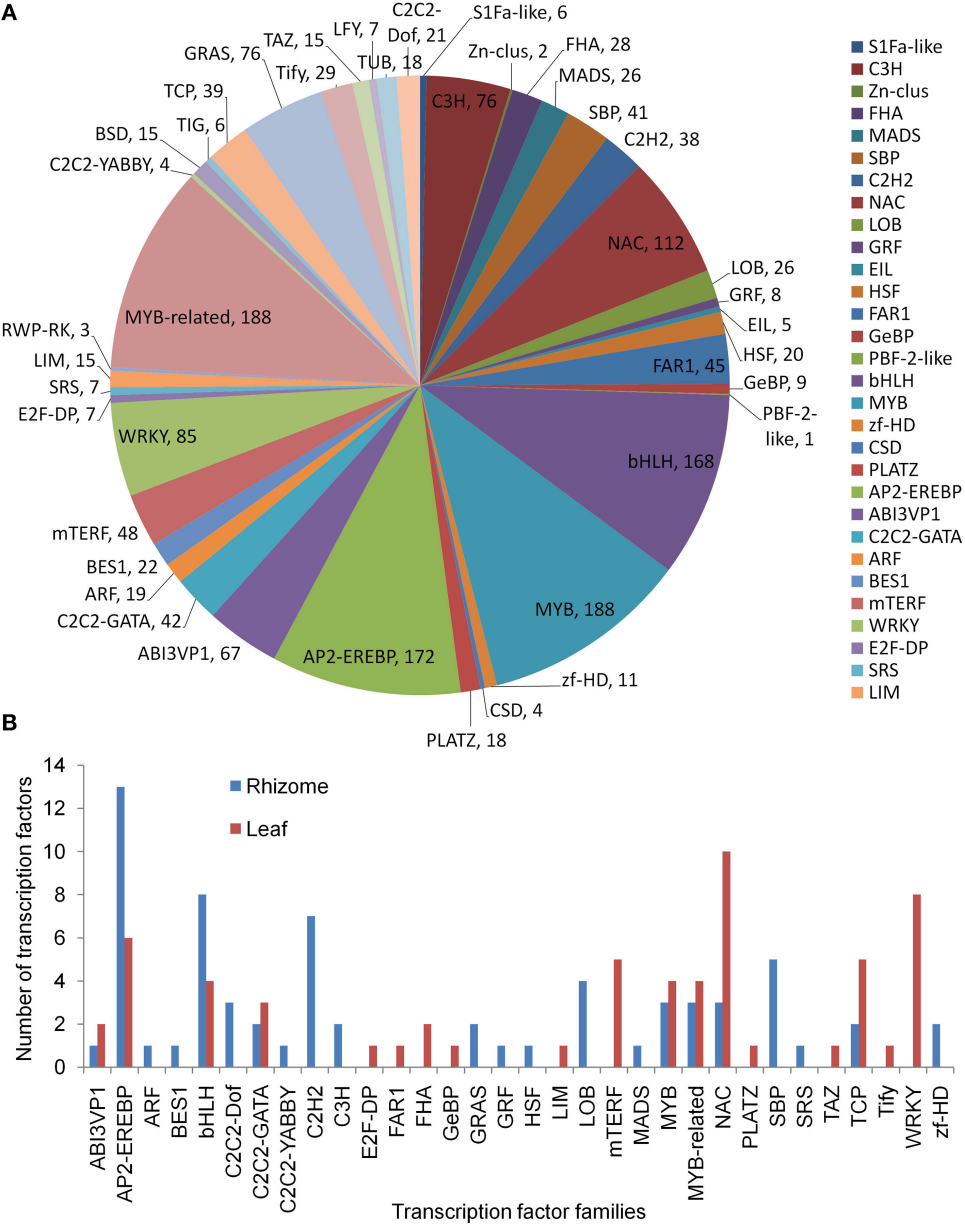
**Transcription factor analysis. (A)** Distribution of transcription factor families. **(B)** Up-regulated transcription factors in leaf and rhizome.

A total of 42 TF families were identified when aligning the annotated *A. lancea* transcripts to the AGRIS database (Figure 6A). Members of the MYB, MYB-related, AP2-EREBP, bHLH, NAC, WRKY, C3H, GRAS, ABI3VP1, and mTERF families were the top 10 classes, each with more than 48 unigenes. There were 60 TFs up-regulated in the leaf, mainly from the NAC, WRKY, AP2-EREBP, mTERF, and TCP families. A total of 67 TFs were up-regulated in the rhizome, mainly from the AP2-EREBP, bHLH, C2H2, and SBP families (Figure 6B).

### Genes involved in rhizome formation and growth

**Figure 7:**
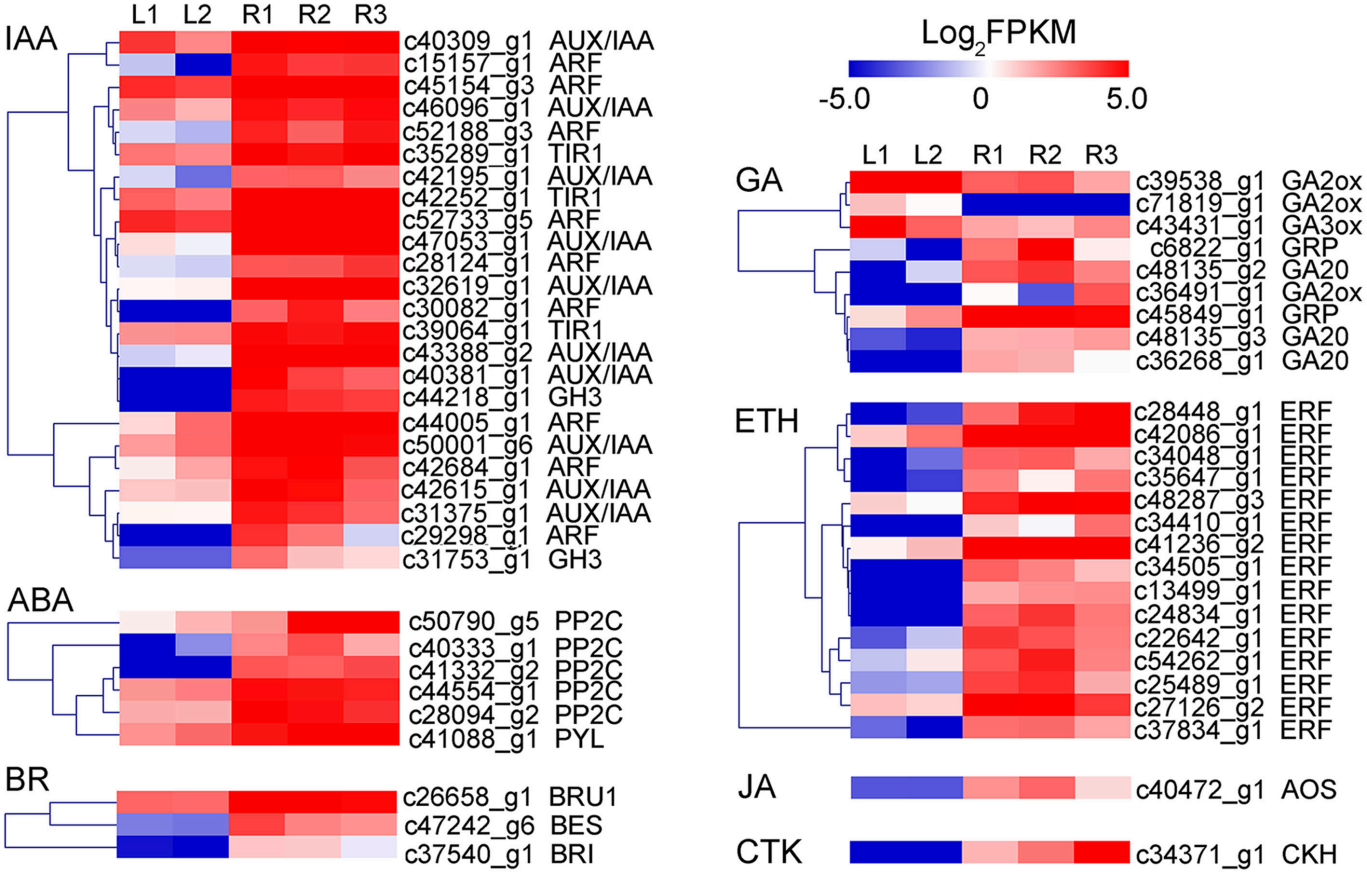
**Heatmap of the expressed genes assigned to hormone responsive protein, hormone biosynthesis pathway, and signal transduction pathway in leaf and rhizome transcriptomes**. AOS, allene oxide synthase; ARF, auxin response factor; BES, brassinazole-resistant protein; BRI, BR Insensitive 1; BRU1, Brassinosteroid-regulated protein; CKH, cytokinin hydroxylase; ERF, Ethylene-responsive transcription factor; GA20, Gibberellin 20-oxidase; GA2ox, Gibberellin 2-beta-dioxygenase; GA3ox, Gibberellin 3-beta-dioxygenase; GH3, IAA-amido synthetase; GRP, Gibberellin-regulated protein; PP2C, 2C type protein phosphatase; PYL, abscisic acid receptor; TIR1, transport inhibitor response 1.

DEGs were further analyzed to screen candidate genes involved in rhizome formation and development. A total of 104 genes involved in organ development, hormone biosynthesis, and hormone signal transduction were identified, including some transcription factors (e.g., 2 MADS-box proteins, 12 AP2-like transcription factors). Unigenes encoding sucrose synthase, lipoxygenase, and gibberellin (GA) 20-oxidase were also identified as candidates (Table 7), as well as a number of genes involving in hormone response, biosynthesis, and signal transduction. Candidate genes in the auxin (IAA), GA, abscisic acid (ABA), ethylene (ETH), cytokinin (CTK), jasmonic acid (JA), and brassinosteroid (BR) pathways are shown in Figure 7.

**Table 7:** **Putative unigenes involved in the rhizome formation and growth**.

**Unigene ID**	**Annotation**
c36342_g2, c39228_g1, c22969_g1, c34893_g1, c1015_g1	LOB (LOB domain containing protein)
c51159_g2, c44467_g2	MADS-box (MADS-domain proteins)
c46989_g2, c46695_g1	Patatin
c41236_g2, c28448_g1, c35388_g1, c48287_g3, c13499_g1, c34505_g1, c25489_g1, c27126_g2, c27126_g1, c42086_g1, c35647_g1, c34410_g1	AP2-like transcription factor
c37914_g2, c8176_g1, c32129_g2, c37914_g3, c23583_g1	DOF (DNA binding with One Finger proteins)
c48784_g1, c63135_g1	BEL1-like homeodomain protein
c41333_g1	Sucrose synthase
c48982_g1, c52646_g2, c52646_g4, c43238_g1, c41952_g1, c49181_g1, c37075_g1, c49113_g1, c24141_g1	Calmodulin-like protein
c54015_g1	Lipoxygenase
c48135_g2, c48135_g3, c36268_g1	GA 20-oxidase
c44800_g1, c35499_g1, c38237_g1	Zinc finger CONSTANS-like protein

## Discussion

### Global gene transcription in the leaf and rhizome of *A. lancea*

Deep RNA sequencing is currently an effective choice for studying the transcriptome of non-model plant species, including *A. lancea*. Transcript profiling and comparative transcriptome analysis have frequently been used to identify differentially expressed genetic networks and the expression patterns of genes in different organs or tissues of plants. As genome resources for the genus *Atractylodes* are not yet available, Illumina-based RNA sequencing was used to profile the transcriptome of *A. lancea*, one of the most important medicinal plants in the genus *Atractylodes*. We obtained 271 million clean sequencing reads which were assembled de novo into 92,366 unigenes. Of these, 53,894 unigenes (about 58.4% of the assembled unigenes) could be functionally annotated against public protein databases (NR, Swiss-Prot, GO, and KEGG), while no functional annotation was found for 41.6% of the assembled unigenes, either due to a match with a protein of unknown function or because no homologous nucleotide sequence matched (Table 2). These unigenes may be of great importance for further research, since they may be considered novel transcripts or alternative splice variants.

Analysis of gene expression levels was employed to profile global gene expression in the leaf and rhizome, the two main vegetative tissues of *A. lancea*, analysis of gene expression levels was employed. Genes related to photosynthesis and stress response, such as ribulose bisphosphate carboxylase, photosystem II polypeptide, and metallothionein-like protein, had the highest expression levels in the leaf. This observation is in agreement with previous reports (Mizrachi et al., 2010; Brown et al., 2012; Zhang et al., 2014). Several gene families were identified to be leaf-specific, including UDP-glycosyl transferases, cytochrome P450 proteins, and ethylene responsive transcription factors. UDP-glycosyl transferases play important roles in the biosynthesis of natural plant products, such as terpenoids and flavonoids, and in the regulation of plant hormones (Yonekura-Sakakibara and Hanada, 2011). Plant cytochrome P450s participate in a wide range of biochemical pathways that produce primary and secondary metabolites, such as lipids, terpenoids, and plant hormones (Mizutani and Ohta, 2010). Ethylene responsive transcription factors, members of the AP2/ERF superfamily, are implicated in diverse biological events, such as hormonal signal transduction, response to biotic and abiotic stress, and metabolism regulation (Nakano et al., 2006; Mizoi et al., 2012). These leaf-specific genes may all be functionally related to leaf growth, development, and metabolic processes.

In the rhizome, highly expressed genes were predominantly those involved in stress response, such as defensin, allergen, antioxidative enzymes, acidic endochitinase, and metallothionein-like protein. Similar results were found in the rhizomes of *Ligusticum chuanxiong* and *Sorghum propinquum* (Zhang et al., 2014; Song et al., 2015). The tissue-specific genes were largely distinct between leaf and rhizome. The plant zinc finger proteins, which belong to a large family of transcription factors, play a variety of important roles in growth and development, hormone response, and response to abiotic and biotic stresses (Li et al., 2013). Non-specific lipid transfer proteins are known to play key roles in plant defense, growth and development (Liu et al., 2015). A variety of enzymes involved in secondary metabolism, such as the biosynthesis of terpenoids, alkaloids, and isoflavone, were also identified as rhizome-specific, which is similar to the results in *S. propinquum* (Zhang et al., 2014).

### DEGs in leaf and rhizome transcriptome of *A. lancea*

Differential gene expression patterns were investigated to further profile global gene expression differences between leaf and rhizome. Most of the DEGs identified in the rhizome were assigned to hormone signal transduction, primary metabolic pathways (carbohydrate metabolism and protein biosynthesis), or some secondary metabolic pathways, such as terpenoid biosynthesis and phenylpropanoid biosynthesis, which is in accord with the rhizome's physiological function as a storage organ for carbohydrates and essential oils. The list of the top 30 up-regulated rhizome transcripts is consistent with this (Figure 5). In addition, other genes involved in primary metabolism (non-specific lipid transfer protein, lysine histidine transporter, etc.) and secondary metabolism (vinorine synthase, farnesene synthase, cinnamoyl-CoA reductase, etc.) were also up-regulated in the rhizome. Notably, some stress response-related genes were remarkably up-regulated. Heavy metal-associated isoprenylated plant proteins have been demonstrated to be involved in heavy metal homeostasis and detoxification, response to cold and drought, and plant–pathogen interactions (de Abreu-Neto et al., 2013). Polygalacturonase and bidirectional sugar transporters were both found to be responsible for plant-microbe interactions and important physiological processes in plants, including cell separation, and phloem transport (Yu et al., 2014; Chen et al., 2015).

Gene expression patterns in the leaf were quite different. Genes associated with photosynthesis (ribulose bisphosphate carboxylase, chlorophyll binding protein, etc.) and fundamental metabolism (carboxyl-terminal proteinase, lysosomal pro-x carboxypeptidase, etc.) were greatly up-regulated, in addition to genes associated with plant developmental events and stress tolerance. The zinc finger protein CONSTANS is known to play a role in flowering time and stress tolerance (Yang et al., 2014). MLO proteins have been found to be associated with various developmental pathways and biotic and abiotic stresses (Deshmukh et al., 2014). Alkenal reductase has been proposed to have a key role in the detoxification of reactive carbonyls (Mano et al., 2005). The expression pattern of these genes suggests that they play specific roles in physiological processes in the rhizome and leaf.

TFs play a paramount role in governing plant growth and development by specifically binding to the cis-acting elements in the promoters of downstream genes (Yang et al., 2012). RNA-seq emerged as a powerful tool for the identification of various TF families not only in model plants but also in medicinal plants, such as *Salvia miltiorrhiza* (Gao et al., 2014) and *Bupleurum chinense* (Sui et al., 2015). Of the 42 TF families identified in total in this study, TFs belonging to 18 and 22 families were found to be up-regulated in the leaf and rhizome, respectively. The MYB and bHLH TFs are members of two of the largest plant TF families, and function in diverse biological processes, such as the regulation of primary and secondary metabolism, hormone signal transduction, defense, and stress response (Feller et al., 2011). The NAC, WRKY, AP2-EREBP, and C2H2 zinc-finger TFs have all been shown to function in plant developmental processes and stress responses (Chen et al., 2012; Mizoi et al., 2012; Nakashima et al., 2012; Razin et al., 2012), while the mTERF transcription factors are mainly targeted to mitochondria or chloroplasts (Kleine and Leister, 2015). Among the TF families identified in our data, the MYB, bHLH, WRKY, NAC, and AP2-EREBP TFs have been reported to be involved in regulating secondary metabolic pathways (Yang et al., 2012). However, further studies are needed to ascertain and unravel their underlying mechanism of action in *A. lancea*.

### Complex regulation of rhizome formation and development in *A. lancea*

As storage organ derived from modified stems, the rhizome serves as a deposit for photosynthates and other compounds, such as essential oils in *A. lancea*. However, the mechanisms governing rhizome formation and growth are poorly understood. The development of other storage organs, such as potato tubers and lotus rhizomes, has been extensively studied (Fernie and Willmitzer, 2001; Yang et al., 2015). Rhizome formation and development are complex developmental processes in rhizomatous plants; they have been reported to be regulated by a combination of environmental stimuli (e.g., photoperiod) and endogenous factors (Cheng et al., 2013; Yang et al., 2015).

Short day conditions promote the formation of storage organs through the regulation of proteins related to photoperiod signal transduction, such as CONSTANS, cycling Dof factors, and the AP2-like TFs (Martinez-Garcia et al., 2002; Imaizumi and Kay, 2006; Cheng et al., 2013). Patatin, MADS-box transcription factors, BEL1-like homeodomain proteins, and calmodulin-like proteins are also believed to be involved in storage organ development (Hannapel et al., 1985; Bamfalvi et al., 1994; Banerjee et al., 2006; Kim et al., 2009). Plant lateral organ development could be regulated by LOB domain-containing proteins (Majer and Hochholdinger, 2011). Sucrose synthase, a starch biosynthesis enzyme, and lipoxygenase also play important roles in the growth of storage organs (Fernie and Willmitzer, 2001; Kolomiets et al., 2001). Candidate genes encoding such proteins were identified in this study and may participate in rhizome formation and growth of *A. lancea*.

Plant hormones have been reported to play important roles in the formation of storage organs (Fernie and Willmitzer, 2001). GA and ABA act antagonistically during plant development, including the process of storage organ formation (Yu et al., 2009). GA prevents the formation of potato tubers and lotus rhizomes, while ABA promotes these processes (Xu et al., 1998; Yang et al., 2015). The effects of JA on the induction of storage organs have been extensively studied in potato, yam, garlic, and lotus (Koda, 1992; Zel et al., 1997; Cheng et al., 2013). Moreover, ethylene, auxin, cytokinins, and brassinosteroids also have positive effects on tuber initiation and development (Vreugdenhil and Struik, 1989; Peres et al., 2005). In our study, a range of GA, ABA, ethylene, cytokinin, JA, and brassinosteroid related genes were detected, indicating the complexity of the gene regulatory networks and developmental processes involved in rhizome formation in *A. lancea*. Further studies on these candidate genes might be useful to uncover the mechanisms of rhizome formation and development in *A. lancea*.

## Conclusions

In this study, 92,366 unigenes, including 38,472 novel genes, were assembled, and 4982 DEGs were identified. Highly expressed and tissue-specific genes were also identified, as well as TF families in leaves and rhizomes. The comparative transcriptome analysis revealed clear differences in global gene expression profile between the leaf and rhizome, suggesting specific and complex molecular mechanisms regulating the growth and development of these two organs. In addition, 104 DEGs were identified to be relevant to rhizome formation and development. These results reveal the coordination of the vegetative organs of rhizomatous plants at the transcriptional level. The sequence datasets and analysis reported here will facilitate functional genomics, gene discovery, transcriptional regulation, and applied studies in *A. lancea* and other *Atractylodes* species. DEGs and potential candidate genes involving in rhizome formation and development will help for illustrating the molecular mechanisms underlying rhizome formation and growth.

## Author contributions

QH, XH, and JD prepared the material for sequencing and analyzed the data. HL participated in data analysis. YL is the main coordinator of the project and participated in the conception of the study together with KY and BH. KY and BH were responsible for drafting and revising the manuscript.

### Conflict of interest statement

The authors declare that the research was conducted in the absence of any commercial or financial relationships that could be construed as a potential conflict of interest.
