# Supplementary material for: Differential Gene Expression between Leaf and Rhizome in Atractylodes lancea: A Comparative Transcriptome Analysis
Source: Front Plant Sci. 2016 Mar 30;7:348. doi: 10.3389/fpls.2016.00348 (PMC4811964; doi:10.3389/fpls.2016.00348)
Supplement: Supplementary file 1 [file Table1.docx]

**Supplementary Table 1** List of primer sequences used for qPCR.

| Unigene NO. | Forward Primer | Reverse Primer | Target Size (bp) |
| --- | --- | --- | --- |
| c40786_g1 | AAATGATGGCTATGTTTCTACG | ATCGACCTCAATGAGTCTGCTA | 239 |
| c53153_g2 | CGTAACAAGAATGGTTTGGG | CTCAATGCCCTGTAGATC | 134 |
| c45414_g1 | TGTTGCTTCGTGGGTAGTG | TTCGTGTTCCTGTTATGGC | 179 |
| c40381_g1 | CAAGAATGAAGAGGAGAAGGG | AGCCGAGAGTGTTGCTTTG | 280 |
| c33812_g1 | CGAACTTCCTCTTCCTTGC | AACTACCCACTCATAACCGC | 175 |
| c37348_g1 | CGAATCGTAAGGGTATCTAAGG | TGAGGAACTGAGGCTGGTA | 271 |
| c29120_g1 | TGGTATTGTGCTGCCGAT | GACGAGATGAATGCTTCCC | 210 |
| c36168_g1 | TGGTGGAGAACGGGAAGG | CTTTTGCTCAACATTTGGG | 75 |
| c41101_g1 | GAGCAAATACTCTCTATGG | GTCCATGCAAACTCTTGAG | 225 |
| c44073_g2 | CGCGTATAAATCCATTGC | CGAAGGTGATAAGGAAGCC | 209 |
| c50304_g2 | GTGGACGAAAGCCCTCTT | CGCACCATTCAGTTTGTTG | 204 |
