# Supplementary material for: Differential Gene Expression between Leaf and Rhizome in Atractylodes lancea: A Comparative Transcriptome Analysis
Source: Front Plant Sci. 2016 Mar 30;7:348. doi: 10.3389/fpls.2016.00348 (PMC4811964; doi:10.3389/fpls.2016.00348)
Supplement: Supplementary file 4 [file Table4.docx]

**Supplementary Table 4** List of genes with more than 1000 FPKM in leaf and rhizome.

| NO. | GeneID | FPKM value | | | | | Annotation |
| --- | --- | --- | --- | --- | --- | --- | --- |
|  |  | Leaf 1 | Leaf 2 | Rhizome 1 | Rhizome 2 | Rhizome 3 |  |
| 1 | c42403_g1 | 1661.6 | 802.2 | 4071.3 | 2146.4 | 1570.8 | Superoxide dismutase [Cu-Zn] |
| 2 | c35348_g1 | 4925.6 | 351.1 | 2.0 | 2.1 | 1.0 | Ribulose bisphosphate carboxylase small chain 2, chloroplastic |
| 3 | c30418_g1 | 1098.3 | 162.2 | 126.5 | 59.3 | 91.6 | Uncharacterized protein |
| 4 | c49821_g5 | 9472.1 | 4019.3 | 3.2 | 1.9 | 4.3 | Photosystem II reaction center W protein, chloroplastic |
| 5 | c51399_g2 | 1690.3 | 865.7 | 8.6 | 7.7 | 1.6 | Oligopeptide transporter 4 |
| 6 | c41445_g1 | 1030.2 | 518.8 | 1599.7 | 647.4 | 927.3 | Protein translation factor SUI1 homolog |
| 7 | c43275_g1 | 572.9 | 403.1 | 1800.7 | 2467.8 | 1781.4 | S-adenosylmethionine synthase 2 |
| 8 | c47016_g1 | 1028.8 | 169.9 | 156.8 | 139.5 | 78.1 | Uncharacterized membrane protein C2G11Uncharacterized protein09 |
| 9 | c36292_g1 | 35.3 | 9.5 | 2796.2 | 2955.9 | 2377.5 | Beta-fructofuranosidase, soluble isoenzyme I |
| 10 | c32959_g1 | 22189.3 | 10317.2 | 24.4 | 10.0 | 6.7 | Photosystem II 10 kDa polypeptide, chloroplastic |
| 11 | c43521_g2 | 1863.4 | 671.5 | 0.6 | 0.5 | 0.3 | Thioredoxin F-type, chloroplastic |
| 12 | c46233_g4 | 1555.0 | 893.6 | 0.2 | 0.2 | 0.3 | Chlorophyll a-b binding protein 13, chloroplastic |
| 13 | c53463_g1 | 987.5 | 1000.6 | 1608.4 | 1548.2 | 897.4 | Uncharacterized protein |
| 14 | c52926_g1 | 1756.8 | 652.2 | 260.2 | 207.8 | 43.3 | Tetraspanin-8 |
| 15 | c51649_g2 | 1335.9 | 818.8 | 948.7 | 564.9 | 509.8 | Histone H3Uncharacterized protein3a |
| 16 | c43181_g1 | 2810.8 | 581.4 | 297.1 | 39.6 | 66.5 | Glutathione S-transferase U17 |
| 17 | c50992_g1 | 397.4 | 173.3 | 2320.0 | 1264.4 | 1064.6 | YTH domain-containing family protein 2 |
| 18 | c49004_g1 | 446.7 | 1622.7 | 4309.0 | 1472.3 | 1861.4 | Root allergen protein |
| 19 | c37134_g1 | 78.8 | 60.2 | 3864.7 | 4270.5 | 519.8 | Protein TsetseEP |
| 20 | c52185_g1 | 3533.8 | 4207.7 | 644.4 | 311.3 | 847.9 | Omega-6 fatty acid desaturase, endoplasmic reticulum |
| 21 | c48722_g1 | 313.9 | 384.9 | 1208.2 | 1372.3 | 572.0 | GDP-L-galactose phosphorylase 1 |
| 22 | c32267_g1 | 2771.8 | 1217.6 | 0.6 | 0.4 | 0.4 | Glyceraldehyde-3-phosphate dehydrogenase B, chloroplastic |
| 23 | c43378_g3 | 1354.5 | 57.1 | 175.0 | 25.8 | 31.9 | Uncharacterized protein |
| 24 | c44748_g1 | 4990.5 | 1209.7 | 1293.4 | 612.4 | 1605.4 | Late embryogenesis abundant protein Lea5 |
| 25 | c51776_g1 | 1462.6 | 445.8 | 279.5 | 107.2 | 369.2 | Omega-3 fatty acid desaturase, chloroplastic |
| 26 | c42161_g1 | 4930.7 | 1333.4 | 656.5 | 176.5 | 337.6 | Protein TIFY 10A |
| 27 | c52994_g1 | 1027.4 | 101.5 | 342.0 | 282.3 | 382.8 | 3-oxo-Delta(4,5)-steroid 5-beta-reductase |
| 28 | c40021_g1 | 3252.7 | 434.4 | 963.0 | 185.5 | 250.4 | Polyubiquitin |
| 29 | c40642_g1 | 1442.7 | 418.0 | 321.0 | 90.2 | 273.2 | Uncharacterized protein |
| 30 | c47095_g1 | 298.2 | 298.2 | 1185.1 | 337.3 | 664.5 | Histone H1 |
| 31 | c28400_g1 | 1153.5 | 367.0 | 358.7 | 366.3 | 451.2 | Zinc finger A20 and AN1 domain-containing stress-associated protein 11 |
| 32 | c45487_g1 | 817.7 | 545.3 | 2135.9 | 1394.5 | 1189.2 | 40S ribosomal protein S15a |
| 33 | c36501_g1 | 1117.6 | 287.9 | 769.7 | 398.7 | 214.9 | Putative uncharacterized protein |
| 34 | c51852_g1 | 2026.5 | 2058.5 | 456.5 | 462.5 | 383.3 | Aspartic proteinase A3 |
| 35 | c44869_g1 | 207.9 | 97.7 | 1135.1 | 226.0 | 754.2 | Heat shock protein 83 |
| 36 | c44207_g1 | 1230.4 | 631.4 | 21.2 | 29.6 | 77.8 | Nudix hydrolase 8 |
| 37 | c53441_g1 | 740.2 | 1134.9 | 1313.0 | 750.8 | 250.2 | Cytochrome P450 716B2 |
| 38 | c45468_g1 | 5027.6 | 7859.4 | 692.3 | 457.7 | 346.3 | Glutamine synthetase nodule isozyme |
| 39 | c46642_g1 | 438.0 | 420.7 | 1069.8 | 717.5 | 1016.0 | S-adenosylmethionine synthase 3 |
| 40 | c40348_g1 | 2468.0 | 1509.8 | 70.2 | 38.6 | 72.8 | Catalase isozyme 1 |
| 41 | c36732_g1 | 1005.6 | 782.8 | 428.9 | 442.9 | 281.7 | Actin-depolymerizing factor 3 |
| 42 | c35477_g1 | 1137.6 | 907.3 | 668.6 | 705.1 | 820.6 | Glyceraldehyde-3-phosphate dehydrogenase, cytosolic |
| 43 | c51165_g4 | 1061.7 | 628.1 | 6.6 | 5.1 | 3.1 | Photosystem I reaction center subunit IV, chloroplastic |
| 44 | c50452_g1 | 1043.6 | 643.5 | 14.3 | 11.8 | 19.4 | 1-deoxy-D-xylulose 5-phosphate reductoisomerase, chloroplastic |
| 45 | c48924_g1 | 705.9 | 811.3 | 1724.3 | 1225.3 | 1184.6 | Elongation factor 1-alpha |
| 46 | c35232_g1 | 2534.2 | 2626.7 | 6453.7 | 6528.3 | 4001.8 | Metallothionein-like protein 1 |
| 47 | c51279_g2 | 1273.0 | 916.6 | 45.1 | 41.2 | 40.8 | Glutamate--glyoxylate aminotransferase 2 |
| 48 | c48174_g1 | 1527.4 | 1672.1 | 7.3 | 4.4 | 6.0 | Uncharacterized protein |
| 49 | c48163_g3 | 1139.5 | 145.7 | 297.5 | 133.3 | 133.5 | Uncharacterized protein |
| 50 | c37521_g1 | 1336.1 | 961.5 | 57.7 | 44.2 | 33.3 | Thioredoxin M-type, chloroplastic |
| 51 | c47111_g2 | 1095.9 | 456.7 | 199.2 | 135.0 | 168.9 | Uncharacterized protein |
| 52 | c48891_g1 | 2458.7 | 1111.9 | 14.1 | 14.6 | 12.2 | Chlorophyll a-b binding protein 8, chloroplastic |
| 53 | c47040_g1 | 3485.0 | 2639.8 | 1.8 | 1.9 | 1.1 | Serine--glyoxylate aminotransferase |
| 54 | c43318_g1 | 1160.1 | 456.8 | 623.0 | 492.7 | 595.9 | Polyubiquitin |
| 55 | c51047_g1 | 1.6 | 1.0 | 248.2 | 1336.0 | 2.8 | Dehydration-responsive protein RD22 |
| 56 | c47665_g3 | 5614.7 | 1738.2 | 13686.4 | 9966.1 | 4348.0 | Translationally-controlled tumor protein homolog |
| 57 | c45360_g1 | 1870.8 | 1134.7 | 4.4 | 3.7 | 3.3 | Oxygen-evolving enhancer protein 1, chloroplastic |
| 58 | c48891_g2 | 2596.4 | 1450.9 | 0.7 | 1.1 | 0.3 | Chlorophyll a-b binding protein 8, chloroplastic |
| 59 | c40816_g1 | 8862.8 | 3395.3 | 11.6 | 5.4 | 6.8 | Calvin cycle protein CP12-1, chloroplastic |
| 60 | c15284_g1 | 1681.0 | 616.4 | 228.8 | 154.4 | 161.5 | Uncharacterized protein |
| 61 | c13694_g1 | 1525.8 | 1097.4 | 0.0 | 0.0 | 0.0 | Ribulose bisphosphate carboxylase small chain, chloroplastic |
| 62 | c37205_g1 | 2095.6 | 0.2 | 0.2 | 0.2 | 0.2 | Putative uncharacterized protein |
| 63 | c44690_g1 | 1152.0 | 256.6 | 1221.5 | 530.8 | 660.3 | Elongation factor 1-beta 2 |
| 64 | c39499_g1 | 123.2 | 72.1 | 1609.3 | 1023.6 | 518.1 | Aquaporin TIP2-1 |
| 65 | c43575_g9 | 1048.5 | 448.9 | 11.3 | 9.3 | 44.5 | Endochitinase B |
| 66 | c43026_g1 | 1955.6 | 1400.5 | 797.8 | 253.5 | 587.7 | Protein translation factor SUI1 homolog 2 |
| 67 | c20947_g1 | 1257.2 | 559.8 | 270.6 | 207.5 | 193.8 | Glutaredoxin-C4, chloroplastic |
| 68 | c41365_g1 | 1554.4 | 604.9 | 3.5 | 3.5 | 1.8 | ATP synthase subunit b', chloroplastic |
| 69 | c50051_g1 | 1061.3 | 415.4 | 73.1 | 21.1 | 32.8 | Transketolase, chloroplastic |
| 70 | c39743_g1 | 464.1 | 1078.4 | 0.4 | 0.6 | 0.7 | Major allergen Pru ar 1 |
| 71 | c44107_g1 | 179.8 | 165.0 | 1420.4 | 1257.4 | 1128.7 | Glycine-rich RNA-binding protein |
| 72 | c42290_g2 | 11063.1 | 8066.1 | 2.6 | 1.5 | 2.8 | Chlorophyll a-b binding protein 3C, chloroplastic |
| 73 | c45916_g2 | 1832.8 | 1137.1 | 484.2 | 315.2 | 331.9 | Thiamine thiazole synthase 1, chloroplastic |
| 74 | c43809_g1 | 2476.5 | 468.6 | 1.9 | 0.6 | 0.5 | Photosystem I reaction center subunit III, chloroplastic |
| 75 | c20465_g1 | 1368.0 | 162.6 | 1454.5 | 60.6 | 530.4 | Uncharacterized protein |
| 76 | c53042_g1 | 2099.3 | 632.9 | 0.3 | 5.4 | 0.3 | Uncharacterized protein |
| 77 | c47358_g4 | 2679.3 | 969.8 | 1315.9 | 579.4 | 670.1 | Probable auxin efflux carrier component 1c |
| 78 | c48599_g3 | 5485.8 | 2810.8 | 1.3 | 1.2 | 1.8 | Chlorophyll a-b binding protein 6A, chloroplastic |
| 79 | c48163_g1 | 1296.8 | 129.6 | 255.5 | 179.3 | 122.8 | Uncharacterized protein |
| 80 | c52149_g1 | 1320.0 | 2546.9 | 2074.8 | 1035.1 | 1226.5 | Putative uncharacterized protein Sb05g016477 |
| 81 | c47723_g1 | 276.8 | 3191.0 | 75468.6 | 47785.4 | 55822.8 | Root allergen protein |
| 82 | c34240_g2 | 1517.0 | 959.6 | 0.4 | 0.3 | 0.5 | Uncharacterized protein |
| 83 | c18630_g1 | 1405.3 | 455.5 | 36.6 | 21.2 | 34.6 | Uncharacterized protein |
| 84 | c27902_g1 | 1299.2 | 875.4 | 0.2 | 0.0 | 0.2 | Protein PROTON GRADIENT REGULATION 5, chloroplastic |
| 85 | c42669_g2 | 1121.9 | 607.9 | 0.2 | 1.5 | 0.9 | ATP synthase delta chain, chloroplastic |
| 86 | c45729_g1 | 2383.8 | 1137.5 | 268.6 | 176.1 | 279.8 | Glycerophosphodiester phosphodiesterase GDE1 |
| 87 | c43228_g1 | 691.6 | 147.0 | 2016.3 | 595.9 | 1516.6 | Non-specific lipid-transfer protein |
| 88 | c40359_g1 | 1062.9 | 665.4 | 93.4 | 50.7 | 88.5 | 4-hydroxy-3-methylbut-2-enyl diphosphate reductase, chloroplastic |
| 89 | c24818_g1 | 0.6 | 0.0 | 681.7 | 1182.2 | 108.5 | Uncharacterized protein |
| 90 | c35565_g1 | 1077.7 | 0.4 | 1.1 | 0.0 | 1.2 | Putative uncharacterized protein |
| 91 | c34775_g2 | 4.1 | 4.6 | 1251.7 | 1095.5 | 415.0 | Uncharacterized protein |
| 92 | c38494_g1 | 1027.0 | 376.5 | 509.3 | 241.6 | 275.4 | Cytochrome b-c1 complex subunit 8 |
| 93 | c42792_g2 | 31.9 | 43.2 | 2332.7 | 282.9 | 577.1 | BAG family molecular chaperone regulator 6 |
| 94 | c28734_g1 | 540.3 | 263.6 | 1185.5 | 367.9 | 337.0 | Uncharacterized protein |
| 95 | c35432_g1 | 9291.8 | 1671.1 | 3.6 | 1.5 | 1.8 | Uncharacterized protein |
| 96 | c49152_g1 | 441.2 | 303.1 | 1129.2 | 466.4 | 509.2 | Basic leucine zipper and W2 domain-containing protein 1-A |
| 97 | c37948_g1 | 2259.1 | 793.6 | 497.1 | 253.2 | 248.8 | Glutathione S-transferase APIC |
| 98 | c36284_g2 | 2354.1 | 1171.3 | 1.5 | 1.0 | 1.0 | Oxygen-evolving enhancer protein 1, chloroplastic |
| 99 | c33198_g1 | 4280.5 | 1732.9 | 46.6 | 27.1 | 7.9 | Cytochrome b6-f complex iron-sulfur subunit, chloroplastic |
| 100 | c32810_g1 | 1956.6 | 732.5 | 3046.2 | 937.3 | 1153.5 | Keratin, type I cytoskeletal 10 |
| 101 | c46252_g1 | 1784.5 | 100.0 | 36.8 | 44.8 | 34.3 | Galactinol synthase 2 |
| 102 | c42644_g1 | 1684.0 | 948.7 | 16.0 | 4.0 | 0.7 | Thioredoxin-like protein CDSP32, chloroplastic |
| 103 | c39758_g1 | 2339.1 | 1023.9 | 1.0 | 0.1 | 0.4 | Photosystem II 22 kDa protein, chloroplastic |
| 104 | c20735_g1 | 0.0 | 3367.7 | 0.0 | 11.7 | 2.5 | Root allergen protein |
| 105 | c51148_g1 | 978.1 | 652.5 | 1835.5 | 1367.6 | 1023.0 | Actin-2 |
| 106 | c30221_g1 | 7685.7 | 3112.0 | 4.0 | 5.3 | 4.4 | Photosystem I reaction center subunit XI, chloroplastic |
| 107 | c36986_g1 | 2048.4 | 945.8 | 1.4 | 0.4 | 0.8 | Putative uncharacterized protein |
| 108 | c49085_g1 | 4236.1 | 1439.5 | 2160.7 | 1170.9 | 2062.3 | Polyubiquitin |
| 109 | c53989_g1 | 87.5 | 22.1 | 2255.4 | 997.4 | 1391.9 | Beta-fructofuranosidase, soluble isoenzyme I |
| 110 | c49718_g1 | 1217.1 | 189.8 | 15.7 | 17.4 | 15.7 | Ribonuclease 2 |
| 111 | c43280_g2 | 1177.8 | 595.3 | 819.1 | 448.9 | 180.9 | Protein LURP-one-related 15 |
| 112 | c52539_g1 | 680.7 | 182.0 | 1287.6 | 593.1 | 1887.0 | Heat shock cognate 70 kDa protein 1 |
| 113 | c34969_g1 | 1355.1 | 401.1 | 108.5 | 15.7 | 33.6 | Uncharacterized protein |
| 114 | c22492_g1 | 4.2 | 1.1 | 2396.5 | 1411.5 | 1782.9 | Homeobox-leucine zipper protein ATHB-7 |
| 115 | c49048_g2 | 2853.5 | 1916.0 | 0.5 | 0.5 | 0.8 | Photosystem I reaction center subunit N, chloroplastic |
| 116 | c31382_g1 | 1964.2 | 1112.0 | 0.4 | 0.6 | 0.7 | Photosystem II core complex proteins psbY, chloroplastic |
| 117 | c34569_g1 | 731.8 | 430.2 | 1745.5 | 1411.8 | 1107.8 | ABC transporter F family member 1 |
| 118 | c52177_g4 | 1164.4 | 696.5 | 295.7 | 156.3 | 291.3 | Uncharacterized protein |
| 119 | c46694_g1 | 1455.4 | 436.6 | 1601.3 | 1073.1 | 696.1 | Uncharacterized protein |
| 120 | c50973_g1 | 808.8 | 1938.1 | 102.1 | 81.1 | 81.1 | Cysteine synthase, chloroplastic/chromoplastic |
| 121 | c28178_g1 | 1022.7 | 472.5 | 11.0 | 9.9 | 36.5 | NAC domain-containing protein 55 |
| 122 | c39489_g1 | 1324.9 | 403.1 | 3544.0 | 909.9 | 1739.2 | Zinc finger A20 and AN1 domain-containing stress-associated protein 8 |
| 123 | c33618_g1 | 2220.6 | 1536.7 | 250.6 | 268.4 | 299.6 | Uncharacterized protein |
| 124 | c51076_g1 | 90.6 | 5.6 | 4891.0 | 1442.7 | 2443.7 | Manganese transport protein mntH |
| 125 | c45781_g1 | 2755.4 | 2191.8 | 4232.5 | 1137.4 | 1705.0 | Uncharacterized protein |
| 126 | c45949_g3 | 1191.1 | 260.1 | 262.5 | 67.6 | 98.3 | Glyceraldehyde-3-phosphate dehydrogenase, cytosolic |
| 127 | c35361_g1 | 2674.7 | 1537.8 | 2.5 | 1.0 | 0.8 | Oxygen-evolving enhancer protein 1, chloroplastic |
| 128 | c36255_g1 | 145.8 | 96.2 | 15480.5 | 1445.2 | 11863.6 | Defensin SD2 |
| 129 | c3319_g1 | 1111.0 | 281.8 | 336.3 | 145.4 | 45.0 | Putative uncharacterized protein |
| 130 | c46873_g1 | 4226.5 | 518.0 | 219.5 | 41.4 | 217.4 | Inorganic pyrophosphatase 1 |
| 131 | c34401_g1 | 1035.5 | 839.3 | 148.9 | 99.0 | 91.8 | Cysteine synthase |
| 132 | c24804_g1 | 416.2 | 485.7 | 1085.6 | 407.5 | 876.1 | Uncharacterized protein |
| 133 | c45719_g2 | 1281.7 | 2613.6 | 2009.0 | 862.3 | 1151.2 | Uncharacterized protein |
| 134 | c38644_g1 | 1269.8 | 716.2 | 1811.5 | 1028.8 | 0.8 | Uncharacterized protein |
| 135 | c21807_g1 | 3243.5 | 3624.7 | 2563.2 | 766.9 | 1621.5 | Uncharacterized protein |
| 136 | c42985_g1 | 646.7 | 1060.5 | 285.5 | 166.6 | 162.6 | Metal ion binding protein, putative |
| 137 | c44585_g1 | 675.2 | 577.4 | 1323.4 | 497.0 | 746.8 | Vacuolar-processing enzyme |
| 138 | c35594_g1 | 1153.1 | 331.9 | 2793.5 | 1496.9 | 624.1 | Zinc finger protein 593 |
| 139 | c36504_g1 | 438.5 | 653.1 | 1108.8 | 866.7 | 164.0 | Putative uncharacterized protein |
| 140 | c44078_g3 | 3310.0 | 1955.6 | 78.2 | 68.4 | 94.8 | Uncharacterized protein |
| 141 | c33684_g1 | 1068.1 | 415.0 | 215.7 | 120.7 | 56.7 | Uncharacterized protein |
| 142 | c46526_g1 | 4017.3 | 3102.9 | 1230.3 | 695.2 | 972.4 | Thiol protease aleurain |
| 143 | c46587_g2 | 1068.0 | 202.4 | 57.0 | 47.1 | 23.1 | Putative uncharacterized protein |
| 144 | c1270_g1 | 7240.8 | 594.2 | 1.5 | 1.3 | 3.0 | Ribulose bisphosphate carboxylase small chain, chloroplastic |
| 145 | c39744_g1 | 243.1 | 353.6 | 2114.4 | 1308.0 | 1353.5 | Auxin-repressed 12Uncharacterized protein5 kDa protein |
| 146 | c46420_g1 | 807.8 | 112.3 | 40983.1 | 12719.3 | 4890.9 | Acidic endochitinase SE2 |
| 147 | c53726_g1 | 1694.0 | 1842.9 | 943.5 | 550.0 | 868.0 | Delta(12) fatty acid dehydrogenase |
| 148 | c40244_g1 | 2326.6 | 703.5 | 236.7 | 229.2 | 128.5 | Protein YLS9 |
| 149 | c54113_g3 | 1321.5 | 446.7 | 2652.8 | 757.7 | 1524.7 | Zinc finger A20 and AN1 domain-containing stress-associated protein 4 |
| 150 | c49692_g1 | 5254.1 | 2863.1 | 3.9 | 2.8 | 5.7 | Fructose-bisphosphate aldolase, chloroplastic |
| 151 | c15573_g1 | 1473.7 | 497.6 | 5.2 | 5.6 | 0.9 | Uncharacterized protein |
| 152 | c50713_g2 | 2663.4 | 233.4 | 336.0 | 101.9 | 255.1 | Postacrosomal sheath WW domain-binding protein |
| 153 | c32981_g1 | 24990.4 | 8587.7 | 6.9 | 4.9 | 6.9 | Ribulose bisphosphate carboxylase small chain, chloroplastic |
| 154 | c50883_g3 | 1219.7 | 1101.4 | 315.4 | 225.2 | 154.2 | Sorbitol dehydrogenase |
| 155 | c46249_g6 | 4233.8 | 2302.4 | 1.3 | 0.5 | 0.8 | Plastocyanin, chloroplastic |
| 156 | c35199_g1 | 3423.7 | 1442.3 | 0.5 | 0.7 | 0.8 | Oxygen-evolving enhancer protein 2, chloroplastic |
| 157 | c46565_g1 | 954.9 | 1389.0 | 18.8 | 30.0 | 7.9 | Glutathione S-transferase U19 |
| 158 | c28785_g1 | 457.0 | 96.2 | 1038.8 | 304.6 | 956.7 | Aquaporin TIP1-1 |
| 159 | c44812_g1 | 1325.7 | 389.0 | 757.1 | 415.8 | 312.8 | Uncharacterized protein |
| 160 | c38754_g1 | 3074.9 | 1020.2 | 1.8 | 1.7 | 1.1 | Ferredoxin-1, chloroplastic |
| 161 | c51328_g1 | 4592.5 | 324.7 | 1256.9 | 306.8 | 1544.5 | Water stress-inducible protein Rab21 |
| 162 | c50570_g1 | 1016.3 | 147.1 | 37.3 | 43.3 | 14.6 | Putative uncharacterized protein |
| 163 | c48230_g1 | 4072.8 | 2402.7 | 1902.1 | 1832.1 | 1729.3 | Uncharacterized protein |
| 164 | c49433_g5 | 1906.4 | 1642.3 | 504.2 | 292.4 | 376.6 | 1,2-dihydroxy-3-keto-5-methylthiopentene dioxygenase 3 |
| 165 | c47113_g5 | 14.7 | 12.8 | 3939.1 | 5517.8 | 1772.7 | Peroxidase 42 |
| 166 | c44905_g1 | 1287.6 | 195.0 | 30.7 | 4.0 | 7.1 | Protein NRT1/ PTR FAMILY 6Uncharacterized protein4 |
| 167 | c51122_g1 | 652.7 | 197.4 | 1133.8 | 638.7 | 636.1 | Midasin |
| 168 | c37710_g1 | 2304.8 | 656.5 | 590.3 | 248.3 | 275.9 | Polyubiquitin |
| 169 | c45524_g2 | 2.4 | 1.3 | 1393.2 | 1220.6 | 238.8 | Uncharacterized protein |
| 170 | c45694_g1 | 2041.5 | 191.6 | 1234.9 | 360.4 | 1026.7 | Allene oxide synthase |
| 171 | c49898_g1 | 10120.0 | 6171.5 | 1491.7 | 1267.5 | 1214.9 | Catalase isozyme 3 |
| 172 | c42290_g1 | 4173.9 | 3079.1 | 1.1 | 2.1 | 1.5 | Chlorophyll a-b binding protein 16, chloroplastic |
| 173 | c48554_g2 | 2341.0 | 1046.2 | 5.2 | 8.5 | 7.5 | Chlorophyll a-b binding protein P4, chloroplastic |
| 174 | c47885_g2 | 1108.1 | 307.0 | 95.3 | 45.7 | 20.7 | Probable non-specific lipid-transfer protein AKCS9 |
| 175 | c40607_g1 | 1101.6 | 146.8 | 0.7 | 1.0 | 1.2 | Early light-induced protein 1, chloroplastic |
| 176 | c44965_g1 | 1.7 | 2.4 | 1869.0 | 857.9 | 892.5 | Uncharacterized protein |
| 177 | c43038_g1 | 712.5 | 1112.8 | 1361.3 | 832.4 | 1087.4 | Omega-6 fatty acid desaturase, endoplasmic reticulum isozyme 2 |
| 178 | c35577_g1 | 3554.0 | 4325.6 | 136.6 | 123.4 | 123.4 | Uncharacterized protein |
| 179 | c38113_g2 | 762.3 | 1330.2 | 80.7 | 66.4 | 30.5 | Extensin-1 |
| 180 | c40180_g1 | 1915.1 | 569.7 | 622.4 | 256.6 | 277.8 | Nucleoside diphosphate kinase B |
| 181 | c43826_g1 | 43.8 | 42.3 | 1443.7 | 779.3 | 497.7 | Histidine decarboxylase |
| 182 | c15696_g1 | 23.4 | 72.8 | 1403.4 | 46.3 | 2092.1 | Uncharacterized protein |
| 183 | c53661_g1 | 753.6 | 1981.8 | 346.8 | 176.7 | 641.3 | Omega-6 fatty acid desaturase, endoplasmic reticulum isozyme 2 |
| 184 | c48816_g3 | 1264.8 | 965.8 | 109.6 | 64.7 | 46.2 | Malate dehydrogenase, glyoxysomal |
| 185 | c37749_g1 | 1957.8 | 441.5 | 51.2 | 77.1 | 22.0 | Low-temperature inducible |
| 186 | c45740_g1 | 274.4 | 155.7 | 1520.9 | 1289.4 | 1142.8 | B2 protein |
| 187 | c47991_g2 | 900.7 | 578.3 | 1013.6 | 570.8 | 652.2 | Uncharacterized protein |
| 188 | c47723_g2 | 698.6 | 1708.3 | 1024.6 | 2276.5 | 1957.4 | Root allergen protein |
| 189 | c45442_g1 | 2224.7 | 1355.4 | 16.2 | 35.9 | 10.3 | 1-aminocyclopropane-1-carboxylate oxidase 3 |
| 190 | c35664_g1 | 3258.8 | 1814.1 | 1.1 | 0.5 | 0.8 | Photosystem I reaction center subunit V, chloroplastic |
| 191 | c47425_g2 | 1731.3 | 1134.1 | 1712.0 | 985.7 | 842.1 | Eukaryotic translation initiation factor 5A |
| 192 | c31982_g1 | 1013.0 | 471.0 | 32.1 | 20.6 | 17.5 | Elongation factor TuA, chloroplastic |
| 193 | c30353_g1 | 55.2 | 7.5 | 1556.4 | 2261.6 | 112.6 | Induced stolen tip protein TUB8 |
| 194 | c49090_g6 | 1391.3 | 373.0 | 3.0 | 0.6 | 15.8 | Uncharacterized protein |
| 195 | c40246_g1 | 2668.5 | 918.0 | 1193.4 | 718.4 | 695.0 | Protein EARLY RESPONSIVE TO DEHYDRATION 15 |
| 196 | c42632_g6 | 3037.2 | 1623.3 | 8.1 | 2.0 | 1.4 | Chlorophyll a-b binding protein CP26, chloroplastic |
| 197 | c43923_g2 | 31.5 | 7.6 | 1742.4 | 3287.5 | 4134.0 | Beta-fructofuranosidase, soluble isoenzyme I |
| 198 | c40887_g1 | 1745.9 | 699.6 | 1526.2 | 1621.4 | 1103.7 | Protein DEHYDRATION-INDUCED 19 homolog 3 |
| 199 | c49437_g1 | 1608.4 | 889.0 | 61.8 | 49.6 | 58.6 | Protein CURVATURE THYLAKOID 1A, chloroplastic |
| 200 | c42921_g1 | 1203.8 | 390.7 | 32.5 | 22.4 | 6.1 | 30S ribosomal protein S31, chloroplastic |
| 201 | c41350_g1 | 23.2 | 3.5 | 684.2 | 1431.9 | 43.8 | 5'-methylthioadenosine/S-adenosylhomocysteine nucleosidase |
| 202 | c51640_g1 | 851.1 | 1153.2 | 749.8 | 289.5 | 741.8 | ABC transporter G family member 29 |
| 203 | c22867_g1 | 3819.6 | 2682.4 | 0.7 | 0.0 | 0.4 | Ribulose bisphosphate carboxylase small chain, chloroplastic |
| 204 | c46299_g1 | 1897.6 | 1395.6 | 5.9 | 2.4 | 26.5 | Probable glutathione S-transferase |
| 205 | c48772_g2 | 1243.3 | 463.0 | 1879.2 | 759.5 | 1319.7 | Leucine rich repeat protein |
| 206 | c53206_g1 | 1404.4 | 192.6 | 989.2 | 377.8 | 696.7 | IAA-amino acid hydrolase ILR1 |
| 207 | c45496_g1 | 1550.4 | 360.8 | 149.6 | 47.6 | 54.7 | RING-H2 finger protein ATL5 |
| 208 | c46376_g5 | 3363.9 | 28.7 | 3.4 | 0.5 | 6.5 | Pathogenesis-related protein PR-1 type |
| 209 | c33507_g1 | 5875.7 | 1506.2 | 829.0 | 322.8 | 828.3 | Peptidyl-prolyl cis-trans isomerase |
| 210 | c39851_g1 | 1817.8 | 1241.5 | 789.0 | 465.1 | 430.1 | Cysteine proteinase 15A |
| 211 | c45460_g2 | 1018.3 | 525.4 | 565.0 | 211.4 | 707.9 | Geraniol 8-hydroxylase |
| 212 | c27329_g1 | 1580.5 | 744.7 | 76.0 | 45.8 | 63.1 | Hydroquinone glucosyltransferase |
| 213 | c56521_g1 | 2080.3 | 1058.6 | 3.9 | 2.2 | 1.8 | Photosystem I reaction center subunit II, chloroplastic |
| 214 | c50680_g3 | 2104.2 | 462.1 | 246.7 | 107.1 | 145.3 | Probable phospholipid hydroperoxide glutathione peroxidase |
| 215 | c45083_g1 | 1547.2 | 514.0 | 1.4 | 2.1 | 2.8 | EG45-like domain containing protein |
| 216 | c43725_g7 | 13.9 | 2.3 | 2480.1 | 1697.5 | 1681.1 | Tubulin alpha-3 chain |
| 217 | c50912_g2 | 1467.8 | 824.5 | 0.2 | 0.2 | 0.8 | 16kda membrane protein |
| 218 | c48503_g3 | 979.0 | 863.5 | 1012.5 | 859.0 | 705.3 | ADP-ribosylation factor 1 |
| 219 | c52373_g1 | 1985.3 | 875.6 | 0.3 | 1.0 | 0.5 | Photosystem II 5 kDa protein, chloroplastic |
| 220 | c39486_g1 | 2348.9 | 657.2 | 981.1 | 441.8 | 286.0 | Ubiquitin-40S ribosomal protein S27a-1 |
| 221 | c38723_g1 | 1773.3 | 616.3 | 1072.9 | 355.8 | 348.7 | Phosphoprotein ECPP44 |
| 222 | c49777_g1 | 795.6 | 2186.8 | 2403.6 | 1455.9 | 1694.2 | Omega-6 fatty acid desaturase, endoplasmic reticulum |
| 223 | c43377_g1 | 1495.2 | 1418.6 | 3.0 | 8.4 | 3.6 | Chlorophyll a-b binding protein 36, chloroplastic |
| 224 | c48920_g2 | 47.0 | 125.9 | 499.7 | 1046.4 | 840.0 | FAM10 family protein At4g22670 |
| 225 | c18698_g1 | 2538.6 | 1045.5 | 0.0 | 0.8 | 0.6 | Chlorophyll a-b binding protein 3, chloroplastic |
| 226 | c50221_g1 | 1637.1 | 754.5 | 15.5 | 15.7 | 5.0 | Phosphoglycerate kinase, chloroplastic |
| 227 | c32146_g1 | 13.5 | 5.8 | 2076.3 | 520.8 | 1110.1 | Uncharacterized protein |
| 228 | c40145_g1 | 1321.0 | 107.5 | 0.4 | 0.4 | 23.4 | Miraculin |
| 229 | c44479_g1 | 3939.9 | 2070.5 | 1.1 | 0.7 | 1.2 | Chlorophyll a-b binding protein CP29Uncharacterized protein2, chloroplastic |
| 230 | c46802_g1 | 2581.0 | 2341.7 | 6.8 | 14.0 | 0.8 | Uncharacterized protein |
| 231 | c53007_g1 | 1033.4 | 637.9 | 230.4 | 167.0 | 301.8 | Poly(A) polymerase |
| 232 | c40436_g1 | 1362.8 | 811.4 | 32.9 | 30.0 | 39.3 | GILT-like protein F37H8Uncharacterized protein5 |
| 233 | c40661_g1 | 1440.0 | 1644.5 | 1407.1 | 783.4 | 680.3 | Stem-specific protein TSJT1 |
| 234 | c42178_g1 | 796.3 | 261.9 | 1088.9 | 532.5 | 228.1 | Cold-regulated 413 plasma membrane protein 2 |
| 235 | c33719_g1 | 2187.8 | 101.7 | 0.2 | 0.5 | 0.2 | Pelargonidin 3-O-(6-caffeoylglucoside) 5-O-(6-O-malonylglucoside) 4'''-malonyltransferase |
| 236 | c49207_g1 | 2130.9 | 781.7 | 5.1 | 4.0 | 3.5 | Glyceraldehyde-3-phosphate dehydrogenase GAPA1, chloroplastic |
| 237 | c35380_g1 | 1181.5 | 141.2 | 401.9 | 120.9 | 240.4 | Uncharacterized protein |
| 238 | c52068_g1 | 2142.4 | 373.4 | 488.6 | 235.9 | 109.7 | Uncharacterized protein |
| 239 | c23671_g1 | 658.9 | 1103.7 | 699.1 | 554.9 | 489.2 | Delta(12) fatty acid dehydrogenase |
| 240 | c34062_g1 | 14.1 | 58.7 | 5635.3 | 846.6 | 1736.4 | Uncharacterized protein |
| 241 | c49261_g1 | 1250.3 | 758.0 | 2694.4 | 1321.2 | 2110.3 | Geraniol dehydrogenase 1 |
| 242 | c48380_g1 | 1337.1 | 300.2 | 10.6 | 3.1 | 9.6 | Glucan endo-1,3-beta-glucosidase |
| 243 | c53932_g2 | 1145.2 | 284.6 | 13.2 | 5.3 | 14.9 | Putative 12-oxophytodienoate reductase 11 |
| 244 | c36012_g1 | 2746.8 | 1130.4 | 1695.9 | 495.6 | 1062.9 | Protein TIFY 10A |
| 245 | c38203_g2 | 3204.7 | 1778.6 | 1.2 | 0.7 | 0.8 | Photosystem I reaction center subunit IV A, chloroplastic |
| 246 | c30151_g1 | 468.2 | 213.4 | 1060.7 | 902.2 | 785.4 | Serine/arginine-rich splicing factor RS2Z32 |
| 247 | c52112_g4 | 13016.9 | 3246.4 | 252.8 | 80.0 | 152.5 | Metallothionein-like protein type 3 |
| 248 | c26157_g1 | 3572.4 | 1776.6 | 1587.3 | 730.2 | 1282.9 | JAZ1 |
| 249 | c31603_g1 | 1471.3 | 653.8 | 0.2 | 1.8 | 0.3 | Glycine cleavage system H protein, mitochondrial |
| 250 | c27305_g1 | 1324.8 | 647.3 | 303.1 | 154.6 | 209.5 | Ubiquitin-like protein 5 |
| 251 | c46843_g1 | 2496.7 | 1791.0 | 16.3 | 21.5 | 0.7 | Chlorophyll a-b binding protein 7, chloroplastic |
| 252 | c53663_g1 | 1987.7 | 1137.2 | 1930.5 | 953.3 | 1417.7 | Glyceraldehyde-3-phosphate dehydrogenase, cytosolic |
| 253 | c47723_g3 | 1811.2 | 2664.2 | 27786.8 | 6864.0 | 8332.7 | Root allergen protein |
| 254 | c34360_g1 | 181.1 | 26.4 | 2663.2 | 1063.6 | 831.3 | Putative uncharacterized protein |
| 255 | c53561_g1 | 2612.5 | 1025.3 | 3.1 | 2.9 | 3.9 | Linoleate 13S-lipoxygenase 2-1, chloroplastic |
| 256 | c51924_g2 | 6434.9 | 4242.9 | 11.5 | 9.3 | 10.3 | Ribulose bisphosphate carboxylase/oxygenase activase 1, chloroplastic |
| 257 | c38975_g1 | 1302.8 | 317.9 | 79.8 | 64.2 | 121.0 | Uncharacterized protein |
| 258 | c43148_g1 | 2203.9 | 1882.4 | 4.2 | 7.5 | 3.5 | Ferredoxin--NADP reductase, leaf-type isozyme, chloroplastic |
| 259 | c45778_g2 | 1357.7 | 997.3 | 3824.4 | 2250.7 | 1946.9 | Ubiquitin-conjugating enzyme E2 10 |
| 260 | c48487_g1 | 2256.1 | 1199.9 | 0.3 | 0.7 | 0.4 | Phosphoribulokinase |
| 261 | c53501_g3 | 1585.0 | 878.2 | 31.3 | 15.8 | 10.6 | 30S ribosomal protein S12, chloroplastic |
| 262 | c38509_g1 | 4.3 | 1.4 | 4673.7 | 1632.5 | 2615.1 | 17Uncharacterized protein8 kDa class I heat shock protein |
| 263 | c53663_g5 | 1125.3 | 1158.1 | 1329.2 | 790.0 | 1255.8 | Uncharacterized protein |
| 264 | c39029_g1 | 1105.4 | 1237.5 | 637.5 | 678.7 | 301.7 | Peptidyl-prolyl cis-trans isomerase |
| 265 | c44708_g2 | 1725.4 | 564.7 | 443.6 | 160.0 | 208.7 | Thioredoxin H-type |
| 266 | c53455_g3 | 444.3 | 484.2 | 1315.6 | 842.8 | 904.6 | Enolase |
| 267 | c25218_g1 | 1666.6 | 353.0 | 0.2 | 0.7 | 0.4 | Uncharacterized protein |
| 268 | c41101_g1 | 1433.8 | 980.7 | 0.3 | 0.3 | 0.3 | ATP synthase gamma chain, chloroplastic |
| 269 | c32940_g1 | 1131.4 | 526.6 | 405.3 | 164.2 | 122.4 | Uncharacterized protein |
| 270 | c45610_g2 | 5094.7 | 2218.4 | 10.0 | 7.1 | 8.0 | Photosystem I reaction center subunit VI, chloroplastic |
| 271 | c39128_g1 | 4888.2 | 1906.6 | 3503.3 | 2882.4 | 1591.7 | Metallothionein-like protein 1 |
| 272 | c45601_g2 | 987.6 | 310.9 | 3411.0 | 1469.0 | 1236.6 | Ubiquitin-conjugating enzyme E2 7 |
| 273 | c53630_g1 | 1495.2 | 1787.1 | 1902.7 | 1040.6 | 1051.1 | Uncharacterized protein |
| 274 | c46885_g1 | 1365.0 | 839.5 | 373.4 | 119.7 | 861.7 | 1-aminocyclopropane-1-carboxylate oxidase 3 |
| 275 | c52915_g5 | 1995.2 | 1958.0 | 3817.6 | 4691.7 | 3326.4 | Heat shock cognate 70 kDa protein 1 |
| 276 | c45922_g3 | 4108.9 | 2326.7 | 3774.1 | 1941.2 | 1671.3 | Translation machinery-associated protein 7 |
| 277 | c52032_g1 | 2982.2 | 1692.9 | 69.1 | 42.4 | 53.7 | Clavaminate synthase-like protein At3g21360 |
| 278 | c52602_g1 | 934.6 | 788.5 | 3549.7 | 2360.6 | 2023.2 | DnaJ protein homolog ANJ1 |
| 279 | c42935_g1 | 1637.1 | 2945.9 | 31.8 | 40.7 | 15.4 | Uncharacterized protein |
| 280 | c48803_g2 | 1035.0 | 797.4 | 465.5 | 346.8 | 382.4 | Uncharacterized protein |
| 281 | c35286_g2 | 1424.5 | 922.5 | 511.4 | 402.4 | 292.7 | Cysteine proteinase 15A |
| 282 | c45351_g1 | 773.6 | 1016.2 | 127.8 | 113.1 | 39.7 | Copper transport protein CCH |
| 283 | c41446_g1 | 385.1 | 221.4 | 1241.8 | 421.2 | 587.2 | NHP2-like protein 1 homolog |
