# Supplementary material for: Differential Gene Expression between Leaf and Rhizome in Atractylodes lancea: A Comparative Transcriptome Analysis
Source: Front Plant Sci. 2016 Mar 30;7:348. doi: 10.3389/fpls.2016.00348 (PMC4811964; doi:10.3389/fpls.2016.00348)
Supplement: Supplementary file 5 [file Table5.docx]

**Supplementary Table 5** List of leaf-specific expressed genes in *A. lancea* detected by transcriptome sequencing.

| No. | Gene ID | L1  Exp | L2  Exp | R1  Exp | R2  Exp | R3  Exp | L1  FPKM | L2  FPKM | R1  FPKM | R2  FPKM | R3  FPKM | logFC | PValue | FDR | Blast_hit |
| --- | --- | --- | --- | --- | --- | --- | --- | --- | --- | --- | --- | --- | --- | --- | --- |
| 1 | c42589_g1 | 388 | 729 | 0 | 0 | 0 | 33.285 | 68.012 | 0 | 0 | 0 | -12.2489 | 1.65E-17 | 6.22E-15 | (S)-N-methylcoclaurine 3'-hydroxylase |
| 2 | c22795_g1 | 13 | 13 | 0 | 0 | 0 | 1.708 | 1.863 | 0 | 0 | 0 | -6.82674 | 0.003435 | 0.058318 | 40S ribosomal protein S18 |
| 3 | c32301_g2 | 57 | 20 | 0 | 0 | 0 | 13.995 | 5.412 | 0 | 0 | 0 | -8.36981 | 0.000111 | 0.003435 | Acanthoscurrin-1 |
| 4 | c50355_g3 | 65 | 167 | 0 | 0 | 0 | 10.193 | 36.386 | 0 | 0 | 0 | -9.98665 | 7.66E-08 | 5.70E-06 | Acyltransferase-like protein |
| 5 | c39634_g5 | 28 | 24 | 0 | 0 | 0 | 5.082 | 4.771 | 0 | 0 | 0 | -7.81805 | 0.00041 | 0.010427 | Aldehyde dehydrogenase |
| 6 | c39634_g4 | 51 | 35 | 0 | 0 | 0 | 6.876 | 5.04 | 0 | 0 | 0 | -8.53796 | 4.47E-05 | 0.001584 | Aldehyde dehydrogenase |
| 7 | c53406_g1 | 328 | 100 | 0 | 0 | 0 | 19.646 | 6.178 | 0 | 0 | 0 | -10.839 | 2.76E-10 | 3.33E-08 | Aldehyde dehydrogenase |
| 8 | c39733_g1 | 114 | 88 | 0 | 0 | 0 | 12.258 | 10.835 | 0 | 0 | 0 | -9.76941 | 1.26E-07 | 8.91E-06 | aldehyde dehydrogenase |
| 9 | c5871_g1 | 65 | 69 | 0 | 0 | 0 | 34.225 | 40.681 | 0 | 0 | 0 | -9.18307 | 3.10E-06 | 0.000156 | AMT1 |
| 10 | c32798_g1 | 131 | 16 | 0 | 0 | 1 | 13.966 | 1.863 | 0 | 0 | 0.081 | -7.5175 | 4.18E-05 | 0.001496 | Aspartate, glycine, lysine and serine  rich protein |
| 11 | c28702_g1 | 100 | 27 | 0 | 0 | 0 | 74.358 | 22.653 | 0 | 0 | 0 | -9.08692 | 1.03E-05 | 0.000444 | ATP synthase subunit 9 |
| 12 | c54298_g1 | 149 | 40 | 0 | 0 | 0 | 87.014 | 26.224 | 0 | 0 | 0 | -9.65948 | 7.17E-07 | 4.24E-05 | ATP synthase subunit 9 |
| 13 | c43299_g1 | 24 | 23 | 0 | 0 | 0 | 7.417 | 7.855 | 0 | 0 | 0 | -7.67457 | 0.000611 | 0.014518 | CC-NBS-LRR RGA |
| 14 | c50193_g1 | 1183 | 718 | 1 | 1 | 0 | 39.008 | 24.133 | 0.032 | 0.032 | 0 | -10.3698 | 3.04E-21 | 1.85E-18 | cellulose synthase |
| 15 | c67184_g1 | 1171 | 499 | 0 | 1 | 0 | 127.246 | 59.081 | 0 | 0.104 | 0 | -10.9783 | 1.95E-19 | 1.00E-16 | Chaperone protein DnaJ |
| 16 | c48779_g6 | 3680 | 1289 | 1 | 0 | 1 | 469.294 | 193.522 | 0.168 | 0 | 0.113 | -11.7709 | 1.12E-27 | 1.38E-24 | Chaperone protein dnaJ |
| 17 | c48303_g8 | 217 | 149 | 0 | 0 | 0 | 82.487 | 62.941 | 0 | 0 | 0 | -10.6244 | 2.93E-10 | 3.51E-08 | Chlorophyll a-b binding protein |
| 18 | c34786_g1 | 87 | 11 | 0 | 0 | 0 | 4.072 | 0.559 | 0 | 0 | 0 | -8.70745 | 8.57E-05 | 0.002757 | Chlorophyll a-b binding protein |
| 19 | c32605_g1 | 88 | 11 | 0 | 0 | 1 | 11.418 | 1.563 | 0 | 0 | 0.097 | -6.95236 | 0.000279 | 0.00753 | Chlorophyll a-b binding protein |
| 20 | c43373_g1 | 315 | 28 | 0 | 0 | 1 | 25.683 | 2.473 | 0 | 0 | 0.057 | -8.72959 | 2.79E-07 | 1.82E-05 | Chlorophyll a-b binding protein |
| 21 | c11694_g1 | 1824 | 1676 | 1 | 1 | 1 | 449.477 | 455.294 | 0.337 | 0.287 | 0.226 | -10.773 | 2.95E-28 | 3.79E-25 | chloroplast PsbO1 precursor |
| 22 | c41524_g1 | 385 | 47 | 0 | 0 | 0 | 30.808 | 4.088 | 0 | 0 | 0 | -10.8445 | 4.07E-09 | 3.91E-07 | chloroplast rubisco small subunit |
| 23 | c2627_g1 | 82 | 74 | 0 | 0 | 0 | 3.758 | 3.674 | 0 | 0 | 0 | -9.39951 | 1.03E-06 | 5.86E-05 | conserved hypothetical protein |
| 24 | c21545_g1 | 84 | 65 | 0 | 1 | 1 | 6.549 | 5.506 | 0 | 0.072 | 0.057 | -6.75568 | 1.24E-05 | 0.000522 | conserved hypothetical protein |
| 25 | c49830_g1 | 423 | 13 | 1 | 0 | 0 | 42.695 | 1.428 | 0.116 | 0 | 0 | -8.9737 | 5.93E-07 | 3.61E-05 | conserved hypothetical protein |
| 26 | c20294_g1 | 960 | 682 | 0 | 0 | 0 | 79.639 | 61.368 | 0 | 0 | 0 | -12.7897 | 1.52E-21 | 9.68E-19 | conserved hypothetical protein |
| 27 | c43857_g2 | 113 | 25 | 0 | 0 | 0 | 72.521 | 14.509 | 0 | 0 | 0 | -9.20455 | 7.87E-06 | 0.000352 | conserved hypothetical protein |
| 28 | c76949_g1 | 48 | 23 | 0 | 0 | 0 | 3.915 | 2.039 | 0 | 0 | 0 | -8.25715 | 0.000133 | 0.003995 | conserved hypothetical protein |
| 29 | c78710_g1 | 16 | 12 | 0 | 0 | 0 | 2.15 | 1.759 | 0 | 0 | 0 | -6.92851 | 0.002896 | 0.05092 | retroviral aspartyl proteases |
| 30 | c46990_g1 | 188 | 21 | 1 | 0 | 1 | 21.013 | 2.556 | 0.126 | 0 | 0.081 | -7.18698 | 1.12E-05 | 0.000477 | CsbD-like protein |
| 31 | c25747_g1 | 33 | 23 | 0 | 0 | 0 | 2.306 | 1.749 | 0 | 0 | 0 | -7.9214 | 0.000315 | 0.008368 | Cyclopentanone 1,2-monooxygenase |
| 32 | c1623_g1 | 22 | 16 | 1 | 0 | 0 | 3.516 | 2.794 | 0.179 | 0 | 0 | -5.47516 | 0.004112 | 0.06747 | Cyclopentanone 1,2-monooxygenase |
| 33 | c48162_g3 | 16 | 148 | 0 | 0 | 0 | 2.676 | 27.414 | 0 | 0 | 0 | -9.49805 | 6.49E-06 | 0.000297 | Cysteine-rich receptor-like kinase |
| 34 | c36379_g1 | 52 | 16 | 0 | 0 | 0 | 6.876 | 2.308 | 0 | 0 | 0 | -8.18958 | 0.00021 | 0.005919 | Cytochrome c |
| 35 | c21794_g1 | 48 | 82 | 0 | 0 | 0 | 5.31 | 10.276 | 0 | 0 | 0 | -9.14659 | 4.46E-06 | 0.000213 | cytochrome P450 |
| 36 | c21794_g2 | 23 | 55 | 0 | 0 | 0 | 2.491 | 6.489 | 0 | 0 | 0 | -8.4158 | 9.14E-05 | 0.002907 | cytochrome P450 |
| 37 | c28589_g1 | 561 | 18 | 0 | 0 | 1 | 24.743 | 0.859 | 0 | 0 | 0.032 | -9.48227 | 6.31E-08 | 4.81E-06 | Cytochrome P450 94B3 |
| 38 | c27034_g1 | 47 | 46 | 0 | 0 | 0 | 51.736 | 47.449 | 0 | 0 | 0 | -8.65597 | 2.83E-05 | 0.001071 | D-galacturonic acid reductase 1 |
| 39 | c52037_g3 | 85 | 68 | 0 | 0 | 1 | 12.058 | 10.535 | 0 | 0 | 0.105 | -7.58906 | 5.09E-06 | 0.00024 | DNA ligase |
| 40 | c51923_g1 | 84 | 65 | 0 | 0 | 1 | 6.834 | 5.754 | 0 | 0 | 1.051 | -7.55073 | 6.32E-06 | 0.00029 | E3 ubiquitin-protein ligase RNF12-B |
| 41 | c43554_g1 | 30 | 31 | 1 | 0 | 0 | 7.246 | 9.624 | 1.178 | 0 | 0 | -6.16452 | 0.000806 | 0.018216 | Elongation factor 1-alpha |
| 42 | c19526_g1 | 65 | 15 | 0 | 0 | 0 | 3.588 | 0.9 | 0 | 0 | 0 | -8.42026 | 0.000124 | 0.003763 | Enolase 1 |
| 43 | c67271_g1 | 12 | 43 | 1 | 0 | 1 | 2.62 | 10.318 | 0.242 | 0 | 0.162 | -5.30637 | 0.002685 | 0.04813 | ethylene responsive transcription factor |
| 44 | c12729_g1 | 461 | 16 | 0 | 0 | 0 | 65.132 | 2.473 | 0 | 0 | 0 | -10.9825 | 5.47E-08 | 4.22E-06 | Ethylene-responsive transcription factor |
| 45 | c28429_g1 | 43 | 14 | 0 | 0 | 0 | 4.015 | 1.428 | 0 | 0 | 0 | -7.93665 | 0.000414 | 0.010534 | Expressed protein |
| 46 | c54176_g2 | 82 | 25 | 0 | 0 | 0 | 5.296 | 1.759 | 0 | 0 | 0 | -8.84156 | 2.37E-05 | 0.000917 | F15O4.13 |
| 47 | c43579_g2 | 14 | 16 | 0 | 0 | 0 | 2.62 | 3.27 | 0 | 0 | 0 | -7.03349 | 0.002485 | 0.045312 | FAD2-8 protein |
| 48 | c51988_g3 | 11 | 80 | 0 | 0 | 0 | 2.506 | 20.056 | 0 | 0 | 0 | -8.6483 | 0.000103 | 0.00323 | Farnesylcysteine lyase |
| 49 | c50541_g2 | 112 | 115 | 0 | 0 | 0 | 6.848 | 7.637 | 0 | 0 | 0 | -9.942 | 4.25E-08 | 3.34E-06 | F-box/kelch-repeat protein |
| 50 | c45322_g2 | 11 | 24 | 0 | 0 | 0 | 0.925 | 3.343 | 0 | 0 | 0 | -7.26344 | 0.001752 | 0.034347 | flowering locus T |
| 51 | c31401_g2 | 47 | 17 | 0 | 0 | 0 | 10.649 | 3.55 | 0 | 0 | 0 | -8.10433 | 0.000243 | 0.006682 | GDSL esterase/lipase CPRD49 |
| 52 | c51862_g1 | 156 | 215 | 0 | 0 | 0 | 28.174 | 42.544 | 0 | 0 | 0 | -10.6547 | 2.29E-10 | 2.83E-08 | GDSL esterase/lipase EXL2 |
| 53 | c34928_g1 | 73 | 27 | 0 | 0 | 0 | 3.104 | 1.242 | 0 | 0 | 0 | -8.74646 | 2.92E-05 | 0.001101 | General alpha-glucoside permease |
| 54 | c18385_g1 | 26 | 52 | 0 | 1 | 0 | 22.294 | 50.492 | 0 | 0.79 | 0 | -6.57972 | 0.000304 | 0.008109 | geraniol 10-hydroxylase |
| 55 | c32973_g1 | 372 | 229 | 0 | 0 | 0 | 27.405 | 18.855 | 0 | 0 | 0 | -11.3379 | 3.55E-13 | 7.01E-11 | germin like protein |
| 56 | c71819_g1 | 29 | 12 | 0 | 0 | 0 | 2.363 | 1.066 | 0 | 0 | 0 | -7.46663 | 0.001142 | 0.024259 | Gibberellin 2-beta-dioxygenase 8 |
| 57 | c32204_g1 | 42 | 28 | 0 | 0 | 0 | 11.816 | 8.351 | 0 | 0 | 0 | -8.24145 | 0.000126 | 0.003805 | Glucose-repressible gene protein |
| 58 | c43857_g1 | 52 | 18 | 0 | 0 | 0 | 16.699 | 6.861 | 0 | 0 | 0 | -8.23261 | 0.000175 | 0.00506 | Glucose-repressible gene protein |
| 59 | c71394_g1 | 33 | 18 | 0 | 0 | 0 | 3.232 | 1.925 | 0 | 0 | 0 | -7.78353 | 0.000504 | 0.012386 | Glutamine synthetase |
| 60 | c76087_g1 | 46 | 14 | 0 | 0 | 0 | 11.589 | 3.891 | 0 | 0 | 0 | -8.00958 | 0.000343 | 0.008991 | Glutaredoxin |
| 61 | c28577_g1 | 213 | 143 | 0 | 0 | 0 | 54.085 | 40.008 | 0 | 0 | 0 | -10.5841 | 4.20E-10 | 4.86E-08 | glutathione S-transferase 1 |
| 62 | c28577_g2 | 46 | 27 | 0 | 0 | 0 | 40.389 | 26.855 | 0 | 0 | 0 | -8.29993 | 0.000109 | 0.003394 | glutathione S-transferase 1 |
| 63 | c43790_g7 | 59 | 175 | 0 | 0 | 1 | 8.385 | 27.186 | 0 | 0 | 0.105 | -8.2177 | 4.03E-07 | 2.53E-05 | glycosyltransferase UGT72B11 |
| 64 | c45838_g6 | 663 | 448 | 0 | 1 | 1 | 38.681 | 24.651 | 0 | 0.048 | 0.097 | -9.63808 | 1.40E-16 | 4.74E-14 | High affinity nitrate transporter |
| 65 | c43521_g4 | 66 | 53 | 0 | 0 | 0 | 12.258 | 14.819 | 0 | 0 | 0 | -9.00779 | 7.13E-06 | 0.000322 | Histone H4 variant TH011 |
| 66 | c49427_g2 | 62 | 20 | 0 | 0 | 0 | 13.753 | 4.885 | 0 | 0 | 0 | -8.45931 | 8.68E-05 | 0.00279 | hydrolase |
| 67 | c27586_g1 | 76 | 132 | 0 | 0 | 0 | 15.432 | 29.422 | 0 | 0 | 0 | -9.82398 | 1.21E-07 | 8.59E-06 | hydrophobin |
| 68 | c27855_g2 | 22 | 95 | 0 | 0 | 0 | 0.655 | 3.043 | 0 | 0 | 0 | -9.00601 | 1.85E-05 | 0.000739 | hypothetical protein |
| 69 | c18240_g1 | 15 | 26 | 0 | 0 | 0 | 1.965 | 3.726 | 0 | 0 | 0 | -7.48729 | 0.001048 | 0.022669 | hypothetical protein |
| 70 | c39020_g1 | 185 | 416 | 0 | 1 | 0 | 32.787 | 81.134 | 0 | 0.192 | 0 | -9.52751 | 6.14E-12 | 9.99E-10 | hypothetical protein |
| 71 | c397_g1 | 40 | 11 | 0 | 0 | 0 | 5.652 | 1.697 | 0 | 0 | 0 | -7.77507 | 0.000679 | 0.01581 | hypothetical protein |
| 72 | c39634_g1 | 59 | 15 | 0 | 0 | 0 | 6.748 | 2.008 | 0 | 0 | 0 | -8.3091 | 0.000169 | 0.0049 | hypothetical protein |
| 73 | c38845_g1 | 12 | 18 | 0 | 0 | 0 | 2.306 | 3.798 | 0 | 0 | 0 | -7.03745 | 0.002553 | 0.046292 | hypothetical protein |
| 74 | c38218_g1 | 23 | 16 | 0 | 0 | 0 | 4.584 | 3.498 | 0 | 0 | 0 | -7.40206 | 0.001161 | 0.024581 | hypothetical protein |
| 75 | c34647_g1 | 101 | 87 | 0 | 0 | 0 | 15.062 | 14.188 | 0 | 0 | 0 | -9.66761 | 2.32E-07 | 1.54E-05 | hypothetical protein |
| 76 | c30628_g1 | 40 | 13 | 0 | 0 | 0 | 7.816 | 2.784 | 0 | 0 | 0 | -7.83213 | 0.000538 | 0.013095 | hypothetical protein |
| 77 | c20393_g1 | 16 | 14 | 0 | 0 | 0 | 2.15 | 2.049 | 0 | 0 | 0 | -7.02952 | 0.002471 | 0.045136 | hypothetical protein |
| 78 | c59339_g1 | 31 | 27 | 0 | 0 | 0 | 2.264 | 2.142 | 0 | 0 | 0 | -7.97517 | 0.000271 | 0.007365 | hypothetical protein |
| 79 | c42180_g2 | 122 | 42 | 0 | 0 | 0 | 8.101 | 3.032 | 0 | 0 | 0 | -9.45789 | 1.44E-06 | 7.90E-05 | hypothetical protein |
| 80 | c24025_g1 | 12 | 90 | 0 | 0 | 0 | 1.367 | 11.208 | 0 | 0 | 0 | -8.81277 | 6.23E-05 | 0.002097 | hypothetical protein GLRG_09422 |
| 81 | c22718_g1 | 189 | 177 | 1 | 0 | 0 | 19.348 | 19.725 | 0.116 | 0 | 0 | -8.76696 | 1.52E-09 | 1.60E-07 | hypothetical protein M91_20998 |
| 82 | c53404_g2 | 46 | 31 | 0 | 0 | 0 | 24.971 | 18.866 | 0 | 0 | 0 | -8.37867 | 8.22E-05 | 0.00266 | hypothetical protein MTR_4g063500 |
| 83 | c29851_g1 | 23.41 | 103.22 | 0 | 0 | 0 | 0.769 | 3.581 | 0 | 0 | 0 | -9.11308 | 1.26E-05 | 0.000532 | hypothetical protein MTR_5g065250 |
| 84 | c44928_g2 | 24 | 45 | 0 | 0 | 0 | 3.346 | 6.841 | 0 | 0 | 0 | -8.23622 | 0.000146 | 0.004324 | hypothetical protein |
| 85 | c44928_g1 | 123 | 111 | 0 | 0 | 0 | 11.617 | 10.204 | 0 | 0 | 0 | -9.98376 | 3.31E-08 | 2.67E-06 | hypothetical protein |
| 86 | c16975_g1 | 22 | 35 | 0 | 0 | 0 | 2.079 | 3.601 | 0 | 0 | 0 | -7.95923 | 0.000309 | 0.008239 | hypothetical protein |
| 87 | c31719_g1 | 45 | 20 | 0 | 0 | 0 | 38.852 | 19.528 | 0 | 0 | 0 | -8.12921 | 0.000206 | 0.005813 | hypothetical protein NFIA_032130 |
| 88 | c36124_g1 | 159 | 196 | 0 | 0 | 0 | 9.937 | 13.288 | 0 | 0 | 0 | -10.5894 | 3.66E-10 | 4.32E-08 | hypothetical protein |
| 89 | c48041_g2 | 31 | 35 | 1 | 1 | 1 | 10.037 | 10.266 | 0.295 | 0.247 | 0.194 | -5.04536 | 0.001275 | 0.026585 | hypothetical protein |
| 90 | c39448_g1 | 49 | 90 | 0 | 1 | 0 | 9.353 | 15.233 | 0 | 0.184 | 0 | -7.41135 | 1.25E-05 | 0.000527 | hypothetical protein |
| 91 | c66283_g1 | 17 | 23 | 0 | 0 | 0 | 10.179 | 15.471 | 0 | 0 | 0 | -7.44831 | 0.001065 | 0.022969 | hypothetical protein |
| 92 | c46714_g1 | 82 | 318 | 0 | 1 | 0 | 3.217 | 13.526 | 0 | 0.04 | 0 | -8.94555 | 5.06E-09 | 4.79E-07 | hypothetical protein |
| 93 | c50000_g1 | 528 | 519 | 0 | 1 | 0 | 50.469 | 54 | 0 | 0.088 | 0 | -10.3171 | 8.41E-17 | 2.97E-14 | hypothetical protein |
| 94 | c40828_g1 | 77 | 16 | 0 | 0 | 0 | 4.157 | 0.942 | 0 | 0 | 0 | -8.63588 | 6.91E-05 | 0.002295 | hypothetical protein PTT_16560 |
| 95 | c43512_g3 | 59 | 18 | 0 | 0 | 0 | 11.133 | 3.726 | 0 | 0 | 0 | -8.36819 | 0.000122 | 0.003704 | hypothetical protein |
| 96 | c14863_g1 | 27 | 22 | 0 | 0 | 0 | 2.463 | 2.184 | 0 | 0 | 0 | -7.73195 | 0.000527 | 0.012869 | hypothetical protein SNOG_01331 |
| 97 | c37728_g1 | 81 | 42 | 1 | 1 | 0 | 134.692 | 80.017 | 1.757 | 1.548 | 0 | -6.39408 | 5.35E-05 | 0.001841 | hypothetical protein |
| 98 | c17291_g1 | 72 | 28 | 0 | 1 | 0 | 83.64 | 37.049 | 0 | 1.077 | 0 | -6.91367 | 0.000107 | 0.003341 | hypothetical protein VITISV_003752 |
| 99 | c35334_g1 | 16 | 78 | 0 | 1 | 1 | 3.573 | 18.949 | 0 | 0.168 | 0.129 | -6.12166 | 0.000388 | 0.00996 | hypothetical protein VITISV_008862 |
| 100 | c53835_g1 | 196 | 35 | 0 | 0 | 0 | 77.447 | 6.561 | 0 | 0 | 0 | -9.94481 | 3.19E-07 | 2.05E-05 | hypothetical protein VITISV_008862 |
| 101 | c21680_g1 | 47 | 16 | 0 | 0 | 0 | 79.711 | 31.098 | 0 | 0 | 0 | -8.08097 | 0.000267 | 0.007264 | hypothetical protein VITISV_011191 |
| 102 | c42635_g1 | 234 | 365 | 0 | 0 | 0 | 8.955 | 15.13 | 0 | 0 | 0 | -11.3474 | 3.50E-13 | 6.95E-11 | hypothetical protein VITISV_011832 |
| 103 | c47885_g1 | 25 | 53 | 0 | 0 | 1 | 2.121 | 4.895 | 0 | 0 | 0.065 | -6.64177 | 0.000301 | 0.00806 | hypothetical protein VITISV_018026 |
| 104 | c41162_g1 | 14 | 89 | 0 | 0 | 0 | 2.363 | 16.486 | 0 | 0 | 0 | -8.8257 | 5.07E-05 | 0.001759 | hypothetical protein VITISV_020406 |
| 105 | c32262_g1 | 30 | 12 | 0 | 0 | 0 | 17.397 | 6.427 | 0 | 0 | 0 | -7.50076 | 0.00107 | 0.023043 | hypothetical protein VITISV_029990 |
| 106 | c25920_g1 | 178 | 135 | 0 | 0 | 0 | 60.135 | 49.219 | 0 | 0 | 0 | -10.4004 | 1.66E-09 | 1.73E-07 | hypothetical protein VITISV_033584 |
| 107 | c54917_g1 | 32 | 14 | 0 | 1 | 0 | 3.018 | 1.438 | 0 | 0.088 | 0 | -5.79792 | 0.002513 | 0.045707 | hypothetical protein VITISV_043720 |
| 108 | c33897_g1 | 25 | 12 | 0 | 0 | 0 | 2.662 | 1.397 | 0 | 0 | 0 | -7.32138 | 0.001531 | 0.030814 | Inorganic phosphate transporter PHO84 |
| 109 | c54232_g4 | 32 | 35 | 0 | 0 | 0 | 17.796 | 21.815 | 0 | 0 | 0 | -8.18599 | 0.000145 | 0.0043 | Integrase core domain containing |
| 110 | c48303_g5 | 923 | 718 | 0 | 0 | 0 | 115.572 | 98.21 | 0 | 0 | 0 | -12.7902 | 1.16E-21 | 7.57E-19 | light-harvesting chlorophyll a/b-binding  protein (LHCP) precursor |
| 111 | c46773_g6 | 77 | 207 | 0 | 1 | 0 | 14.322 | 42.244 | 0 | 0.176 | 0 | -8.44729 | 6.39E-08 | 4.86E-06 | Lysosomal Pro-X carboxypeptidase |
| 112 | c49738_g2 | 16 | 16 | 1 | 0 | 0 | 2.135 | 2.328 | 0.147 | 0 | 0 | -5.23295 | 0.006678 | 0.09725 | Magnesium transporter NIPA3 |
| 113 | c30692_g1 | 41 | 21 | 0 | 0 | 0 | 6.122 | 2.546 | 0 | 0 | 0 | -8.06322 | 0.000237 | 0.006544 | Maltose permease MAL61 |
| 114 | c2727_g1 | 48 | 15 | 0 | 0 | 0 | 22.209 | 7.751 | 0 | 0 | 0 | -8.07998 | 0.000281 | 0.007581 | mitochondrial succinate dehydrogenase  iron sulfur subunit |
| 115 | c52692_g1 | 1563 | 3058 | 0 | 1 | 0 | 65.801 | 130.167 | 0 | 0.04 | 0 | -12.4705 | 1.54E-29 | 2.20E-26 | MLO protein 1 |
| 116 | c27180_g1 | 19 | 44 | 0 | 0 | 0 | 3.217 | 8.144 | 0 | 0 | 0 | -8.10822 | 0.000231 | 0.006415 | monosaccharide-H+ symporter |
| 117 | c25921_g1 | 72 | 116 | 0 | 1 | 1 | 11.56 | 20.677 | 0 | 0.343 | 0.267 | -7.10059 | 2.42E-06 | 0.000126 | multidrug resistance-associated protein |
| 118 | c27983_g1 | 51 | 21 | 0 | 0 | 0 | 4.613 | 2.059 | 0 | 0 | 0 | -8.27525 | 0.000135 | 0.004063 | Non-classical export protein 2 |
| 119 | c56075_g1 | 16 | 13 | 0 | 0 | 0 | 1.751 | 1.542 | 0 | 0 | 0 | -6.9799 | 0.002661 | 0.047803 | O-acyltransferase WSD1 |
| 120 | c33483_g1 | 203 | 21 | 1 | 0 | 0 | 15.005 | 1.635 | 0.063 | 0 | 0 | -8.0182 | 4.77E-06 | 0.000226 | Opsin-1 |
| 121 | c47499_g2 | 12 | 60 | 0 | 0 | 0 | 1.068 | 5.785 | 0 | 0 | 0 | -8.30849 | 0.000215 | 0.006024 | ORF I polyprotein |
| 122 | c13870_g1 | 199 | 139 | 0 | 0 | 1 | 159.45 | 125.913 | 0 | 0 | 0.574 | -8.72053 | 4.04E-09 | 3.89E-07 | Os02g0698000 |
| 123 | c34595_g1 | 26 | 118 | 0 | 0 | 0 | 6.805 | 34.068 | 0 | 0 | 0 | -9.30555 | 5.69E-06 | 0.000264 | Os08g0455800 |
| 124 | c48299_g2 | 256 | 52 | 0 | 0 | 0 | 45.244 | 10.566 | 0 | 0 | 0 | -10.3606 | 2.19E-08 | 1.83E-06 | Pathogen-related protein |
| 125 | c32329_g1 | 172 | 230 | 0 | 0 | 0 | 11.617 | 16.879 | 0 | 0 | 0 | -10.77 | 9.12E-11 | 1.20E-08 | Pc12g07100 |
| 126 | c35197_g1 | 75 | 11 | 0 | 0 | 0 | 13.511 | 2.173 | 0 | 0 | 0 | -8.52052 | 0.000131 | 0.003939 | Pc21g07700 |
| 127 | c42788_g1 | 2536 | 888 | 1 | 0 | 0 | 95.357 | 40.215 | 0.042 | 0 | 0 | -11.9941 | 4.63E-25 | 4.22E-22 | Pectinesterase-1 precursor |
| 128 | c22954_g1 | 65 | 29 | 0 | 0 | 0 | 7.901 | 3.839 | 0 | 0 | 0 | -8.65982 | 3.58E-05 | 0.001313 | Peptidyl-prolyl cis-trans isomerase |
| 129 | c43932_g1 | 2056 | 2489 | 1 | 0 | 0 | 127.787 | 147.232 | 0.032 | 0 | 0 | -12.4247 | 2.49E-31 | 4.32E-28 | phosphoglucomutase |
| 130 | c25118_g2 | 438 | 545 | 0 | 1 | 0 | 51.536 | 69.916 | 0 | 0.112 | 0 | -10.2297 | 3.64E-16 | 1.16E-13 | phosphoribulose kinase |
| 131 | c7001_g1 | 50 | 57 | 0 | 1 | 0 | 24.117 | 30.715 | 0 | 0.447 | 0 | -7.02712 | 5.14E-05 | 0.00178 | photosystem I reaction center subunit |
| 132 | c31682_g1 | 70 | 16 | 0 | 0 | 0 | 7.161 | 1.78 | 0 | 0 | 0 | -8.52419 | 9.05E-05 | 0.002883 | Photosystem II 10 kDa polypeptide |
| 133 | c38410_g2 | 705 | 491 | 0 | 0 | 0 | 81.362 | 61.782 | 0 | 0 | 0 | -12.3322 | 1.09E-18 | 4.99E-16 | Photosystem II 11 kDa protein |
| 134 | c26723_g1 | 49 | 67 | 0 | 1 | 0 | 27.249 | 41.768 | 0 | 0.511 | 0 | -7.14628 | 3.40E-05 | 0.001257 | phytoene synthase |
| 135 | c26723_g3 | 51 | 90 | 0 | 1 | 0 | 18.963 | 37.162 | 0 | 0.343 | 0 | -7.43141 | 1.11E-05 | 0.000472 | phytoene synthase |
| 136 | c49505_g1 | 38 | 249 | 0 | 0 | 0 | 0.997 | 7.079 | 0 | 0 | 0 | -10.3025 | 7.38E-08 | 5.53E-06 | pol protein |
| 137 | c44032_g1 | 421 | 89 | 0 | 0 | 0 | 141.326 | 20.894 | 0 | 0 | 0 | -11.088 | 1.16E-10 | 1.49E-08 | polyprotein |
| 138 | c52084_g2 | 123.97 | 69.05 | 0 | 0 | 0 | 6.734 | 4.067 | 0 | 0 | 0 | -9.69885 | 2.39E-07 | 1.58E-05 | polyprotein |
| 139 | c42180_g1 | 358 | 85 | 0 | 0 | 1 | 29.883 | 6.271 | 0 | 0 | 0.057 | -9.0978 | 2.13E-09 | 2.16E-07 | predicted protein |
| 140 | c57856_g1 | 19 | 43 | 0 | 0 | 1 | 2.135 | 5.268 | 0 | 0 | 0.081 | -6.31479 | 0.000815 | 0.018383 | predicted protein |
| 141 | c32176_g1 | 215 | 296 | 0 | 0 | 1 | 16.842 | 25.21 | 0 | 0 | 0.057 | -9.32278 | 2.04E-11 | 3.02E-09 | ABC transporter C family member 4 |
| 142 | c75313_g1 | 27 | 23 | 0 | 0 | 0 | 39.407 | 38.384 | 0 | 0 | 0 | -7.76163 | 0.00047 | 0.011702 | ammonium transporter 1 member 1 |
| 143 | c45545_g2 | 64 | 32 | 0 | 0 | 0 | 10.008 | 5.474 | 0 | 0 | 0 | -8.69167 | 2.99E-05 | 0.001124 | ankyrin repeat-containing protein |
| 144 | c46370_g1 | 119 | 369 | 1 | 1 | 0 | 4.769 | 16.03 | 0.042 | 0.04 | 0 | -8.41846 | 6.43E-10 | 7.24E-08 | ankyrin repeat-containing protein |
| 145 | c67307_g1 | 22 | 43 | 1 | 0 | 0 | 1.481 | 3.136 | 0.074 | 0 | 0 | -6.26485 | 0.000691 | 0.016019 | auxin-induced protein 5NG4-like |
| 146 | c22321_g1 | 1489 | 1027 | 0 | 1 | 0 | 256.002 | 183.504 | 0 | 0.056 | 0 | -11.5773 | 5.71E-25 | 5.16E-22 | caffeic acid 3-O-methyltransferase |
| 147 | c33675_g1 | 14 | 639 | 0 | 0 | 0 | 2.392 | 98.396 | 0 | 0 | 0 | -11.4949 | 1.55E-08 | 1.33E-06 | cell number regulator 2-like |
| 148 | c17981_g1 | 240 | 225 | 0 | 0 | 0 | 53.8 | 55.459 | 0 | 0 | 0 | -10.9744 | 1.13E-11 | 1.73E-09 | chlorophyll a-b binding protein 13 |
| 149 | c50001_g2 | 43 | 90 | 0 | 0 | 0 | 5.538 | 12.646 | 0 | 0 | 0 | -9.18228 | 4.33E-06 | 0.000208 | CTP synthase isoform X1 |
| 150 | c33643_g1 | 123 | 42 | 1 | 1 | 1 | 16.5 | 6.22 | 0.147 | 0.128 | 0.097 | -6.3483 | 1.47E-05 | 0.000605 | cucumber peeling cupredoxin-like |
| 151 | c48556_g2 | 32 | 33 | 0 | 0 | 0 | 5.168 | 7.048 | 0 | 0 | 0 | -8.14152 | 0.000165 | 0.00479 | defensin-like protein 19-like |
| 152 | c35171_g1 | 12 | 50 | 0 | 0 | 0 | 2.264 | 8.662 | 0 | 0 | 0 | -8.0918 | 0.000344 | 0.008995 | dynein light chain LC6 |
| 153 | c15058_g1 | 19 | 11 | 0 | 0 | 0 | 3.887 | 2.463 | 0 | 0 | 0 | -7.02354 | 0.002599 | 0.046921 | E3 ubiquitin-protein ligase COP1-like |
| 154 | c55964_g1 | 14 | 31 | 0 | 1 | 0 | 4.285 | 10.494 | 0 | 0.279 | 0 | -5.78967 | 0.002569 | 0.046513 | ethylene-responsive transcription factor |
| 155 | c53067_g1 | 13 | 118 | 0 | 0 | 1 | 1.95 | 19.373 | 0 | 0 | 0.113 | -7.40262 | 7.89E-05 | 0.002569 | glutamate receptor 2.7-like |
| 156 | c47520_g1 | 116 | 1596 | 0 | 0 | 1 | 7.759 | 513.402 | 0 | 0 | 0.024 | -11.0914 | 2.28E-13 | 4.63E-11 | glutamate receptor 2.7-like |
| 157 | c43367_g2 | 59 | 47 | 0 | 0 | 0 | 6.677 | 5.795 | 0 | 0 | 0 | -8.84112 | 1.39E-05 | 0.000576 | glycine-rich protein 2-like |
| 158 | c20748_g1 | 30 | 131 | 0 | 1 | 0 | 5.268 | 25.168 | 0 | 0.16 | 0 | -7.63304 | 1.09E-05 | 0.000464 | homeobox-leucine zipper protein |
| 159 | c39793_g1 | 26 | 76 | 0 | 0 | 0 | 29.028 | 92.901 | 0 | 0 | 0 | -8.80434 | 2.63E-05 | 0.001003 | leucine-rich repeat receptor kinase |
| 160 | c52777_g1 | 121 | 31 | 0 | 0 | 0 | 42.866 | 12.18 | 0 | 0 | 0 | -9.34515 | 3.49E-06 | 0.000173 | cytochrome P450 76C4-like |
| 161 | c40508_g1 | 53 | 29 | 0 | 0 | 0 | 48.205 | 41.095 | 0 | 0 | 0 | -8.46614 | 6.40E-05 | 0.002145 | LRR receptor-like  erine/threonine kinase |
| 162 | c45316_g2 | 534 | 1302 | 0 | 0 | 0 | 26.978 | 68.405 | 0 | 0 | 0 | -12.9692 | 6.01E-21 | 3.57E-18 | lysosomal Pro-X carboxypeptidasee |
| 163 | c39783_g1 | 212 | 428 | 0 | 0 | 1 | 26.523 | 58.429 | 0 | 0 | 0.089 | -9.65167 | 1.52E-12 | 2.66E-10 | oxygen-evolving enhancer protein |
| 164 | c42145_g1 | 18 | 17 | 0 | 0 | 0 | 4.442 | 4.616 | 0 | 0 | 0 | -7.25147 | 0.001626 | 0.032398 | oxygen-evolving enhancer protein |
| 165 | c37084_g1 | 44 | 197 | 0 | 1 | 0 | 1.637 | 7.969 | 0 | 0.032 | 0 | -8.21538 | 6.47E-07 | 3.88E-05 | pentatricopeptide |
| 166 | c45376_g1 | 609 | 599 | 1 | 0 | 0 | 22.693 | 24.175 | 0.042 | 0 | 0 | -10.5026 | 5.51E-18 | 2.24E-15 | pentatricopeptide |
| 167 | c41298_g1 | 733 | 655 | 0 | 0 | 1 | 75.71 | 73.683 | 0 | 0 | 0.073 | -10.7488 | 2.58E-19 | 1.30E-16 | peroxisomal membrane protein |
| 168 | c49821_g6 | 23.1 | 42.16 | 0 | 0 | 0 | 4.356 | 8.724 | 0 | 0 | 0 | -8.14997 | 0.000187 | 0.005336 | photosystem II reaction center protein |
| 169 | c27478_g1 | 13 | 36 | 0 | 0 | 0 | 9.567 | 29.898 | 0 | 0 | 0 | -7.74927 | 0.000654 | 0.015322 | LRR receptor serine/threonine kinase |
| 170 | c50822_g1 | 98 | 357 | 1 | 0 | 1 | 19.974 | 98.044 | 0.473 | 0 | 0.315 | -8.35324 | 2.39E-09 | 2.40E-07 | LRR receptor serine/threonine kinase |
| 171 | c15657_g1 | 344 | 158 | 0 | 0 | 1 | 27.505 | 13.816 | 0 | 0 | 0.057 | -9.28178 | 6.65E-11 | 8.95E-09 | plastid-lipid-associated protein 11 |
| 172 | c52369_g3 | 15.41 | 161 | 0 | 0 | 0 | 0.982 | 11.942 | 0 | 0 | 0 | -9.60057 | 5.23E-06 | 0.000245 | polyol transporter 6 |
| 173 | c35209_g2 | 231 | 503 | 0 | 0 | 0 | 46.781 | 96.482 | 0 | 0 | 0 | -11.6453 | 3.20E-14 | 7.46E-12 | protein Pop3-like |
| 174 | c32768_g1 | 334 | 16 | 0 | 0 | 1 | 21.426 | 1.118 | 0 | 0 | 0.049 | -8.75971 | 9.04E-07 | 5.22E-05 | S-adenosylmethionine ethyltransferase |
| 175 | c25104_g1 | 30 | 77 | 0 | 0 | 0 | 3.616 | 10.142 | 0 | 0 | 0 | -8.87168 | 1.85E-05 | 0.000739 | WRKY transcription factor 72 |
| 176 | c11096_g1 | 89 | 25 | 0 | 0 | 0 | 95.385 | 31.17 | 0 | 0 | 0 | -8.93187 | 1.84E-05 | 0.000735 | ZINC INDUCED FACILITATOR |
| 177 | c22638_g1 | 120 | 66 | 1 | 1 | 0 | 12.998 | 7.793 | 0.116 | 0.096 | 0 | -6.99505 | 3.02E-06 | 0.000153 | psbQ-like protein 2 |
| 178 | c33966_g1 | 253 | 111 | 0 | 0 | 1 | 29.512 | 14.116 | 0 | 0 | 0.089 | -8.82101 | 3.45E-09 | 3.37E-07 | psbQ-like protein 3 |
| 179 | c34583_g1 | 491 | 99 | 0 | 0 | 0 | 29.043 | 6.354 | 0 | 0 | 0 | -11.2977 | 2.43E-11 | 3.55E-09 | auxin efflux carrier component 8 |
| 180 | c60273_g1 | 15 | 15 | 0 | 0 | 0 | 2.548 | 2.784 | 0 | 0 | 0 | -7.0315 | 0.002468 | 0.04511 | phospholipid-transporting |
| 181 | c1110_g1 | 11 | 14 | 0 | 0 | 0 | 1.267 | 1.759 | 0 | 0 | 0 | -6.77419 | 0.003749 | 0.062547 | phospholipid-transporting |
| 182 | c20358_g1 | 19 | 11 | 0 | 0 | 0 | 3.203 | 2.028 | 0 | 0 | 0 | -7.02354 | 0.002599 | 0.046921 | zinc finger protein |
| 183 | c31967_g1 | 27 | 109 | 0 | 1 | 0 | 1.737 | 7.637 | 0 | 0.056 | 0 | -7.38891 | 2.85E-05 | 0.001076 | receptor-like protein kinase |
| 184 | c15289_g1 | 19 | 12 | 0 | 0 | 0 | 8.086 | 5.681 | 0 | 0 | 0 | -7.07171 | 0.002323 | 0.042987 | ribonuclease 3-like protein 3-like |
| 185 | c38181_g1 | 38 | 239 | 0 | 0 | 0 | 1.594 | 10.835 | 0 | 0 | 0 | -10.2511 | 8.88E-08 | 6.53E-06 | rop guanine nucleotide exchange factor |
| 186 | c47032_g1 | 14 | 14 | 1 | 0 | 0 | 3.573 | 3.995 | 0.21 | 0 | 0 | -5.04115 | 0.009257 | 0.122738 | ruvB-like 1-like |
| 187 | c34654_g2 | 329 | 139 | 0 | 0 | 0 | 38.638 | 17.8 | 0 | 0 | 0 | -10.9719 | 3.67E-11 | 5.16E-09 | S-norcoclaurine synthase-like |
| 188 | c29763_g2 | 24 | 27 | 1 | 1 | 0 | 3.132 | 3.85 | 0.147 | 0.12 | 0 | -5.13311 | 0.002537 | 0.04606 | transcription factor bHLH87-like |
| 189 | c30387_g1 | 25 | 19 | 0 | 0 | 0 | 2.961 | 2.235 | 0 | 0 | 0 | -7.57641 | 0.00078 | 0.017732 | transcription factor bHLH87-like |
| 190 | c37348_g1 | 401 | 119 | 0 | 0 | 0 | 21.469 | 7.782 | 0 | 0 | 0 | -11.1195 | 2.64E-11 | 3.81E-09 | transcription factor TCP5-like |
| 191 | c44073_g2 | 595 | 46 | 1 | 0 | 0 | 230.02 | 19.766 | 0.421 | 0 | 0 | -9.54282 | 2.32E-09 | 2.34E-07 | UDP-glycosyltransferase 85A3-like |
| 192 | c42671_g1 | 114 | 62 | 0 | 0 | 0 | 6.99 | 4.15 | 0 | 0 | 0 | -9.56567 | 5.09E-07 | 3.13E-05 | uncharacterized protein |
| 193 | c38691_g1 | 1663 | 413 | 0 | 0 | 0 | 61.815 | 16.63 | 0 | 0 | 0 | -13.1142 | 5.29E-20 | 2.89E-17 | uncharacterized protein |
| 194 | c34834_g1 | 333 | 117 | 1 | 1 | 1 | 30.324 | 13.412 | 0.158 | 0.136 | 0.089 | -7.7967 | 1.75E-09 | 1.81E-07 | uncharacterized protein |
| 195 | c73250_g1 | 274 | 528 | 0 | 0 | 0 | 29.384 | 61.699 | 0 | 0 | 0 | -11.7714 | 4.40E-15 | 1.21E-12 | uncharacterized protein |
| 196 | c18317_g1 | 126 | 134 | 0 | 0 | 0 | 9.909 | 11.456 | 0 | 0 | 0 | -10.1382 | 1.14E-08 | 1.01E-06 | uncharacterized protein |
| 197 | c9795_g1 | 607 | 291 | 0 | 0 | 0 | 58.697 | 30.632 | 0 | 0 | 0 | -11.9134 | 9.25E-16 | 2.81E-13 | uncharacterized protein |
| 198 | c28770_g1 | 348 | 313 | 1 | 0 | 0 | 45.685 | 44.852 | 0.147 | 0 | 0 | -9.62598 | 3.14E-13 | 6.28E-11 | uncharacterized protein |
| 199 | c28846_g1 | 636 | 316 | 0 | 0 | 0 | 73.489 | 39.688 | 0 | 0 | 0 | -11.9981 | 3.03E-16 | 9.81E-14 | uncharacterized protein |
| 200 | c35531_g1 | 2204 | 1699 | 1 | 1 | 1 | 281.585 | 236.78 | 0.137 | 0.12 | 0.097 | -10.9275 | 3.58E-29 | 4.79E-26 | uncharacterized protein |
| 201 | c45224_g3 | 17 | 242 | 0 | 0 | 0 | 1.367 | 21.205 | 0 | 0 | 0 | -10.1586 | 7.53E-07 | 4.43E-05 | uncharacterized protein |
| 202 | c45897_g1 | 206 | 719 | 0 | 1 | 0 | 11.048 | 39.936 | 0 | 0.048 | 0 | -10.1551 | 7.62E-14 | 1.68E-11 | uncharacterized protein |
| 203 | c54022_g1 | 12 | 103 | 0 | 1 | 0 | 1.666 | 20.097 | 0 | 0.207 | 0 | -7.15228 | 0.000147 | 0.00434 | uncharacterized protein |
| 204 | c38605_g1 | 572 | 374 | 0 | 0 | 0 | 99.542 | 65.332 | 0 | 0 | 0 | -11.993 | 1.18E-16 | 4.02E-14 | uncharacterized protein |
| 205 | c3136_g1 | 35 | 20 | 0 | 0 | 0 | 7.403 | 4.647 | 0 | 0 | 0 | -7.89263 | 0.000355 | 0.009233 | uncharacterized protein |
| 206 | c53394_g2 | 33 | 34 | 0 | 0 | 0 | 5.139 | 5.723 | 0 | 0 | 0 | -8.18507 | 0.000145 | 0.004291 | uncharacterized protein |
| 207 | c6642_g1 | 44 | 42 | 1 | 1 | 1 | 6.449 | 6.727 | 0.158 | 0.136 | 0.105 | -5.42393 | 0.000383 | 0.009851 | uncharacterized protein |
| 208 | c54188_g1 | 44 | 30 | 0 | 0 | 1 | 19.96 | 15.182 | 0 | 0 | 0.323 | -6.54904 | 0.000347 | 0.009053 | uncharacterized protein |
| 209 | c45119_g1 | 1561 | 530 | 1 | 1 | 1 | 77.447 | 27.931 | 0.074 | 0.072 | 0.032 | -10.0143 | 6.24E-20 | 3.37E-17 | uncharacterized protein |
| 210 | c39799_g1 | 1978 | 1145 | 1 | 1 | 1 | 145.256 | 90.634 | 0.105 | 0.08 | 0.057 | -10.6012 | 4.29E-26 | 4.47E-23 | uncharacterized protein |
| 211 | c40806_g1 | 26 | 18 | 0 | 0 | 0 | 5.453 | 3.974 | 0 | 0 | 0 | -7.57503 | 0.000792 | 0.017933 | uncharacterized protein |
| 212 | c28987_g1 | 124 | 12 | 0 | 1 | 0 | 33.242 | 3.55 | 0 | 0.247 | 0 | -7.34371 | 8.48E-05 | 0.002732 | uncharacterized protein |
| 213 | c47423_g4 | 20 | 62 | 0 | 0 | 0 | 2.62 | 8.869 | 0 | 0 | 0 | -8.49088 | 8.32E-05 | 0.002685 | uncharacterized protein |
| 214 | c49465_g5 | 405 | 184 | 1 | 0 | 0 | 37.656 | 17.955 | 0.095 | 0 | 0 | -9.44641 | 8.19E-12 | 1.29E-09 | very-long-chain enoyl-CoA reductase |
| 215 | c45624_g1 | 86 | 556 | 0 | 0 | 0 | 2.577 | 18.711 | 0 | 0 | 0 | -11.4634 | 2.53E-11 | 3.69E-09 | wall-associated receptor kinase 2-like |
| 216 | c29120_g1 | 327 | 2067 | 0 | 0 | 0 | 14.365 | 99.783 | 0 | 0 | 0 | -13.3617 | 8.52E-19 | 3.94E-16 | zinc finger protein CONSTANS |
| 217 | c48023_g2 | 230 | 21 | 0 | 1 | 0 | 7.987 | 0.787 | 0 | 0.032 | 0 | -8.22724 | 2.65E-06 | 0.000136 | inorganic phosphate transporter 1-4 |
| 218 | c47566_g2 | 112 | 117 | 0 | 1 | 0 | 25.74 | 33.168 | 0 | 0.2 | 0 | -8.12339 | 1.99E-07 | 1.34E-05 | leucine-rich repeat receptor-like  protein kinase |
| 219 | c49349_g3 | 35 | 334 | 0 | 1 | 0 | 6.99 | 38.86 | 0 | 0.231 | 0 | -8.83507 | 1.03E-07 | 7.45E-06 | WRKY transcription factor 40 |
| 220 | c35613_g1 | 196 | 12 | 0 | 0 | 0 | 12.585 | 0.838 | 0 | 0 | 0 | -9.78767 | 3.89E-06 | 0.000189 | Protein FDD123 |
| 221 | c38555_g1 | 16 | 63 | 0 | 0 | 0 | 1.21 | 4.419 | 0 | 0 | 0 | -8.43978 | 0.000117 | 0.003582 | 22 kDa kafirin cluster |
| 222 | c4510_g1 | 41 | 23 | 0 | 0 | 0 | 2.563 | 1.563 | 0 | 0 | 0 | -8.11012 | 0.0002 | 0.005676 | aldehyde dehydrogenase-like protein |
| 223 | c43541_g1 | 930 | 980 | 0 | 0 | 0 | 71.581 | 57.353 | 0 | 0 | 0 | -13.0139 | 2.19E-23 | 1.69E-20 | carboxyl-terminal proteinase |
| 224 | c43541_g2 | 721 | 521 | 0 | 0 | 1 | 37.456 | 29.432 | 0 | 0 | 0.04 | -10.5861 | 3.83E-18 | 1.59E-15 | carboxyl-terminal proteinase |
| 225 | c24852_g1 | 35 | 12 | 0 | 0 | 0 | 5.524 | 5.185 | 0 | 0 | 0 | -7.66028 | 0.000796 | 0.018018 | germin-like protein 2-1 |
| 226 | c41375_g2 | 129 | 21 | 0 | 0 | 1 | 20.857 | 3.829 | 0 | 0 | 0.089 | -7.5469 | 2.63E-05 | 0.001003 | multifunctional pol protein |
| 227 | c60734_g1 | 55 | 64 | 0 | 0 | 0 | 37.385 | 49.012 | 0 | 0 | 0 | -9.01352 | 6.87E-06 | 0.000312 | MYB transcription factor |
| 228 | c36207_g1 | 2099 | 1700 | 0 | 0 | 1 | 169.002 | 148.805 | 0 | 0 | 0.057 | -12.1948 | 9.66E-30 | 1.45E-26 | NADH dehydrogenase |
| 229 | c33182_g1 | 104 | 77 | 0 | 0 | 0 | 59.865 | 43.868 | 0 | 0 | 0 | -9.6106 | 3.28E-07 | 2.10E-05 | phospholipid-transporting ATPase 5 |
| 230 | c12860_g1 | 348 | 319 | 0 | 1 | 1 | 556.792 | 584.405 | 0 | 1.484 | 1.124 | -8.90923 | 6.31E-13 | 1.18E-10 | photosystem II oxygen evolving system |
| 231 | c53508_g3 | 29 | 240 | 0 | 0 | 1 | 0.498 | 4.46 | 0 | 0 | 0.016 | -8.43102 | 8.43E-07 | 4.92E-05 | polyprotein |
| 232 | c50295_g2 | 56 | 33 | 0 | 0 | 0 | 14.664 | 9.531 | 0 | 0 | 0 | -8.58505 | 4.01E-05 | 0.001442 | Putative protein |
| 233 | c26881_g1 | 153 | 340 | 0 | 1 | 0 | 40.489 | 40.774 | 0 | 0.04 | 0 | -9.24131 | 9.52E-11 | 1.25E-08 | reverse transcriptase |
| 234 | c41375_g1 | 236 | 113 | 1 | 0 | 0 | 15.148 | 7.689 | 0.105 | 0 | 0 | -8.68633 | 5.39E-09 | 5.07E-07 | transport protein |
| 235 | c35416_g1 | 201 | 22 | 0 | 0 | 0 | 13.596 | 1.604 | 0 | 0 | 0 | -9.89064 | 1.01E-06 | 5.76E-05 | transposable element |
| 236 | c55647_g1 | 18 | 20 | 0 | 0 | 0 | 1.224 | 1.48 | 0 | 0 | 0 | -7.37181 | 0.001221 | 0.025659 | uncharacterized protein |
| 237 | c47566_g1 | 43 | 43 | 0 | 0 | 0 | 10.62 | 12.615 | 0 | 0 | 0 | -8.5437 | 4.25E-05 | 0.001518 | Receptor-like protein 12 |
| 238 | c42368_g1 | 410 | 18 | 0 | 0 | 0 | 66.328 | 3.187 | 0 | 0 | 0 | -10.8267 | 6.85E-08 | 5.17E-06 | Receptor-like protein kinase |
| 239 | c53707_g7 | 11 | 31 | 0 | 0 | 0 | 3.516 | 10.949 | 0 | 0 | 0 | -7.52814 | 0.001148 | 0.024364 | Receptor-like protein kinase ANXUR2 |
| 240 | c73128_g1 | 56 | 17 | 0 | 1 | 0 | 18.75 | 6.313 | 0 | 0.311 | 0 | -6.45735 | 0.000555 | 0.0134 | Anthocyanidin 3-O-glucosyltransferase |
| 241 | c18385_g2 | 110 | 210 | 1 | 0 | 1 | 25.398 | 43.216 | 0.547 | 0 | 0.137 | -7.8376 | 2.43E-08 | 2.01E-06 | Geraniol 8-hydroxylase |
| 242 | c22867_g1 | 6159 | 3848 | 1 | 0 | 1 | 3819.629 | 2682.421 | 0.673 | 0 | 0.445 | -12.7893 | 5.47E-37 | 2.01E-33 | RuBisCO small subunit |
| 243 | c41511_g1 | 27 | 79 | 0 | 0 | 0 | 2.491 | 7.896 | 0 | 0 | 0 | -8.85973 | 2.15E-05 | 0.000841 | retrotransposon protein |
| 244 | c49616_g1 | 33 | 215 | 0 | 0 | 0 | 4.356 | 31.895 | 0 | 0 | 0 | -10.0919 | 2.35E-07 | 1.56E-05 | ribosomal protein S13 |
| 245 | c36168_g1 | 1077 | 756 | 0 | 0 | 0 | 122.192 | 92.508 | 0 | 0 | 0 | -12.9482 | 1.21E-22 | 8.88E-20 | RuBisCO small subunit |
| 246 | c13694_g1 | 760 | 476 | 0 | 0 | 0 | 1525.819 | 1097.435 | 0 | 0 | 0 | -12.3781 | 8.52E-19 | 3.94E-16 | RuBisCO small subunit |
| 247 | c52611_g1 | 45 | 37 | 0 | 0 | 0 | 6.891 | 4.636 | 0 | 0 | 0 | -8.47217 | 5.50E-05 | 0.001887 | RNA helicase family protein |
| 248 | c46941_g5 | 40 | 170 | 0 | 0 | 0 | 4.655 | 24.268 | 0 | 0 | 0 | -9.8486 | 3.79E-07 | 2.39E-05 | RNA polymerase beta subunit |
| 249 | c46720_g4 | 46 | 69 | 0 | 0 | 0 | 42.411 | 72.131 | 0 | 0 | 0 | -8.96813 | 9.12E-06 | 0.0004 | sesquiterpene cyclase |
| 250 | c31803_g1 | 68 | 30 | 0 | 0 | 0 | 14.521 | 7.358 | 0 | 0 | 0 | -8.71964 | 2.92E-05 | 0.001103 | stress response RCI peptide |
| 251 | c13660_g1 | 53 | 27 | 0 | 0 | 0 | 14.564 | 8.186 | 0 | 0 | 0 | -8.42962 | 7.48E-05 | 0.00246 | succinate dehydrogenase |
| 252 | c15867_g1 | 750 | 259 | 0 | 0 | 1 | 349.422 | 134.731 | 0 | 0 | 0.332 | -10.2799 | 5.98E-15 | 1.62E-12 | Thiamine thiazole synthase |
| 253 | c23216_g1 | 2016 | 892 | 1 | 1 | 1 | 958.776 | 473.901 | 0.515 | 0.439 | 0.34 | -10.4942 | 3.13E-24 | 2.61E-21 | thiazole biosynthetic protein |
| 254 | c40707_g1 | 154 | 494 | 1 | 1 | 0 | 11.361 | 39.708 | 0.084 | 0.072 | 0 | -8.83052 | 1.76E-11 | 2.63E-09 | Thioredoxin-like 4 |
| 255 | c46253_g1 | 71 | 176 | 0 | 0 | 0 | 5.424 | 14.478 | 0 | 0 | 0 | -10.0765 | 4.13E-08 | 3.25E-06 | TIR-NBS type disease  resistance protein |
| 256 | c13576_g1 | 26 | 20 | 1 | 0 | 0 | 1.566 | 1.304 | 0.063 | 0 | 0 | -5.75177 | 0.00229 | 0.042542 | TPA: hypothetical protein |
| 257 | c25686_g1 | 778 | 1347 | 0 | 1 | 0 | 36.702 | 69.927 | 0 | 0.056 | 0 | -11.3475 | 1.87E-22 | 1.33E-19 | transcription factor |
| 258 | c59747_g1 | 68 | 18 | 0 | 0 | 0 | 6.99 | 2.018 | 0 | 0 | 0 | -8.52565 | 8.07E-05 | 0.002618 | Transcriptional regulator |
| 259 | c72102_g1 | 26 | 11 | 0 | 0 | 0 | 2.022 | 0.931 | 0 | 0 | 0 | -7.31973 | 0.00159 | 0.031788 | Transposon Ty3-G Gag-Pol |
| 260 | c28975_g1 | 52 | 83 | 0 | 0 | 1 | 1.566 | 2.711 | 0 | 0 | 0.024 | -7.42123 | 1.33E-05 | 0.000556 | Ty3/gypsy retrotransposon protein |
| 261 | c50000_g4 | 79 | 91 | 0 | 0 | 0 | 39.777 | 51.247 | 0 | 0 | 0 | -9.52711 | 5.19E-07 | 3.18E-05 | tyrosine aminotransferase 1 |
| 262 | c23359_g1 | 44 | 24 | 0 | 0 | 0 | 4.869 | 2.898 | 0 | 0 | 0 | -8.19687 | 0.000158 | 0.004621 | ubiquitin ligase protein cop1 |
| 263 | c14710_g1 | 104 | 69 | 0 | 1 | 1 | 20.316 | 14.788 | 0 | 0.184 | 0.146 | -6.96763 | 4.45E-06 | 0.000213 | UDP-glucose glucosyltransferase |
| 264 | c11908_g1 | 40 | 30 | 1 | 0 | 0 | 13.397 | 11.135 | 0.368 | 0 | 0 | -6.35882 | 0.00046 | 0.011481 | UDP-glucose glucosyltransferase |
| 265 | c23107_g1 | 244 | 16 | 1 | 0 | 0 | 38.723 | 2.773 | 0.179 | 0 | 0 | -8.22978 | 3.90E-06 | 0.000189 | Uncharacterized protein |
| 266 | c28419_g1 | 11 | 12 | 0 | 0 | 0 | 1.466 | 1.739 | 0 | 0 | 0 | -6.65279 | 0.004467 | 0.071865 | Uncharacterized protein |
| 267 | c39617_g3 | 17 | 28 | 0 | 0 | 0 | 2.577 | 4.626 | 0 | 0 | 0 | -7.62018 | 0.000751 | 0.017201 | Uncharacterized protein |
| 268 | c7157_g1 | 98 | 38 | 0 | 0 | 0 | 7.431 | 3.125 | 0 | 0 | 0 | -9.18972 | 4.62E-06 | 0.00022 | Uncharacterized protein |
| 269 | c30469_g1 | 200 | 26 | 0 | 0 | 0 | 11.275 | 1.573 | 0 | 0 | 0 | -9.91095 | 6.85E-07 | 4.08E-05 | Uncharacterized protein |
| 270 | c33100_g1 | 16 | 21 | 0 | 1 | 0 | 22.935 | 34.42 | 0 | 1.333 | 0 | -5.50146 | 0.004284 | 0.069533 | Uncharacterized protein |
| 271 | c43817_g1 | 35 | 29 | 0 | 0 | 0 | 6.463 | 7.151 | 0 | 0 | 0 | -8.11589 | 0.000183 | 0.005249 | Uncharacterized protein |
| 272 | c39617_g2 | 25 | 21 | 0 | 0 | 0 | 3.645 | 3.374 | 0 | 0 | 0 | -7.64171 | 0.000657 | 0.015367 | Uncharacterized protein |
| 273 | c67739_g1 | 14 | 19 | 0 | 0 | 0 | 1.324 | 1.966 | 0 | 0 | 0 | -7.17253 | 0.001938 | 0.037233 | Uncharacterized protein |
| 274 | c34354_g1 | 53 | 118 | 0 | 0 | 0 | 61.573 | 156.142 | 0 | 0 | 0 | -9.54515 | 7.51E-07 | 4.42E-05 | Uncharacterized protein |
| 275 | c39832_g1 | 113 | 30 | 0 | 0 | 0 | 8.186 | 2.36 | 0 | 0 | 0 | -9.25761 | 5.12E-06 | 0.000241 | Uncharacterized protein |
| 276 | c31709_g1 | 36 | 15 | 0 | 0 | 0 | 3.488 | 1.583 | 0 | 0 | 0 | -7.77991 | 0.000559 | 0.013492 | Uncharacterized protein |
| 277 | c33495_g1 | 23 | 23 | 0 | 0 | 0 | 5.979 | 6.592 | 0 | 0 | 0 | -7.64434 | 0.000653 | 0.015313 | Uncharacterized protein |
| 278 | c37466_g1 | 32 | 46 | 1 | 0 | 0 | 4.684 | 6.064 | 0.105 | 0 | 0 | -6.52555 | 0.000282 | 0.007604 | Uncharacterized protein |
| 279 | c35403_g1 | 393 | 139 | 0 | 0 | 0 | 30.367 | 11.673 | 0 | 0 | 0 | -11.1544 | 1.08E-11 | 1.67E-09 | Uncharacterized protein |
| 280 | c15715_g1 | 66 | 42 | 0 | 0 | 1 | 11.546 | 8.051 | 0 | 0 | 0.129 | -7.08819 | 5.21E-05 | 0.001801 | Uncharacterized protein |
| 281 | c35103_g1 | 181 | 51 | 0 | 0 | 0 | 29.641 | 9.138 | 0 | 0 | 0 | -9.95538 | 1.33E-07 | 9.32E-06 | Uncharacterized protein |
| 282 | c29016_g1 | 123 | 27 | 0 | 0 | 0 | 19.945 | 4.791 | 0 | 0 | 0 | -9.32456 | 4.66E-06 | 0.000221 | Uncharacterized protein |
| 283 | c40852_g1 | 137 | 17 | 0 | 0 | 1 | 18.237 | 2.473 | 0 | 0 | 0.097 | -7.58399 | 3.17E-05 | 0.00118 | Uncharacterized protein |
| 284 | c49523_g1 | 97 | 496 | 0 | 0 | 0 | 28.331 | 121.877 | 0 | 0 | 0 | -11.347 | 2.60E-11 | 3.77E-09 | Uncharacterized protein |
| 285 | c50271_g4 | 33 | 103 | 0 | 0 | 0 | 15.731 | 55.987 | 0 | 0 | 0 | -9.21938 | 5.36E-06 | 0.000251 | Uncharacterized protein |
| 286 | c24123_g1 | 103 | 55 | 0 | 0 | 0 | 36.488 | 21.619 | 0 | 0 | 0 | -9.40998 | 1.23E-06 | 6.88E-05 | Uncharacterized protein |
| 287 | c43566_g1 | 11 | 81 | 0 | 1 | 0 | 1.594 | 12.77 | 0 | 0.136 | 0 | -6.82984 | 0.000342 | 0.008951 | uncharacterized protein |
| 288 | c46178_g1 | 106 | 48 | 0 | 0 | 0 | 112.924 | 58.15 | 0 | 0 | 0 | -9.37076 | 1.67E-06 | 9.01E-05 | uncharacterized protein |
| 289 | c25118_g1 | 60 | 57 | 0 | 0 | 0 | 92.452 | 100.518 | 0 | 0 | 0 | -8.98598 | 7.69E-06 | 0.000345 | unknown |
| 290 | c43980_g1 | 76 | 28 | 0 | 0 | 1 | 10.378 | 5.164 | 0 | 0 | 0.162 | -7.02814 | 8.80E-05 | 0.002821 | unkown |
| 291 | c29851_g2 | 13.59 | 34.78 | 0 | 0 | 0 | 1.025 | 2.836 | 0 | 0 | 0 | -7.74803 | 0.000625 | 0.014763 | unkown |
| 292 | c51945_g3 | 31 | 16 | 1 | 0 | 1 | 4.242 | 2.391 | 0.147 | 0 | 0.097 | -5.05399 | 0.003779 | 0.062926 | unkown |
| 293 | c30794_g1 | 14 | 21 | 0 | 1 | 1 | 2.264 | 3.715 | 0 | 0.152 | 0.121 | -4.69001 | 0.008307 | 0.113504 | unkown |
| 294 | c52581_g1 | 28 | 25 | 0 | 0 | 0 | 3.118 | 3.032 | 0 | 0 | 0 | -7.84603 | 0.000378 | 0.009746 | unkown |
| 295 | c43009_g5 | 44.68 | 19 | 0 | 0 | 0 | 8.072 | 3.757 | 0 | 0 | 0 | -8.10626 | 0.000224 | 0.006256 | unkown |
| 296 | c31064_g1 | 28 | 15 | 0 | 0 | 0 | 5.723 | 3.363 | 0 | 0 | 0 | -7.53837 | 0.000903 | 0.020025 | unkown |
| 297 | c52647_g1 | 35 | 25 | 0 | 1 | 0 | 7.261 | 5.713 | 0 | 0.192 | 0 | -6.18687 | 0.000854 | 0.019095 | unkown |
| 298 | c47553_g6 | 104 | 299 | 0 | 0 | 0 | 19.661 | 63.283 | 0 | 0 | 0 | -10.7841 | 3.71E-10 | 4.36E-08 | unkown |
| 299 | c41174_g1 | 62 | 476 | 0 | 0 | 0 | 13.923 | 83.525 | 0 | 0 | 0 | -11.2096 | 3.49E-10 | 4.13E-08 | unkown |
| 300 | c44858_g3 | 60 | 119 | 0 | 0 | 0 | 6.25 | 13.495 | 0 | 0 | 0 | -9.60947 | 4.72E-07 | 2.91E-05 | unkown |
| 301 | c45775_g1 | 28 | 65 | 0 | 0 | 0 | 7.958 | 20.387 | 0 | 0 | 0 | -8.66853 | 3.68E-05 | 0.001345 | unkown |
| 302 | c9502_g1 | 17 | 14 | 1 | 1 | 1 | 3.445 | 3.115 | 0.221 | 0.184 | 0.146 | -3.95547 | 0.015692 | 0.177761 | unkown |
| 303 | c44961_g9 | 25 | 36 | 0 | 1 | 1 | 5.125 | 8.341 | 0 | 0.184 | 0.186 | -5.48499 | 0.001326 | 0.027429 | unkown |
| 304 | c47494_g1 | 25 | 13 | 1 | 0 | 0 | 4.627 | 3.167 | 1.147 | 0 | 0 | -5.46961 | 0.004654 | 0.074067 | unkown |
| 305 | c45821_g3 | 117 | 121 | 1 | 0 | 1 | 7.303 | 6.654 | 0.042 | 0 | 0.032 | -7.40129 | 2.98E-07 | 1.93E-05 | unkown |
| 306 | c47921_g1 | 26 | 39 | 0 | 1 | 0 | 27.32 | 46.59 | 0 | 0.974 | 0 | -6.31323 | 0.000613 | 0.014571 | unkown |
| 307 | c32989_g1 | 19 | 23 | 0 | 1 | 0 | 3.645 | 8.051 | 0 | 0.128 | 0 | -5.68221 | 0.002949 | 0.051614 | unkown |
| 308 | c18925_g1 | 36 | 62 | 0 | 0 | 0 | 6.207 | 13.257 | 0 | 0 | 0 | -8.73984 | 2.37E-05 | 0.000916 | unkown |
| 309 | c41862_g1 | 99 | 88 | 0 | 0 | 0 | 19.504 | 19.031 | 0 | 0 | 0 | -9.66041 | 2.41E-07 | 1.59E-05 | unkown |
| 310 | c50807_g2 | 52 | 43 | 0 | 0 | 0 | 11.66 | 15.947 | 0 | 0 | 0 | -8.684 | 2.55E-05 | 0.000978 | unkown |
| 311 | c42716_g1 | 107 | 21 | 0 | 0 | 0 | 12.998 | 2.784 | 0 | 0 | 0 | -9.09515 | 1.42E-05 | 0.000588 | unkown |
| 312 | c45354_g1 | 14 | 15 | 0 | 0 | 0 | 3.217 | 4.14 | 0 | 0 | 0 | -6.984 | 0.002656 | 0.047753 | unkown |
| 313 | c41862_g3 | 124 | 202 | 0 | 0 | 0 | 23.077 | 41.219 | 0 | 0 | 0 | -10.4708 | 1.30E-09 | 1.38E-07 | unkown |
| 314 | c53297_g2 | 27 | 27 | 0 | 0 | 0 | 7.859 | 2.711 | 0 | 0 | 0 | -7.8746 | 0.000351 | 0.009133 | unkown |
| 315 | c53912_g3 | 29 | 34 | 0 | 0 | 1 | 29.256 | 38.963 | 0 | 0 | 0.72 | -6.32712 | 0.000643 | 0.015115 | unkown |
| 316 | c46811_g2 | 115 | 763 | 0 | 0 | 0 | 8.528 | 59.236 | 0 | 0 | 0 | -11.9151 | 7.58E-13 | 1.39E-10 | unkown |
| 317 | c40773_g1 | 44 | 48 | 0 | 0 | 0 | 4.072 | 4.833 | 0 | 0 | 0 | -8.64208 | 2.99E-05 | 0.001125 | unkown |
| 318 | c45264_g4 | 60 | 19 | 1 | 0 | 0 | 7.83 | 2.525 | 0.2 | 0 | 0 | -6.52011 | 0.000386 | 0.009919 | unkown |
| 319 | c57930_g1 | 49 | 425 | 0 | 0 | 1 | 36.816 | 360.468 | 0 | 0 | 0.534 | -9.24293 | 9.30E-09 | 8.40E-07 | unkown |
| 320 | c62705_g1 | 23 | 11 | 0 | 0 | 0 | 1.566 | 0.818 | 0 | 0 | 0 | -7.20017 | 0.001926 | 0.037088 | unkown |
| 321 | c46139_g6 | 26 | 47 | 0 | 0 | 0 | 3.716 | 7.689 | 0 | 0 | 0 | -8.31675 | 0.00011 | 0.003401 | unkown |
| 322 | c30065_g1 | 18 | 13 | 0 | 0 | 0 | 11.503 | 12.657 | 0 | 0 | 0 | -7.07364 | 0.00228 | 0.042419 | unkown |
| 323 | c54573_g1 | 21 | 29 | 0 | 0 | 0 | 4.442 | 6.737 | 0 | 0 | 0 | -7.76892 | 0.000485 | 0.012003 | unkown |
| 324 | c18446_g1 | 15 | 17 | 0 | 0 | 0 | 6.549 | 8.269 | 0 | 0 | 0 | -7.12579 | 0.002068 | 0.039281 | unkown |
| 325 | c45506_g1 | 35 | 36 | 0 | 0 | 0 | 9.738 | 11.052 | 0 | 0 | 0 | -8.26843 | 0.000112 | 0.003466 | unkown |
| 326 | c24171_g1 | 66 | 30 | 0 | 0 | 0 | 10.863 | 5.402 | 0 | 0 | 0 | -8.69037 | 3.15E-05 | 0.001176 | unkown |
| 327 | c37973_g1 | 67 | 107 | 0 | 0 | 0 | 10.435 | 18.224 | 0 | 0 | 0 | -9.56556 | 4.89E-07 | 3.01E-05 | unkown |
| 328 | c47885_g3 | 20 | 36 | 0 | 0 | 0 | 3.616 | 7.141 | 0 | 0 | 0 | -7.93555 | 0.00034 | 0.00892 | unkown |
| 329 | c45565_g1 | 775 | 1102 | 0 | 0 | 0 | 61.644 | 91.297 | 0 | 0 | 0 | -12.9934 | 7.38E-23 | 5.53E-20 | unkown |
| 330 | c44926_g5 | 14 | 31 | 0 | 0 | 0 | 3.872 | 9.479 | 0 | 0 | 0 | -7.62421 | 0.000784 | 0.017791 | unkown |
| 331 | c38674_g1 | 23 | 27 | 1 | 1 | 0 | 8.67 | 10.194 | 0.368 | 0.311 | 0 | -5.10515 | 0.002725 | 0.048667 | unkown |
| 332 | c4078_g1 | 25 | 18 | 0 | 0 | 1 | 13.069 | 10.525 | 0 | 0 | 0.372 | -5.77433 | 0.002693 | 0.048211 | unkown |
| 333 | c16495_g1 | 56 | 54 | 0 | 0 | 0 | 8.129 | 8.569 | 0 | 0 | 0 | -8.89739 | 1.10E-05 | 0.000471 | unkown |
| 334 | c28249_g1 | 35 | 51 | 0 | 0 | 0 | 7.944 | 13.06 | 0 | 0 | 0 | -8.54942 | 4.39E-05 | 0.001557 | unkown |
| 335 | c49476_g6 | 17 | 31 | 0 | 0 | 0 | 5.609 | 10.494 | 0 | 0 | 0 | -7.71428 | 0.000593 | 0.014163 | unkown |
| 336 | c52825_g3 | 67 | 19 | 0 | 0 | 0 | 6.52 | 2.018 | 0 | 0 | 0 | -8.52638 | 7.65E-05 | 0.002511 | unkown |
| 337 | c28066_g1 | 129 | 74 | 0 | 0 | 0 | 22.323 | 14.033 | 0 | 0 | 0 | -9.77208 | 1.47E-07 | 1.03E-05 | unkown |
| 338 | c41129_g1 | 15 | 27 | 0 | 0 | 0 | 9.809 | 9.924 | 0 | 0 | 0 | -7.5224 | 0.000977 | 0.021405 | unkown |
| 339 | c10405_g1 | 46 | 42 | 1 | 0 | 0 | 2.918 | 2.887 | 0.074 | 0 | 0 | -6.69398 | 0.000153 | 0.00451 | unkown |
| 340 | c53654_g1 | 28 | 55 | 0 | 0 | 0 | 5.837 | 8.859 | 0 | 0 | 0 | -8.5026 | 5.94E-05 | 0.002014 | unkown |
| 341 | c38813_g1 | 771 | 175 | 0 | 1 | 0 | 94.887 | 23.492 | 0 | 0.112 | 0 | -10.15 | 1.81E-13 | 3.74E-11 | unkown |
| 342 | c50154_g10 | 19 | 14 | 0 | 0 | 0 | 4.171 | 2.608 | 0 | 0 | 0 | -7.16347 | 0.001938 | 0.037233 | unkown |
| 343 | c32389_g1 | 154 | 217 | 0 | 0 | 0 | 27.59 | 42.596 | 0 | 0 | 0 | -10.6551 | 2.32E-10 | 2.85E-08 | unkown |
| 344 | c47315_g3 | 30 | 16 | 0 | 0 | 0 | 3.673 | 1.863 | 0 | 0 | 0 | -7.63509 | 0.000715 | 0.016482 | unkown |
| 345 | c36111_g1 | 11 | 31 | 1 | 1 | 0 | 4.627 | 14.509 | 0.452 | 0.391 | 0 | -4.86421 | 0.006006 | 0.089981 | unkown |
| 346 | c44767_g2 | 32 | 11 | 0 | 1 | 0 | 6.051 | 2.287 | 0 | 0.176 | 0 | -5.69799 | 0.003362 | 0.057381 | unkown |
| 347 | c60160_g1 | 222 | 107 | 0 | 0 | 1 | 199.696 | 109.086 | 0 | 0 | 0.639 | -8.67738 | 9.10E-09 | 8.23E-07 | unkown |
| 348 | c53269_g2 | 22 | 99 | 0 | 0 | 0 | 41.357 | 147.874 | 0 | 0 | 0 | -9.0548 | 1.58E-05 | 0.000643 | unkown |
| 349 | c37658_g1 | 39 | 32 | 0 | 0 | 0 | 8.072 | 7.575 | 0 | 0 | 0 | -8.26497 | 0.000113 | 0.003494 | unkown |
| 350 | c54186_g3 | 71 | 177 | 0 | 0 | 0 | 5.709 | 14.323 | 0 | 0 | 0 | -10.0824 | 3.95E-08 | 3.13E-06 | unkown |
| 351 | c32485_g1 | 28 | 20 | 0 | 0 | 0 | 8.969 | 7.099 | 0 | 0 | 0 | -7.70038 | 0.00058 | 0.0139 | unkown |
| 352 | c58703_g1 | 88 | 103 | 0 | 0 | 0 | 18.251 | 23.471 | 0 | 0 | 0 | -9.69518 | 2.01E-07 | 1.35E-05 | unkown |
| 353 | c51119_g4 | 129 | 45 | 0 | 0 | 0 | 11.46 | 4.098 | 0 | 0 | 0 | -9.54332 | 9.11E-07 | 5.25E-05 | unkown |
| 354 | c48649_g2 | 145 | 55 | 0 | 0 | 0 | 26.651 | 14.975 | 0 | 0 | 0 | -9.74499 | 2.76E-07 | 1.80E-05 | unkown |
| 355 | c43702_g1 | 32 | 34 | 0 | 1 | 1 | 1.95 | 2.256 | 0 | 0.056 | 0.049 | -5.5932 | 0.000955 | 0.021007 | unkown |
| 356 | c60172_g1 | 19 | 28 | 0 | 0 | 0 | 5.097 | 8.279 | 0 | 0 | 0 | -7.68102 | 0.000604 | 0.014374 | unkown |
| 357 | c49471_g2 | 19 | 42 | 0 | 0 | 0 | 6.136 | 19.259 | 0 | 0 | 0 | -8.06124 | 0.000259 | 0.007076 | unkown |
| 358 | c53038_g5 | 11 | 107 | 0 | 0 | 0 | 1.438 | 14.675 | 0 | 0 | 0 | -9.02411 | 4.05E-05 | 0.001454 | unkown |
| 359 | c48451_g4 | 20 | 13 | 0 | 0 | 0 | 5.823 | 4.181 | 0 | 0 | 0 | -7.16165 | 0.00198 | 0.037899 | unkown |
| 360 | c15622_g1 | 58 | 22 | 0 | 1 | 0 | 4.869 | 2.008 | 0 | 0.08 | 0 | -6.59193 | 0.000317 | 0.008395 | unkown |
| 361 | c71666_g1 | 20 | 15 | 1 | 0 | 1 | 2.164 | 1.77 | 0.116 | 0 | 0.081 | -4.63635 | 0.008788 | 0.118355 | unkown |
| 362 | c47315_g5 | 247 | 283 | 0 | 1 | 0 | 37.94 | 52.903 | 0 | 0.144 | 0 | -9.33638 | 1.04E-11 | 1.62E-09 | unkown |
| 363 | c38750_g1 | 68 | 29 | 0 | 0 | 0 | 4.157 | 1.925 | 0 | 0 | 0 | -8.70444 | 3.13E-05 | 0.001169 | unkown |
| 364 | c33037_g1 | 25 | 15 | 0 | 0 | 0 | 13.98 | 11.021 | 0 | 0 | 0 | -7.43623 | 0.001102 | 0.023582 | unkown |
| 365 | c35066_g1 | 88 | 28 | 1 | 0 | 0 | 12.258 | 4.233 | 0.147 | 0 | 0 | -7.07749 | 6.13E-05 | 0.00207 | unkown |
| 366 | c53433_g4 | 24 | 35 | 0 | 0 | 0 | 1.509 | 2.401 | 0 | 0 | 0 | -8.00752 | 0.000254 | 0.006951 | unkown |
| 367 | c35883_g1 | 21 | 34 | 0 | 0 | 0 | 2.321 | 4.129 | 0 | 0 | 0 | -7.90815 | 0.000348 | 0.009088 | unkown |
| 368 | c9536_g1 | 25 | 26 | 0 | 0 | 0 | 3.445 | 3.912 | 0 | 0 | 0 | -7.7931 | 0.000444 | 0.011149 | unkown |
| 369 | c36186_g1 | 13 | 33 | 0 | 0 | 0 | 7.346 | 20.915 | 0 | 0 | 0 | -7.65749 | 0.000781 | 0.017744 | unkown |
| 370 | c50125_g1 | 326 | 355 | 0 | 0 | 0 | 32.246 | 37.928 | 0 | 0 | 0 | -11.5269 | 2.34E-14 | 5.68E-12 | unkown |
| 371 | c47589_g4 | 36 | 106 | 0 | 0 | 0 | 10.706 | 33.095 | 0 | 0 | 0 | -9.2809 | 3.82E-06 | 0.000187 | unkown |
| 372 | c19758_g1 | 361 | 465 | 1 | 1 | 1 | 127.204 | 181.797 | 0.379 | 0.327 | 0.251 | -8.69342 | 2.44E-14 | 5.85E-12 | unkown |
| 373 | c20058_g1 | 14 | 35 | 0 | 0 | 0 | 1.039 | 2.825 | 0 | 0 | 0 | -7.74803 | 0.000625 | 0.014763 | unkown |
| 374 | c50275_g3 | 19.19 | 69.06 | 0 | 0 | 0 | 1.167 | 4.781 | 0 | 0 | 0 | -8.59422 | 6.58E-05 | 0.002198 | unkown |
| 375 | c64490_g1 | 14 | 17 | 0 | 0 | 1 | 3.63 | 4.874 | 0 | 0 | 0.186 | -5.31455 | 0.006891 | 0.099301 | unkown |
| 376 | c36543_g1 | 27 | 18 | 1 | 0 | 0 | 11.831 | 8.931 | 0.368 | 0 | 0 | -5.71778 | 0.002488 | 0.045339 | unkown |
| 377 | c39571_g1 | 12 | 206 | 0 | 1 | 1 | 0.797 | 23.595 | 0 | 0.048 | 0.186 | -7.33943 | 1.98E-05 | 0.000783 | unkown |
| 378 | c48626_g1 | 12 | 43 | 0 | 0 | 0 | 3.531 | 13.971 | 0 | 0 | 0 | -7.91809 | 0.000477 | 0.011831 | unkown |
| 379 | c19152_g1 | 48 | 113 | 0 | 0 | 0 | 9.183 | 23.73 | 0 | 0 | 0 | -9.45906 | 1.25E-06 | 6.98E-05 | unkown |
| 380 | c54666_g1 | 33 | 25 | 0 | 0 | 0 | 3.645 | 3.001 | 0 | 0 | 0 | -7.97306 | 0.000276 | 0.00748 | unkown |
| 381 | c30504_g1 | 15 | 19 | 0 | 0 | 0 | 6.036 | 8.517 | 0 | 0 | 0 | -7.2143 | 0.00166 | 0.032903 | unkown |
| 382 | c39522_g1 | 29 | 79 | 0 | 1 | 0 | 10.435 | 25.541 | 0 | 0.255 | 0 | -7.05245 | 7.28E-05 | 0.002405 | unkown |
| 383 | c39195_g2 | 88 | 11 | 1 | 0 | 1 | 13.155 | 3.074 | 0.168 | 0 | 0.315 | -6.1106 | 0.000491 | 0.012109 | unkown |
| 384 | c48056_g3 | 119 | 58 | 0 | 0 | 0 | 10.535 | 5.661 | 0 | 0 | 0 | -9.57229 | 5.50E-07 | 3.35E-05 | unkown |
| 385 | c51377_g2 | 17 | 77 | 0 | 0 | 0 | 1.794 | 8.859 | 0 | 0 | 0 | -8.6913 | 5.87E-05 | 0.001993 | unkown |
| 386 | c5033_g1 | 34 | 15 | 0 | 0 | 0 | 15.219 | 7.493 | 0 | 0 | 0 | -7.72322 | 0.000614 | 0.01458 | unkown |
| 387 | c64125_g1 | 70 | 39 | 0 | 0 | 0 | 12.243 | 7.472 | 0 | 0 | 0 | -8.87598 | 1.37E-05 | 0.000569 | unkown |
| 388 | c27848_g1 | 15 | 16 | 0 | 0 | 0 | 3.502 | 4.108 | 0 | 0 | 0 | -7.07941 | 0.002249 | 0.041998 | unkown |
| 389 | c51435_g2 | 29 | 37 | 0 | 0 | 0 | 5.581 | 7.813 | 0 | 0 | 0 | -8.1667 | 0.000157 | 0.004595 | unkown |
| 390 | c4878_g1 | 25 | 11 | 1 | 1 | 0 | 8.67 | 4.233 | 0.379 | 0.319 | 0 | -4.61709 | 0.008975 | 0.120309 | unkown |
| 391 | c40050_g1 | 38 | 31 | 0 | 0 | 0 | 4.171 | 3.705 | 0 | 0 | 0 | -8.22379 | 0.00013 | 0.003918 | unkown |
| 392 | c27058_g1 | 69 | 30 | 1 | 0 | 0 | 12.158 | 6.23 | 0.189 | 0 | 0 | -6.85267 | 0.00011 | 0.003408 | unkown |
| 393 | c9760_g1 | 11 | 20 | 0 | 0 | 0 | 5.424 | 11.011 | 0 | 0 | 0 | -7.08709 | 0.002436 | 0.044628 | unkown |
| 394 | c38809_g1 | 469 | 45 | 0 | 0 | 0 | 61.146 | 6.934 | 0 | 0 | 0 | -11.0937 | 1.65E-09 | 1.72E-07 | unkown |
| 395 | c4733_g1 | 16 | 13 | 0 | 0 | 0 | 2.491 | 2.204 | 0 | 0 | 0 | -6.9799 | 0.002662 | 0.047803 | unkown |
| 396 | c27430_g1 | 27 | 11 | 1 | 0 | 1 | 4.684 | 2.09 | 0.189 | 0 | 0.129 | -4.74568 | 0.007979 | 0.110417 | unkown |
| 397 | c48593_g3 | 102 | 253 | 0 | 0 | 0 | 23.889 | 60.913 | 0 | 0 | 0 | -10.5994 | 1.01E-09 | 1.10E-07 | unkown |
| 398 | c20532_g1 | 17 | 13 | 0 | 0 | 0 | 10.734 | 9.231 | 0 | 0 | 0 | -7.02753 | 0.002492 | 0.045388 | unkown |
| 399 | c1192_g1 | 19 | 24 | 0 | 1 | 0 | 2.662 | 3.684 | 0 | 0.128 | 0 | -5.71664 | 0.002742 | 0.048925 | unkown |
| 400 | c31207_g1 | 19 | 11 | 0 | 0 | 0 | 2.292 | 1.449 | 0 | 0 | 0 | -7.02354 | 0.002599 | 0.046921 | unkown |
| 401 | c45877_g4 | 23 | 16 | 0 | 0 | 0 | 11.019 | 8.558 | 0 | 0 | 0 | -7.40206 | 0.001162 | 0.024587 | unkown |
| 402 | c42260_g2 | 69 | 18 | 0 | 0 | 0 | 8.158 | 2.318 | 0 | 0 | 0 | -8.54213 | 7.65E-05 | 0.00251 | unkown |
| 403 | c53487_g5 | 23 | 43 | 0 | 0 | 0 | 13.539 | 28.428 | 0 | 0 | 0 | -8.17225 | 0.000175 | 0.005056 | unkown |
| 404 | c18556_g1 | 30 | 12 | 1 | 0 | 1 | 4.698 | 2.049 | 0.168 | 0 | 0.113 | -4.88903 | 0.005751 | 0.087131 | unkown |
| 405 | c36968_g1 | 35 | 12 | 1 | 1 | 1 | 6.449 | 2.422 | 0.2 | 0.168 | 0.137 | -4.54021 | 0.005578 | 0.085 | unkown |
| 406 | c32082_g1 | 23 | 19 | 0 | 0 | 0 | 7.133 | 6.52 | 0 | 0 | 0 | -7.5109 | 0.000901 | 0.019992 | unkown |
| 407 | c28944_g1 | 25 | 26 | 0 | 0 | 0 | 4.954 | 5.516 | 0 | 0 | 0 | -7.7931 | 0.000444 | 0.011149 | unkown |
| 408 | c45735_g1 | 32 | 27 | 0 | 0 | 1 | 5.353 | 4.947 | 0 | 0 | 0.121 | -6.22838 | 0.000858 | 0.019168 | unkown |
| 409 | c41862_g2 | 93 | 93 | 0 | 0 | 0 | 45.173 | 50.481 | 0 | 0 | 0 | -9.65452 | 2.44E-07 | 1.61E-05 | unkown |
| 410 | c45786_g2 | 45 | 55 | 0 | 1 | 1 | 5.04 | 7.275 | 0 | 0.096 | 0.073 | -6.19091 | 0.000137 | 0.004114 | unkown |
| 411 | c54195_g3 | 109 | 49 | 0 | 0 | 0 | 14.251 | 6.996 | 0 | 0 | 0 | -9.4076 | 1.43E-06 | 7.85E-05 | unkown |
| 412 | c22991_g1 | 24 | 24 | 1 | 0 | 0 | 36.987 | 42.326 | 1.631 | 0 | 0 | -5.81741 | 0.001954 | 0.037509 | unkown |
| 413 | c62273_g1 | 36 | 14 | 0 | 0 | 0 | 5.467 | 2.328 | 0 | 0 | 0 | -7.75061 | 0.000607 | 0.014445 | unkown |
| 414 | c46527_g3 | 33 | 37 | 0 | 0 | 0 | 3.573 | 4.357 | 0 | 0 | 0 | -8.24935 | 0.000121 | 0.003672 | unkown |
| 415 | c43701_g1 | 85 | 49 | 0 | 0 | 0 | 9.795 | 6.075 | 0 | 0 | 0 | -9.17378 | 3.76E-06 | 0.000184 | unkown |
| 416 | c53836_g2 | 19 | 56 | 0 | 0 | 0 | 3.346 | 10.783 | 0 | 0 | 0 | -8.36192 | 0.000126 | 0.003805 | unkown |
| 417 | c41885_g3 | 19 | 13 | 0 | 0 | 0 | 11.475 | 8.827 | 0 | 0 | 0 | -7.11832 | 0.002104 | 0.039787 | unkown |
| 418 | c17004_g1 | 72 | 133 | 1 | 0 | 0 | 60.349 | 126.141 | 0.894 | 0 | 0 | -7.93297 | 7.29E-07 | 4.31E-05 | unkown |
| 419 | c27680_g1 | 11 | 76 | 0 | 0 | 0 | 2.207 | 16.734 | 0 | 0 | 0 | -8.58325 | 0.000117 | 0.003582 | unkown |
| 420 | c37932_g1 | 180 | 16 | 0 | 0 | 0 | 12.913 | 1.242 | 0 | 0 | 0 | -9.7036 | 3.18E-06 | 0.00016 | unkown |
| 421 | c42039_g1 | 132 | 14 | 0 | 0 | 0 | 25.099 | 3.187 | 0 | 0 | 0 | -9.28028 | 1.41E-05 | 0.000583 | unkown |
| 422 | c18878_g1 | 108 | 223 | 0 | 0 | 0 | 22.551 | 51.133 | 0 | 0 | 0 | -10.4961 | 1.55E-09 | 1.63E-07 | unkown |
| 423 | c35269_g1 | 52 | 37 | 0 | 0 | 0 | 11.347 | 8.879 | 0 | 0 | 0 | -8.58783 | 3.76E-05 | 0.001367 | unkown |
| 424 | c48694_g2 | 39 | 64 | 0 | 0 | 0 | 2.99 | 5.764 | 0 | 0 | 0 | -8.81078 | 1.76E-05 | 0.00071 | unkown |
| 425 | c50254_g2 | 13 | 188 | 0 | 0 | 0 | 1.908 | 32.154 | 0 | 0 | 0 | -9.79326 | 3.64E-06 | 0.000179 | unkown |
| 426 | c50317_g9 | 205 | 72 | 0 | 0 | 0 | 10.734 | 4.088 | 0 | 0 | 0 | -10.2134 | 1.81E-08 | 1.54E-06 | unkown |
| 427 | c52106_g3 | 170 | 56 | 0 | 0 | 0 | 61.844 | 20.708 | 0 | 0 | 0 | -9.91939 | 1.26E-07 | 8.87E-06 | unkown |
| 428 | c47665_g9 | 61 | 43 | 0 | 0 | 0 | 12.898 | 9.987 | 0 | 0 | 0 | -8.81185 | 1.61E-05 | 0.000654 | unkown |
| 429 | c50948_g6 | 43 | 31 | 0 | 0 | 1 | 5.182 | 5.05 | 0 | 0 | 0.081 | -6.54979 | 0.000342 | 0.008955 | unkown |
| 430 | c40669_g2 | 29 | 52 | 0 | 0 | 0 | 4.399 | 8.631 | 0 | 0 | 0 | -8.46623 | 6.60E-05 | 0.002203 | unkown |
| 431 | c54753_g1 | 24 | 20 | 0 | 0 | 0 | 3.844 | 3.508 | 0 | 0 | 0 | -7.57779 | 0.000772 | 0.017584 | unkown |
| 432 | c51707_g2 | 74 | 56 | 0 | 0 | 0 | 29.27 | 24.64 | 0 | 0 | 0 | -9.1342 | 4.05E-06 | 0.000196 | unkown |
| 433 | c46208_g4 | 11 | 41 | 0 | 0 | 0 | 5.666 | 23.616 | 0 | 0 | 0 | -7.83789 | 0.000622 | 0.014713 | unkown |
| 434 | c68025_g1 | 15 | 16 | 0 | 0 | 0 | 1.95 | 2.277 | 0 | 0 | 0 | -7.07941 | 0.002249 | 0.041998 | unkown |
| 435 | c31974_g1 | 135 | 21 | 0 | 0 | 0 | 57.06 | 9.883 | 0 | 0 | 0 | -9.37816 | 5.70E-06 | 0.000264 | unkown |
| 436 | c48041_g4 | 13 | 17 | 0 | 0 | 0 | 6.691 | 9.79 | 0 | 0 | 0 | -7.03547 | 0.002517 | 0.045762 | unkown |
| 437 | c34800_g1 | 20 | 24 | 0 | 0 | 0 | 3.716 | 4.885 | 0 | 0 | 0 | -7.58329 | 0.000772 | 0.017583 | unkown |
| 438 | c63618_g1 | 26 | 30 | 0 | 0 | 0 | 41.599 | 54.962 | 0 | 0 | 0 | -7.92903 | 0.000308 | 0.008217 | unkown |
| 439 | c21772_g1 | 88 | 22 | 1 | 1 | 0 | 13.852 | 3.85 | 0.179 | 0.144 | 0 | -6.22123 | 0.000171 | 0.004935 | unkown |
| 440 | c49090_g4 | 22 | 17 | 0 | 0 | 0 | 10.606 | 9.159 | 0 | 0 | 0 | -7.40361 | 0.00114 | 0.024232 | unkown |
| 441 | c43053_g1 | 93 | 323 | 1 | 1 | 1 | 11.46 | 42.637 | 0.137 | 0.71 | 0.089 | -7.71538 | 7.05E-09 | 6.51E-07 | unkown |
| 442 | c18676_g1 | 252 | 69 | 0 | 0 | 0 | 88.793 | 26.979 | 0 | 0 | 0 | -10.4231 | 7.35E-09 | 6.77E-07 | unkown |
| 443 | c47627_g2 | 23 | 659 | 0 | 0 | 0 | 6.762 | 29.68 | 0 | 0 | 0 | -11.5568 | 3.08E-09 | 3.03E-07 | unkown |
| 444 | c37378_g1 | 56 | 251 | 0 | 0 | 0 | 3.274 | 15.854 | 0 | 0 | 0 | -10.3965 | 1.70E-08 | 1.45E-06 | unkown |
| 445 | c34949_g1 | 80 | 18 | 0 | 0 | 0 | 17.995 | 4.45 | 0 | 0 | 0 | -8.71197 | 4.94E-05 | 0.001723 | unkown |
| 446 | c36546_g1 | 47 | 12 | 1 | 0 | 0 | 22.821 | 6.509 | 0.526 | 0 | 0 | -6.09393 | 0.00144 | 0.029283 | unkown |
| 447 | c32510_g1 | 11 | 11 | 0 | 1 | 0 | 3.004 | 3.312 | 0 | 0.247 | 0 | -4.75327 | 0.014995 | 0.171967 | unkown |
| 448 | c52585_g4 | 156 | 107 | 0 | 0 | 0 | 14.92 | 10.866 | 0 | 0 | 0 | -10.1479 | 1.18E-08 | 1.04E-06 | unkown |
| 449 | c44858_g2 | 60 | 79 | 0 | 0 | 0 | 8.286 | 11.911 | 0 | 0 | 0 | -9.23914 | 2.48E-06 | 0.000129 | unkown |
| 450 | c1617_g1 | 31 | 27 | 0 | 0 | 0 | 14.194 | 13.795 | 0 | 0 | 0 | -7.97517 | 0.000271 | 0.007361 | unkown |
| 451 | c49593_g3 | 12 | 12 | 0 | 0 | 0 | 5.623 | 6.923 | 0 | 0 | 0 | -6.71232 | 0.004091 | 0.067153 | unkown |
| 452 | c47315_g12 | 15 | 13 | 0 | 0 | 0 | 8.015 | 7.782 | 0 | 0 | 0 | -6.93063 | 0.00287 | 0.050595 | unkown |
| 453 | c45660_g7 | 27 | 24 | 0 | 1 | 0 | 23.433 | 23.585 | 0 | 0.806 | 0 | -5.95651 | 0.001489 | 0.030131 | unkown |
| 454 | c39715_g1 | 42 | 18 | 0 | 0 | 0 | 5.951 | 2.784 | 0 | 0 | 0 | -8.01371 | 0.000288 | 0.007769 | unkown |
| 455 | c38144_g1 | 54 | 32 | 0 | 0 | 1 | 13.866 | 9.055 | 0 | 0 | 0.186 | -6.76174 | 0.000172 | 0.004976 | unkown |
| 456 | c45756_g1 | 38 | 16 | 0 | 0 | 0 | 20.472 | 8.776 | 0 | 0 | 0 | -7.86213 | 0.000443 | 0.011146 | unkown |
| 457 | c50690_g5 | 469 | 23 | 1 | 1 | 0 | 200.693 | 4.905 | 0.179 | 1.205 | 0 | -8.37564 | 1.50E-07 | 1.04E-05 | unkown |
| 458 | c27749_g1 | 143 | 68 | 0 | 0 | 0 | 13.966 | 7.234 | 0 | 0 | 0 | -9.82512 | 1.32E-07 | 9.26E-06 | unkown |
| 459 | c36772_g1 | 24 | 17 | 0 | 0 | 0 | 19.518 | 25.406 | 0 | 0 | 0 | -7.47405 | 0.00099 | 0.021641 | unkown |
| 460 | c47904_g4 | 19 | 29 | 0 | 0 | 0 | 8.499 | 14.478 | 0 | 0 | 0 | -7.71176 | 0.000564 | 0.013577 | unkown |
| 461 | c45462_g1 | 195 | 61 | 0 | 0 | 0 | 120.342 | 39.801 | 0 | 0 | 0 | -10.0984 | 4.71E-08 | 3.67E-06 | unkown |
| 462 | c45657_g5 | 12 | 43 | 0 | 0 | 0 | 1.096 | 4.295 | 0 | 0 | 0 | -7.91809 | 0.000477 | 0.011834 | unkown |
| 463 | c50012_g3 | 34 | 17 | 0 | 0 | 0 | 7.574 | 4.16 | 0 | 0 | 0 | -7.78232 | 0.00052 | 0.012707 | unkown |
| 464 | c47829_g1 | 12 | 12 | 0 | 1 | 1 | 2.378 | 2.608 | 0 | 0.184 | 0.146 | -4.14427 | 0.0207 | 0.215075 | unkown |
| 465 | c26231_g1 | 45 | 15 | 1 | 1 | 1 | 6.52 | 2.867 | 0.252 | 0.207 | 0.097 | -4.89077 | 0.002438 | 0.04465 | unkown |
| 466 | c40714_g1 | 22 | 27 | 0 | 0 | 0 | 2.42 | 3.229 | 0 | 0 | 0 | -7.73815 | 0.000506 | 0.012409 | unkown |
| 467 | c13233_g1 | 12 | 70 | 0 | 0 | 0 | 2.847 | 18.317 | 0 | 0 | 0 | -8.49685 | 0.000135 | 0.004046 | unkown |
| 468 | c53696_g4 | 11 | 13 | 0 | 0 | 0 | 5.453 | 7.213 | 0 | 0 | 0 | -6.71476 | 0.004124 | 0.067623 | unkown |
| 469 | c25836_g1 | 138 | 274 | 0 | 1 | 0 | 24.8 | 45.855 | 0 | 0.239 | 0 | -8.98066 | 7.35E-10 | 8.15E-08 | unkown |
| 470 | c63382_g1 | 38 | 37 | 0 | 0 | 0 | 9.909 | 10.639 | 0 | 0 | 0 | -8.34641 | 8.79E-05 | 0.002819 | unkown |
| 471 | c44744_g9 | 31 | 18 | 0 | 0 | 0 | 4.47 | 2.836 | 0 | 0 | 0 | -7.72697 | 0.000562 | 0.013539 | unkown |
| 472 | c50529_g3 | 36 | 57 | 1 | 0 | 0 | 12.471 | 17.117 | 1.062 | 0 | 0 | -6.782 | 0.000124 | 0.003776 | unkown |
| 473 | c39812_g1 | 11 | 40 | 0 | 0 | 0 | 4.129 | 13.474 | 0 | 0 | 0 | -7.80974 | 0.000664 | 0.015496 | unkown |
| 474 | c67573_g1 | 107 | 151 | 0 | 1 | 0 | 21.996 | 34.078 | 0 | 0.192 | 0 | -8.30009 | 7.21E-08 | 5.42E-06 | unkown |
| 475 | c46221_g1 | 172 | 50 | 0 | 0 | 0 | 32.004 | 10.204 | 0 | 0 | 0 | -9.89223 | 1.82E-07 | 1.24E-05 | unkown |
| 476 | c50665_g5 | 13 | 12 | 1 | 0 | 0 | 8.67 | 9.014 | 0.715 | 0 | 0 | -4.87753 | 0.011932 | 0.147206 | unkown |
| 477 | c5177_g1 | 16 | 15 | 0 | 0 | 0 | 10.193 | 10.742 | 0 | 0 | 0 | -7.07749 | 0.002245 | 0.041953 | unkown |
| 478 | c48762_g3 | 68 | 116 | 1 | 0 | 0 | 5.339 | 9.893 | 0.084 | 0 | 0 | -7.7748 | 1.62E-06 | 8.76E-05 | unkown |
| 479 | c49850_g3 | 81 | 19 | 0 | 0 | 1 | 26.722 | 6.944 | 0 | 0 | 0.234 | -6.96885 | 0.000152 | 0.004471 | unkown |
| 480 | c72901_g1 | 48 | 44 | 0 | 0 | 0 | 63.21 | 66.149 | 0 | 0 | 0 | -8.6394 | 2.99E-05 | 0.001124 | unkown |
| 481 | c23881_g1 | 141 | 17 | 0 | 0 | 1 | 64.805 | 8.724 | 0 | 0 | 0.218 | -7.62065 | 2.82E-05 | 0.00107 | unkown |
| 482 | c36152_g1 | 20 | 24 | 1 | 1 | 1 | 12.514 | 18.162 | 0.673 | 0.575 | 0.445 | -4.46334 | 0.005697 | 0.086444 | unkown |
| 483 | c42113_g5 | 109 | 24 | 1 | 0 | 0 | 24.515 | 5.992 | 0.295 | 0 | 0 | -7.27053 | 4.05E-05 | 0.001454 | unkown |
| 484 | c36353_g1 | 116 | 16 | 0 | 1 | 1 | 26.181 | 3.974 | 0 | 0.207 | 0.162 | -6.56562 | 0.000117 | 0.003579 | unkown |
| 485 | c44851_g2 | 360 | 165 | 0 | 0 | 0 | 141.597 | 96.833 | 0 | 0 | 0 | -11.1387 | 5.56E-12 | 9.08E-10 | unkown |
| 486 | c44051_g2 | 41 | 64 | 0 | 0 | 0 | 8.058 | 10.504 | 0 | 0 | 0 | -8.83774 | 1.54E-05 | 0.000629 | unkown |
| 487 | c47531_g2 | 25 | 11 | 0 | 0 | 1 | 3.445 | 1.656 | 0 | 0 | 0.097 | -5.51455 | 0.005176 | 0.080416 | unkown |
| 488 | c13737_g1 | 15 | 17 | 0 | 1 | 0 | 2.164 | 2.68 | 0 | 0.136 | 0 | -5.29114 | 0.006522 | 0.09566 | unkown |
| 489 | c34028_g2 | 140 | 105 | 0 | 1 | 0 | 31.705 | 26.151 | 0 | 0.207 | 0 | -8.2157 | 1.15E-07 | 8.25E-06 | unkown |
| 490 | c44468_g2 | 69.51 | 75 | 0 | 0 | 0 | 8.457 | 12.491 | 0 | 0 | 0 | -9.29684 | 1.75E-06 | 9.41E-05 | unkown |
| 491 | c2519_g1 | 12 | 15 | 0 | 0 | 0 | 2.605 | 3.57 | 0 | 0 | 0 | -6.884 | 0.003215 | 0.055385 | unkown |
| 492 | c52532_g3 | 223 | 27 | 0 | 0 | 0 | 36.517 | 4.843 | 0 | 0 | 0 | -10.0559 | 3.70E-07 | 2.34E-05 | unkown |
| 493 | c52026_g4 | 91 | 237 | 0 | 0 | 0 | 12.044 | 33.985 | 0 | 0 | 0 | -10.486 | 2.65E-09 | 2.65E-07 | unkown |
| 494 | c18836_g1 | 17 | 24 | 0 | 0 | 0 | 2.249 | 3.456 | 0 | 0 | 0 | -7.48436 | 0.001001 | 0.02185 | unkown |
| 495 | c44394_g1 | 38 | 34 | 0 | 0 | 0 | 12.671 | 11.011 | 0 | 0 | 0 | -8.2864 | 0.000106 | 0.003311 | unkown |
| 496 | c50175_g1 | 15 | 30 | 1 | 0 | 1 | 5.638 | 12.532 | 0.41 | 0 | 0.275 | -5.01122 | 0.00421 | 0.06861 | unkown |
| 497 | c55938_g1 | 315 | 106 | 0 | 0 | 0 | 110.419 | 41.219 | 0 | 0 | 0 | -10.8164 | 2.51E-10 | 3.05E-08 | unkown |
| 498 | c14794_g1 | 386 | 297 | 0 | 0 | 0 | 187.467 | 161.213 | 0 | 0 | 0 | -11.5257 | 2.64E-14 | 6.27E-12 | unkown |
| 499 | c14219_g1 | 58 | 39 | 0 | 0 | 0 | 5.809 | 4.243 | 0 | 0 | 0 | -8.71085 | 2.42E-05 | 0.000934 | unkown |
| 500 | c7219_g1 | 140 | 45 | 0 | 0 | 0 | 19.049 | 6.685 | 0 | 0 | 0 | -9.63066 | 6.43E-07 | 3.86E-05 | unkown |
| 501 | c49793_g2 | 60 | 43 | 0 | 0 | 1 | 23.576 | 23.409 | 0 | 0 | 0.332 | -7.02204 | 6.44E-05 | 0.002158 | unkown |
| 502 | c4099_g1 | 14 | 15 | 0 | 0 | 1 | 7.261 | 8.703 | 0 | 0 | 0.372 | -5.2175 | 0.00815 | 0.111884 | unkown |
| 503 | c45799_g5 | 35 | 79 | 0 | 0 | 0 | 9.311 | 22.633 | 0 | 0 | 0 | -8.96128 | 1.22E-05 | 0.000515 | unkown |
| 504 | c49074_g5 | 16 | 27 | 0 | 0 | 0 | 1.68 | 3.105 | 0 | 0 | 0 | -7.55527 | 0.000893 | 0.019867 | unkown |
| 505 | c53740_g2 | 50 | 140 | 0 | 0 | 0 | 4.342 | 14.281 | 0 | 0 | 0 | -9.69987 | 4.34E-07 | 2.70E-05 | unkown |
| 506 | c33077_g2 | 16 | 20 | 1 | 0 | 0 | 2.975 | 4.067 | 0.2 | 0 | 0 | -5.40555 | 0.00469 | 0.074437 | unkown |
| 507 | c42586_g1 | 101 | 151 | 0 | 0 | 0 | 19.96 | 31.129 | 0 | 0 | 0 | -10.0984 | 1.84E-08 | 1.56E-06 | unkown |
| 508 | c39169_g1 | 309 | 54 | 0 | 0 | 0 | 54.683 | 10.618 | 0 | 0 | 0 | -10.5961 | 6.83E-09 | 6.32E-07 | unkown |
| 509 | c13777_g1 | 22 | 29 | 0 | 0 | 0 | 3.36 | 4.833 | 0 | 0 | 0 | -7.79667 | 0.000455 | 0.0114 | unkown |
| 510 | c10091_g1 | 91 | 96 | 1 | 1 | 0 | 48.646 | 57.487 | 0.579 | 0.495 | 0 | -7.01338 | 2.21E-06 | 0.000116 | unkown |
| 511 | c16992_g1 | 612 | 203 | 0 | 0 | 0 | 237.737 | 87.344 | 0 | 0 | 0 | -11.7688 | 2.40E-14 | 5.77E-12 | unkown |
| 512 | c77477_g1 | 82 | 67 | 0 | 0 | 0 | 7.517 | 6.685 | 0 | 0 | 0 | -9.33184 | 1.48E-06 | 8.07E-05 | unkown |
| 513 | c76280_g1 | 37 | 26 | 0 | 0 | 0 | 8.67 | 6.706 | 0 | 0 | 0 | -8.09077 | 0.0002 | 0.005676 | unkown |
| 514 | c46941_g7 | 14 | 82 | 0 | 0 | 0 | 2.705 | 17.365 | 0 | 0 | 0 | -8.72376 | 6.62E-05 | 0.002207 | unkown |
| 515 | c37853_g1 | 11 | 14 | 0 | 0 | 0 | 15.233 | 22.157 | 0 | 0 | 0 | -6.77419 | 0.00375 | 0.062547 | unkown |
| 516 | c20681_g1 | 19 | 20 | 0 | 0 | 0 | 2.534 | 2.908 | 0 | 0 | 0 | -7.40825 | 0.001123 | 0.023908 | unkown |
| 517 | c41862_g6 | 67 | 91 | 0 | 1 | 0 | 16.272 | 24.33 | 0 | 0.223 | 0 | -7.59186 | 4.30E-06 | 0.000207 | unkown |
| 518 | c51310_g1 | 223 | 113 | 0 | 0 | 1 | 16.628 | 9.159 | 0 | 0 | 0.057 | -8.70804 | 6.55E-09 | 6.08E-07 | unkown |
| 519 | c59120_g1 | 42 | 30 | 0 | 0 | 0 | 3.346 | 2.598 | 0 | 0 | 0 | -8.28298 | 0.00011 | 0.003407 | unkown |
| 520 | c43851_g2 | 64 | 13 | 0 | 0 | 0 | 7.944 | 1.759 | 0 | 0 | 0 | -8.36412 | 0.000168 | 0.00488 | unkown |
| 521 | c47564_g1 | 14 | 13 | 1 | 1 | 1 | 5.638 | 5.402 | 0.442 | 0.375 | 0.291 | -3.75946 | 0.02198 | 0.224333 | unkown |
| 522 | c51119_g3 | 133 | 30 | 0 | 0 | 0 | 32.16 | 9.179 | 0 | 0 | 0 | -9.44453 | 2.61E-06 | 0.000135 | unkown |
| 523 | c13617_g1 | 15 | 14 | 0 | 0 | 0 | 1.481 | 1.501 | 0 | 0 | 0 | -6.98195 | 0.002654 | 0.047717 | unkown |
| 524 | c18837_g2 | 16 | 64 | 0 | 0 | 0 | 7.83 | 34.989 | 0 | 0 | 0 | -8.45803 | 0.000112 | 0.003466 | unkown |
| 525 | c53022_g1 | 28 | 18 | 0 | 0 | 0 | 3.474 | 3.291 | 0 | 0 | 0 | -7.63774 | 0.000683 | 0.015873 | unkown |
| 526 | c32109_g1 | 56 | 37 | 0 | 0 | 0 | 89.591 | 67.784 | 0 | 0 | 0 | -8.64999 | 3.11E-05 | 0.001163 | unkown |
| 527 | c43194_g6 | 49 | 118 | 0 | 0 | 0 | 5.31 | 13.712 | 0 | 0 | 0 | -9.51207 | 9.76E-07 | 5.58E-05 | unkown |
| 528 | c51465_g4 | 25 | 21 | 0 | 0 | 0 | 2.392 | 2.194 | 0 | 0 | 0 | -7.64171 | 0.000657 | 0.015365 | unkown |
| 529 | c31821_g1 | 124 | 28 | 0 | 1 | 0 | 34.78 | 8.672 | 0 | 0.583 | 0 | -7.51089 | 1.67E-05 | 0.000676 | unkown |
| 530 | c45397_g9 | 15 | 87 | 0 | 0 | 0 | 2.121 | 13.402 | 0 | 0 | 0 | -8.81096 | 4.88E-05 | 0.001707 | unkown |
| 531 | c31287_g1 | 15 | 53 | 0 | 0 | 0 | 0.911 | 3.477 | 0 | 0 | 0 | -8.22298 | 0.000213 | 0.005984 | unkown |
| 532 | c48190_g2 | 411 | 195 | 0 | 0 | 0 | 37.157 | 23.088 | 0 | 0 | 0 | -11.3461 | 6.33E-13 | 1.18E-10 | unkown |
| 533 | c43865_g3 | 13 | 11 | 1 | 1 | 0 | 15.561 | 15.006 | 1.273 | 1.109 | 0 | -4.04608 | 0.021111 | 0.218036 | unkown |
| 534 | c11722_g1 | 15 | 19 | 0 | 1 | 0 | 2.335 | 3.239 | 0 | 0.144 | 0 | -5.37967 | 0.005321 | 0.081989 | unkown |
| 535 | c33604_g2 | 11 | 34 | 0 | 0 | 0 | 0.897 | 3.032 | 0 | 0 | 0 | -7.62823 | 0.000902 | 0.019997 | unkown |
| 536 | c40555_g8 | 45 | 48 | 0 | 1 | 0 | 10.692 | 12.553 | 0 | 0.215 | 0 | -6.82401 | 0.00011 | 0.003401 | unkown |
| 537 | c20280_g1 | 15 | 31 | 1 | 0 | 0 | 4.57 | 10.442 | 0.337 | 0 | 0 | -5.76521 | 0.00242 | 0.044433 | unkown |
| 538 | c51119_g2 | 175 | 121 | 1 | 0 | 0 | 31.648 | 26.348 | 0.326 | 0 | 0 | -8.45303 | 2.04E-08 | 1.71E-06 | unkown |
| 539 | c17898_g1 | 85 | 12 | 0 | 0 | 0 | 10.806 | 1.666 | 0 | 0 | 0 | -8.69342 | 8.08E-05 | 0.002622 | unkown |
| 540 | c40292_g1 | 48 | 25 | 0 | 0 | 1 | 38.41 | 11.311 | 0 | 0 | 0.744 | -6.52623 | 0.000401 | 0.010216 | unkown |
| 541 | c47839_g5 | 28 | 26 | 1 | 1 | 0 | 12.799 | 13.567 | 0.537 | 0.455 | 0 | -5.21272 | 0.00207 | 0.039308 | unkown |
| 542 | c34232_g2 | 15 | 48 | 0 | 0 | 0 | 3.161 | 11.456 | 0 | 0 | 0 | -8.11209 | 0.000276 | 0.00747 | unkown |
| 543 | c40555_g4 | 248 | 166 | 0 | 0 | 0 | 31.591 | 22.033 | 0 | 0 | 0 | -10.8016 | 6.96E-11 | 9.32E-09 | unkown |
| 544 | c46572_g3 | 148 | 45 | 0 | 0 | 1 | 68.521 | 19.745 | 0 | 0 | 0.267 | -7.91031 | 2.16E-06 | 0.000114 | unkown |
| 545 | c47616_g7 | 15 | 13 | 0 | 0 | 0 | 12.243 | 12.977 | 0 | 0 | 0 | -6.93063 | 0.002869 | 0.050595 | unkown |
| 546 | c53110_g6 | 124 | 26 | 1 | 0 | 0 | 96.168 | 22.778 | 0.831 | 0 | 0 | -7.44473 | 2.08E-05 | 0.000817 | unkown |
| 547 | c40191_g3 | 85 | 43 | 0 | 0 | 0 | 6.962 | 3.829 | 0 | 0 | 0 | -9.10596 | 5.60E-06 | 0.00026 | unkown |
| 548 | c7428_g1 | 23 | 14 | 0 | 1 | 0 | 2.805 | 1.863 | 0 | 0.112 | 0 | -5.49004 | 0.004637 | 0.073854 | unkown |
| 549 | c28095_g1 | 15 | 26 | 1 | 0 | 0 | 1.95 | 3.695 | 0.147 | 0 | 0 | -5.59704 | 0.003472 | 0.05877 | unkown |
| 550 | c50628_g7 | 20 | 16 | 0 | 0 | 0 | 1.893 | 1.645 | 0 | 0 | 0 | -7.28936 | 0.001512 | 0.030518 | unkown |
| 551 | c811_g1 | 44 | 30 | 0 | 0 | 0 | 4.584 | 3.405 | 0 | 0 | 0 | -8.32169 | 9.76E-05 | 0.003084 | unkown |
| 552 | c53460_g3 | 21 | 17 | 0 | 0 | 0 | 6.079 | 1.666 | 0 | 0 | 0 | -7.36705 | 0.001223 | 0.025685 | unkown |
| 553 | c39182_g1 | 24 | 14 | 0 | 0 | 0 | 2.378 | 1.511 | 0 | 0 | 0 | -7.36228 | 0.001361 | 0.028061 | unkown |
| 554 | c49821_g3 | 84 | 145 | 0 | 0 | 1 | 9.197 | 17.8 | 0 | 0 | 0.081 | -8.17796 | 2.47E-07 | 1.63E-05 | unkown |
| 555 | c37550_g1 | 250 | 26 | 0 | 0 | 0 | 39.862 | 4.533 | 0 | 0 | 0 | -10.1977 | 2.41E-07 | 1.59E-05 | unkown |
| 556 | c50317_g6 | 16 | 13 | 0 | 0 | 0 | 12.685 | 11.642 | 0 | 0 | 0 | -6.9799 | 0.002661 | 0.047803 | unkown |
| 557 | c49726_g2 | 48 | 15 | 0 | 0 | 0 | 18.308 | 13.981 | 0 | 0 | 0 | -8.07998 | 0.000281 | 0.00758 | unkown |
| 558 | c1978_g1 | 12 | 21 | 1 | 0 | 0 | 2.876 | 5.557 | 0.263 | 0 | 0 | -5.28452 | 0.006189 | 0.092101 | unkown |
| 559 | c71669_g1 | 18 | 22 | 0 | 0 | 0 | 3.431 | 4.595 | 0 | 0 | 0 | -7.4468 | 0.00105 | 0.022714 | unkown |
| 560 | c5002_g1 | 17 | 11 | 0 | 0 | 0 | 4 | 2.846 | 0 | 0 | 0 | -6.92639 | 0.002958 | 0.051725 | unkown |
| 561 | c46119_g1 | 23 | 12 | 0 | 0 | 0 | 19.931 | 8.869 | 0 | 0 | 0 | -7.24286 | 0.001759 | 0.034443 | unkown |
| 562 | c33629_g1 | 326 | 33 | 0 | 0 | 1 | 106.817 | 12.967 | 0 | 0 | 1.148 | -8.79481 | 1.45E-07 | 1.01E-05 | unkown |
| 563 | c52131_g1 | 30 | 88 | 1 | 0 | 0 | 9.268 | 27.621 | 0.431 | 0 | 0 | -7.13385 | 4.97E-05 | 0.001733 | unkown |
| 564 | c51430_g2 | 38 | 17 | 0 | 1 | 0 | 8.656 | 4.264 | 0 | 0.207 | 0 | -6.05502 | 0.001359 | 0.028029 | unkown |
| 565 | c24774_g1 | 476 | 424 | 1 | 0 | 0 | 178.47 | 178.92 | 0.547 | 0 | 0 | -10.0741 | 1.41E-15 | 4.21E-13 | unkown |
| 566 | c34908_g1 | 82 | 45 | 0 | 0 | 0 | 30.651 | 18.69 | 0 | 0 | 0 | -9.09581 | 5.57E-06 | 0.000259 | unkown |
| 567 | c33535_g1 | 15 | 22 | 1 | 1 | 0 | 4.157 | 6.53 | 0.2 | 0.168 | 0 | -4.67449 | 0.007303 | 0.103594 | unkown |
| 568 | c28541_g1 | 21 | 21 | 0 | 0 | 1 | 10.734 | 12.005 | 0 | 0 | 0.364 | -5.7455 | 0.002841 | 0.050294 | unkown |
| 569 | c15274_g1 | 22 | 27 | 0 | 0 | 0 | 12.243 | 15.823 | 0 | 0 | 0 | -7.73815 | 0.000506 | 0.012409 | unkown |
| 570 | c79597_g1 | 14 | 11 | 0 | 0 | 0 | 14.507 | 12.957 | 0 | 0 | 0 | -6.76713 | 0.003723 | 0.062218 | unkown |
| 571 | c68031_g1 | 96 | 78 | 0 | 1 | 0 | 10.193 | 9.024 | 0 | 0.096 | 0 | -7.72312 | 2.05E-06 | 0.000109 | unkown |
| 572 | c41489_g2 | 89 | 49 | 0 | 0 | 1 | 45.543 | 30.798 | 0 | 0 | 0.332 | -7.43675 | 1.30E-05 | 0.000545 | unkown |
| 573 | c14726_g1 | 23 | 17 | 0 | 0 | 0 | 10.222 | 8.434 | 0 | 0 | 0 | -7.43926 | 0.001063 | 0.022928 | unkown |
| 574 | c44784_g4 | 15 | 16 | 0 | 0 | 0 | 24.003 | 29.308 | 0 | 0 | 0 | -7.07941 | 0.002251 | 0.042013 | unkown |
| 575 | c46144_g4 | 53 | 13 | 0 | 0 | 0 | 7.802 | 2.09 | 0 | 0 | 0 | -8.14427 | 0.000274 | 0.00743 | unkown |
| 576 | c42978_g1 | 17 | 23 | 1 | 0 | 0 | 6.193 | 9.128 | 0.6 | 0 | 0 | -5.5583 | 0.003582 | 0.060345 | unkown |
| 577 | c34597_g1 | 26 | 14 | 1 | 1 | 1 | 6.606 | 3.922 | 0.274 | 0.231 | 0.186 | -4.31467 | 0.007975 | 0.110413 | unkown |
| 578 | c12584_g1 | 17 | 45 | 0 | 0 | 0 | 3.417 | 9.935 | 0 | 0 | 0 | -8.08689 | 0.000264 | 0.007189 | unkown |
| 579 | c27863_g1 | 19 | 15 | 0 | 0 | 0 | 5.609 | 4.895 | 0 | 0 | 0 | -7.20725 | 0.001754 | 0.034362 | unkown |
| 580 | c20104_g1 | 44 | 34 | 0 | 0 | 0 | 8.328 | 7.058 | 0 | 0 | 0 | -8.39928 | 7.41E-05 | 0.002439 | unkown |
| 581 | c49638_g1 | 1792 | 494 | 0 | 0 | 0 | 350.689 | 89.372 | 0 | 0 | 0 | -13.2543 | 2.63E-21 | 1.63E-18 | unkown |
| 582 | c48847_g3 | 12 | 29 | 0 | 0 | 1 | 1.751 | 4.626 | 0 | 0 | 0.105 | -5.72501 | 0.003594 | 0.060526 | unkown |
| 583 | c50878_g2 | 88 | 18 | 0 | 0 | 0 | 30.609 | 7.192 | 0 | 0 | 0 | -8.82403 | 3.61E-05 | 0.001323 | unkown |
| 584 | c50317_g7 | 256 | 93 | 0 | 0 | 0 | 17.995 | 7.099 | 0 | 0 | 0 | -10.5469 | 1.58E-09 | 1.65E-07 | unkown |
| 585 | c33757_g1 | 37 | 17 | 0 | 0 | 0 | 4.043 | 2.018 | 0 | 0 | 0 | -7.86327 | 0.000427 | 0.010819 | unkown |
| 586 | c44437_g2 | 21 | 51 | 0 | 0 | 0 | 6.193 | 16.641 | 0 | 0 | 0 | -8.30086 | 0.000134 | 0.004017 | unkown |
| 587 | c4106_g1 | 18 | 14 | 0 | 0 | 0 | 6.022 | 5.195 | 0 | 0 | 0 | -7.12019 | 0.00207 | 0.039308 | unkown |
| 588 | c49020_g9 | 19 | 24 | 0 | 0 | 0 | 2.363 | 5.857 | 0 | 0 | 0 | -7.55106 | 0.000843 | 0.018901 | unkown |
| 589 | c47348_g10 | 58 | 40 | 0 | 0 | 0 | 3.175 | 2.38 | 0 | 0 | 0 | -8.72599 | 2.30E-05 | 0.000893 | unkown |
| 590 | c32301_g1 | 226 | 257 | 0 | 0 | 0 | 97.321 | 118.856 | 0 | 0 | 0 | -11.0322 | 6.47E-12 | 1.05E-09 | unkown |
| 591 | c37295_g1 | 31 | 18 | 0 | 0 | 0 | 4.157 | 2.629 | 0 | 0 | 0 | -7.72697 | 0.000562 | 0.013539 | unkown |
| 592 | c17190_g1 | 40 | 28 | 0 | 0 | 0 | 3.801 | 2.898 | 0 | 0 | 0 | -8.2005 | 0.000141 | 0.004205 | unkown |
| 593 | c44555_g10 | 45 | 47 | 0 | 0 | 0 | 6.093 | 6.944 | 0 | 0 | 0 | -8.64141 | 2.98E-05 | 0.001122 | unkown |
| 594 | c20429_g1 | 22 | 12 | 1 | 1 | 0 | 7.446 | 4.502 | 0.368 | 0.311 | 0 | -4.53825 | 0.00998 | 0.129899 | unkown |
| 595 | c41878_g1 | 170 | 61 | 1 | 0 | 0 | 28.502 | 6.095 | 0.936 | 0 | 0 | -8.08108 | 4.70E-07 | 2.90E-05 | unkown |
| 596 | c54085_g3 | 23 | 102 | 1 | 1 | 0 | 1.794 | 8.652 | 0.084 | 0.072 | 0 | -6.44408 | 9.01E-05 | 0.002875 | unkown |
| 597 | c50272_g2 | 15 | 109 | 0 | 0 | 0 | 2.434 | 19.394 | 0 | 0 | 0 | -9.09383 | 2.35E-05 | 0.000909 | unkown |
| 598 | c34671_g1 | 16 | 11 | 0 | 0 | 0 | 3.488 | 2.639 | 0 | 0 | 0 | -6.87523 | 0.003259 | 0.055985 | unkown |
| 599 | c48464_g3 | 14 | 16 | 0 | 0 | 0 | 7.076 | 11.736 | 0 | 0 | 0 | -7.03349 | 0.002484 | 0.045312 | unkown |
| 600 | c45522_g7 | 102 | 61 | 0 | 0 | 0 | 81.305 | 49.043 | 0 | 0 | 0 | -9.4565 | 8.72E-07 | 5.06E-05 | unkown |
| 601 | c17143_g1 | 22 | 51 | 0 | 0 | 0 | 4.869 | 12.398 | 0 | 0 | 0 | -8.3201 | 0.000122 | 0.003721 | unkown |
| 602 | c4638_g1 | 25 | 18 | 1 | 0 | 0 | 6.179 | 4.905 | 0.274 | 0 | 0 | -5.6533 | 0.002861 | 0.050512 | unkown |
| 603 | c45270_g2 | 299 | 75 | 0 | 0 | 1 | 49.586 | 11.146 | 0 | 0 | 0.089 | -8.85549 | 9.66E-09 | 8.70E-07 | unkown |
| 604 | c31740_g2 | 58 | 15 | 0 | 1 | 0 | 111.415 | 25.127 | 0 | 1.165 | 0 | -6.45554 | 0.000611 | 0.01452 | unkown |
| 605 | c45096_g2 | 83 | 14 | 0 | 0 | 0 | 28.786 | 4.947 | 0 | 0 | 0 | -8.69473 | 6.86E-05 | 0.002283 | unkown |
| 606 | c52705_g3 | 117 | 550 | 0 | 1 | 0 | 36.645 | 187.892 | 0 | 0.287 | 0 | -9.68554 | 2.61E-11 | 3.77E-09 | unkown |
| 607 | c44858_g1 | 99 | 151 | 0 | 0 | 0 | 10.35 | 17.2 | 0 | 0 | 0 | -10.0872 | 2.05E-08 | 1.72E-06 | unkown |
| 608 | c37255_g1 | 39 | 79 | 0 | 0 | 0 | 6.207 | 13.754 | 0 | 0 | 0 | -9.0095 | 9.24E-06 | 0.000404 | unkown |
| 609 | c43009_g3 | 157.32 | 49 | 0 | 0 | 0 | 14.763 | 5.081 | 0 | 0 | 0 | -9.78524 | 2.82E-07 | 1.83E-05 | unkown |
| 610 | c52478_g1 | 17 | 16 | 0 | 0 | 0 | 4.513 | 4.44 | 0 | 0 | 0 | -7.1671 | 0.001894 | 0.03653 | unkown |
| 611 | c25227_g1 | 12 | 22 | 0 | 0 | 0 | 2.577 | 5.433 | 0 | 0 | 0 | -7.21957 | 0.001787 | 0.034892 | unkown |
| 612 | c2423_g1 | 17 | 32 | 0 | 0 | 0 | 5.723 | 11.932 | 0 | 0 | 0 | -7.74433 | 0.00056 | 0.013495 | unkown |
| 613 | c19370_g1 | 23 | 12 | 0 | 0 | 0 | 25.569 | 15.182 | 0 | 0 | 0 | -7.24286 | 0.001759 | 0.034443 | unkown |
| 614 | c41830_g3 | 153 | 37 | 0 | 0 | 0 | 52.12 | 9.531 | 0 | 0 | 0 | -9.66601 | 8.08E-07 | 4.73E-05 | unkown |
| 615 | c50685_g1 | 36 | 28 | 0 | 0 | 0 | 9.724 | 8.01 | 0 | 0 | 0 | -8.11493 | 0.000184 | 0.005287 | unkown |
| 616 | c20936_g1 | 17 | 15 | 0 | 0 | 0 | 4.485 | 5.599 | 0 | 0 | 0 | -7.12206 | 0.002052 | 0.039009 | unkown |
| 617 | c43946_g4 | 95 | 64 | 0 | 0 | 0 | 79.654 | 69.461 | 0 | 0 | 0 | -9.42247 | 9.74E-07 | 5.57E-05 | unkown |
| 618 | c50151_g1 | 155 | 12 | 0 | 0 | 1 | 37.371 | 3.187 | 0 | 0 | 0.178 | -7.69978 | 3.58E-05 | 0.001314 | unkown |
| 619 | c4816_g1 | 17 | 12 | 0 | 0 | 0 | 10.265 | 7.42 | 0 | 0 | 0 | -6.97784 | 0.002701 | 0.04833 | unkown |
| 620 | c46732_g7 | 54 | 13 | 0 | 0 | 1 | 5.495 | 1.438 | 0 | 0 | 0.073 | -6.39689 | 0.000854 | 0.019095 | unkown |
| 621 | c44926_g2 | 127 | 68 | 1 | 1 | 0 | 37.77 | 19.3 | 0.21 | 0.144 | 0 | -7.06323 | 2.09E-06 | 0.00011 | unkown |
| 622 | c701_g1 | 35 | 56 | 0 | 0 | 0 | 5.652 | 9.893 | 0 | 0 | 0 | -8.63212 | 3.47E-05 | 0.00128 | unkown |
| 623 | c73538_g1 | 16 | 38 | 0 | 0 | 0 | 2.249 | 5.837 | 0 | 0 | 0 | -7.88698 | 0.000434 | 0.010961 | unkown |
| 624 | c32966_g1 | 20 | 43 | 0 | 0 | 0 | 1.794 | 4.212 | 0 | 0 | 0 | -8.10726 | 0.000224 | 0.006237 | unkown |
| 625 | c42710_g1 | 22 | 18 | 0 | 0 | 1 | 6.122 | 5.423 | 0 | 0 | 0.275 | -5.67288 | 0.003315 | 0.056765 | unkown |
| 626 | c46208_g1 | 48 | 173 | 0 | 0 | 0 | 5.381 | 21.132 | 0 | 0 | 0 | -9.92054 | 1.92E-07 | 1.30E-05 | unkown |
| 627 | c78835_g1 | 15 | 47 | 0 | 0 | 0 | 1.609 | 5.495 | 0 | 0 | 0 | -8.08885 | 0.000289 | 0.007793 | unkown |
| 628 | c46389_g4 | 675 | 120 | 0 | 0 | 0 | 194.101 | 37.804 | 0 | 0 | 0 | -11.7267 | 9.14E-13 | 1.67E-10 | unkown |
| 629 | c40555_g5 | 32 | 27 | 0 | 0 | 0 | 6.891 | 6.396 | 0 | 0 | 0 | -7.99925 | 0.000254 | 0.006961 | unkown |
| 630 | c4560_g1 | 17 | 25 | 0 | 0 | 0 | 2.192 | 3.529 | 0 | 0 | 0 | -7.51953 | 0.000931 | 0.020559 | unkown |
| 631 | c4948_g1 | 112 | 54 | 0 | 0 | 0 | 6.563 | 3.436 | 0 | 0 | 0 | -9.4797 | 9.06E-07 | 5.23E-05 | unkown |
| 632 | c33492_g1 | 15 | 13 | 0 | 0 | 0 | 12.713 | 12.47 | 0 | 0 | 0 | -6.93063 | 0.002869 | 0.050595 | unkown |
| 633 | c28984_g1 | 170 | 26 | 1 | 0 | 0 | 43.564 | 8.879 | 0.284 | 0 | 0 | -7.82915 | 5.95E-06 | 0.000275 | unkown |
| 634 | c36412_g1 | 73 | 16 | 0 | 0 | 0 | 21.739 | 5.568 | 0 | 0 | 0 | -8.57312 | 7.96E-05 | 0.002586 | unkown |
| 635 | c49793_g6 | 100 | 27 | 0 | 0 | 0 | 72.849 | 22.188 | 0 | 0 | 0 | -9.08692 | 1.03E-05 | 0.000444 | unkown |
| 636 | c67227_g1 | 28 | 26 | 0 | 0 | 0 | 6.848 | 7.006 | 0 | 0 | 0 | -7.87347 | 0.000351 | 0.009142 | unkown |
| 637 | c15701_g1 | 144 | 57 | 0 | 0 | 0 | 39.236 | 17.138 | 0 | 0 | 0 | -9.75272 | 2.49E-07 | 1.64E-05 | unkown |
| 638 | c50529_g4 | 18 | 61 | 0 | 0 | 0 | 18.493 | 71.262 | 0 | 0 | 0 | -8.43823 | 0.000105 | 0.003282 | unkown |
| 639 | c11852_g1 | 68 | 75 | 0 | 0 | 0 | 23.59 | 28.863 | 0 | 0 | 0 | -9.27729 | 1.94E-06 | 0.000103 | unkown |
| 640 | c24745_g1 | 48 | 28 | 0 | 0 | 0 | 9.04 | 5.775 | 0 | 0 | 0 | -8.35776 | 9.14E-05 | 0.002905 | unkown |
| 641 | c38553_g1 | 14 | 22 | 0 | 0 | 0 | 2.933 | 5.102 | 0 | 0 | 0 | -7.29936 | 0.001471 | 0.02984 | unkown |
| 642 | c51646_g4 | 15 | 20 | 0 | 0 | 0 | 3.516 | 7.192 | 0 | 0 | 0 | -7.2566 | 0.001565 | 0.03134 | unkown |
| 643 | c20335_g1 | 69 | 44 | 1 | 1 | 0 | 8.115 | 5.64 | 0.126 | 0.112 | 0 | -6.27458 | 7.73E-05 | 0.002532 | unkown |
| 644 | c24246_g1 | 28 | 42 | 0 | 0 | 0 | 2.847 | 4.895 | 0 | 0 | 0 | -8.25372 | 0.000127 | 0.003841 | unkown |
| 645 | c38609_g1 | 235 | 59 | 1 | 0 | 0 | 78.543 | 17.552 | 0.431 | 0 | 0 | -8.42597 | 1.12E-07 | 8.05E-06 | unkown |
| 646 | c42113_g6 | 33 | 15 | 0 | 0 | 0 | 6.677 | 3.332 | 0 | 0 | 0 | -7.69401 | 0.000657 | 0.015374 | unkown |
| 647 | c50185_g4 | 12 | 28 | 0 | 0 | 0 | 2.648 | 6.789 | 0 | 0 | 0 | -7.45582 | 0.001232 | 0.025846 | unkown |
| 648 | c14432_g1 | 26 | 22 | 0 | 0 | 0 | 9.567 | 8.983 | 0 | 0 | 0 | -7.70291 | 0.000568 | 0.013635 | unkown |
| 649 | c30189_g3 | 34 | 12 | 1 | 0 | 0 | 5.453 | 2.101 | 0.179 | 0 | 0 | -5.73909 | 0.002806 | 0.049833 | unkown |
| 650 | c45343_g5 | 20 | 11 | 0 | 0 | 0 | 9.41 | 4.626 | 0 | 0 | 0 | -7.06977 | 0.002388 | 0.044007 | unkown |
| 651 | c43775_g2 | 84.89 | 16.1 | 0 | 0 | 0 | 8.941 | 1.852 | 0 | 0 | 0 | -8.75377 | 4.97E-05 | 0.001733 | unkown |
| 652 | c33510_g1 | 14 | 14 | 0 | 0 | 0 | 1.979 | 2.163 | 0 | 0 | 0 | -6.93275 | 0.002866 | 0.050575 | unkown |
| 653 | c58204_g1 | 30 | 31 | 0 | 0 | 0 | 11.603 | 13.329 | 0 | 0 | 0 | -8.05025 | 0.000222 | 0.006197 | unkown |
| 654 | c44589_g2 | 44.38 | 13 | 0 | 0 | 0 | 8.286 | 2.649 | 0 | 0 | 0 | -7.93557 | 0.000436 | 0.011004 | unkown |
| 655 | c45454_g3 | 46 | 11 | 0 | 0 | 0 | 6.492 | 1.697 | 0 | 0 | 0 | -7.93338 | 0.000491 | 0.01212 | unkown |
| 656 | c13446_g1 | 33 | 45 | 0 | 0 | 0 | 9.695 | 14.623 | 0 | 0 | 0 | -8.40795 | 7.27E-05 | 0.002401 | unkown |
| 657 | c34827_g2 | 32 | 65 | 0 | 1 | 0 | 2.192 | 7.927 | 0 | 0.064 | 0 | -6.89401 | 0.000105 | 0.003278 | unkown |
| 658 | c25749_g1 | 147 | 273 | 0 | 0 | 0 | 31.776 | 64.856 | 0 | 0 | 0 | -10.838 | 8.35E-11 | 1.10E-08 | unkown |
| 659 | c32969_g1 | 59 | 30 | 0 | 0 | 0 | 12.585 | 7.037 | 0 | 0 | 0 | -8.58296 | 4.28E-05 | 0.001525 | unkown |
| 660 | c27825_g1 | 13 | 32 | 0 | 0 | 0 | 1.125 | 3.001 | 0 | 0 | 0 | -7.62555 | 0.000816 | 0.018394 | unkown |
| 661 | c30039_g1 | 55 | 12 | 0 | 0 | 0 | 19.575 | 4.564 | 0 | 0 | 0 | -8.16477 | 0.000277 | 0.007494 | unkown |
| 662 | c51189_g6 | 43 | 95 | 0 | 0 | 0 | 11.261 | 27.424 | 0 | 0 | 0 | -9.23616 | 3.61E-06 | 0.000178 | unkown |
| 663 | c25596_g1 | 31 | 138 | 0 | 1 | 0 | 6.62 | 32.361 | 0 | 0.2 | 0 | -7.70317 | 8.09E-06 | 0.000361 | unkown |
| 664 | c49150_g3 | 20 | 26 | 0 | 0 | 0 | 4.912 | 7.11 | 0 | 0 | 0 | -7.64829 | 0.000668 | 0.015586 | unkown |
| 665 | c31385_g1 | 108 | 25 | 0 | 0 | 1 | 30.167 | 7.71 | 0 | 0 | 0.202 | -7.37647 | 3.53E-05 | 0.001298 | unkown |
| 666 | c43227_g1 | 24 | 44 | 0 | 1 | 0 | 2.292 | 4.543 | 0 | 0.088 | 0 | -6.38098 | 0.000549 | 0.013294 | unkown |
| 667 | c76458_g1 | 18 | 61 | 0 | 0 | 0 | 2.477 | 9.159 | 0 | 0 | 0 | -8.43823 | 0.000105 | 0.003281 | unkown |
| 668 | c36372_g1 | 27 | 32 | 0 | 0 | 0 | 8.471 | 14.105 | 0 | 0 | 0 | -8.00443 | 0.000256 | 0.006999 | unkown |
| 669 | c52728_g8 | 84 | 196 | 0 | 1 | 0 | 5.552 | 14.054 | 0 | 0.064 | 0 | -8.42511 | 5.78E-08 | 4.44E-06 | unkown |
| 670 | c27197_g1 | 15 | 56 | 0 | 0 | 0 | 4.855 | 20.066 | 0 | 0 | 0 | -8.28565 | 0.000184 | 0.005287 | unkown |
| 671 | c47358_g8 | 17 | 31 | 0 | 0 | 0 | 5.752 | 11.622 | 0 | 0 | 0 | -7.71428 | 0.000594 | 0.014171 | unkown |
| 672 | c49158_g1 | 238 | 266 | 0 | 0 | 0 | 26.395 | 28.231 | 0 | 0 | 0 | -11.0933 | 3.35E-12 | 5.59E-10 | unkown |
| 673 | c22227_g1 | 13 | 11 | 0 | 0 | 1 | 1.822 | 1.687 | 0 | 0 | 0.105 | -4.9437 | 0.012349 | 0.150574 | unkown |
| 674 | c11758_g1 | 15 | 17 | 0 | 0 | 0 | 2.634 | 3.27 | 0 | 0 | 0 | -7.12579 | 0.002068 | 0.039281 | unkown |
| 675 | c52532_g2 | 83 | 75 | 0 | 0 | 0 | 27.918 | 23.885 | 0 | 0 | 0 | -9.41788 | 9.39E-07 | 5.40E-05 | unkown |
| 676 | c38983_g2 | 15 | 17 | 1 | 0 | 0 | 4.442 | 5.568 | 0.326 | 0 | 0 | -5.23474 | 0.006717 | 0.09755 | unkown |
| 677 | c35192_g1 | 15 | 25 | 0 | 0 | 0 | 2.278 | 4.15 | 0 | 0 | 0 | -7.45131 | 0.001114 | 0.023778 | unkown |
| 678 | c26715_g1 | 24 | 20 | 0 | 0 | 0 | 3.972 | 3.684 | 0 | 0 | 0 | -7.57779 | 0.000772 | 0.017583 | unkown |
| 679 | c20532_g2 | 88 | 65 | 1 | 0 | 1 | 28.288 | 21.205 | 0.358 | 0 | 0.234 | -6.75868 | 1.06E-05 | 0.000454 | unkown |
| 680 | c55412_g1 | 12 | 13 | 0 | 0 | 0 | 2.833 | 3.374 | 0 | 0 | 0 | -6.77184 | 0.003701 | 0.061912 | unkown |
| 681 | c18372_g1 | 16 | 17 | 0 | 0 | 0 | 2.975 | 3.456 | 0 | 0 | 0 | -7.16891 | 0.00189 | 0.036474 | unkown |
| 682 | c46732_g3 | 43 | 25 | 0 | 0 | 0 | 6.321 | 4.015 | 0 | 0 | 0 | -8.19778 | 0.000149 | 0.004398 | unkown |
| 683 | c48911_g1 | 32 | 25 | 0 | 0 | 0 | 15.959 | 13.226 | 0 | 0 | 0 | -7.94853 | 0.000293 | 0.007876 | unkown |
| 684 | c52719_g1 | 130.48 | 103.14 | 0 | 1 | 0 | 8.314 | 7.13 | 0 | 0.056 | 0 | -8.14406 | 1.85E-07 | 1.26E-05 | unnamed protein product |
| 685 | c31581_g1 | 109 | 110 | 0 | 1 | 1 | 30.95 | 34.503 | 0 | 0.263 | 0.202 | -7.31215 | 5.99E-07 | 3.63E-05 | unnamed protein product |
| 686 | c39454_g1 | 1269 | 143 | 0 | 0 | 0 | 67.097 | 9.893 | 0 | 0 | 0 | -12.552 | 1.41E-14 | 3.56E-12 | unnamed protein product |
| 687 | c55075_g1 | 56 | 118 | 0 | 0 | 0 | 3.787 | 8.662 | 0 | 0 | 0 | -9.56948 | 6.17E-07 | 3.72E-05 | unnamed protein product |
| 688 | c37601_g1 | 1993 | 213 | 0 | 0 | 1 | 106.475 | 12.346 | 0 | 0 | 0.04 | -11.4003 | 6.38E-16 | 1.97E-13 | unnamed protein product |
| 689 | c37764_g1 | 414 | 265 | 0 | 0 | 0 | 62.584 | 43.796 | 0 | 0 | 0 | -11.5144 | 4.18E-14 | 9.62E-12 | unnamed protein product |
| 690 | c43517_g5 | 67 | 27 | 0 | 0 | 0 | 10.919 | 4.812 | 0 | 0 | 0 | -8.6585 | 3.81E-05 | 0.001384 | unnamed protein product |
| 691 | c35000_g1 | 438 | 326 | 0 | 1 | 0 | 58.299 | 47.377 | 0 | 0.12 | 0 | -9.85773 | 2.78E-14 | 6.55E-12 | unnamed protein product |
| 692 | c32365_g1 | 456 | 333 | 0 | 1 | 0 | 69.603 | 59.971 | 0 | 0.128 | 0 | -9.90393 | 1.54E-14 | 3.82E-12 | unnamed protein product |
| 693 | c43433_g1 | 429 | 301 | 0 | 0 | 0 | 42.041 | 32.112 | 0 | 0 | 0 | -11.6203 | 8.94E-15 | 2.34E-12 | unnamed protein product |
| 694 | c32946_g1 | 112 | 11 | 0 | 0 | 0 | 16.885 | 2.504 | 0 | 0 | 0 | -9.03303 | 3.73E-05 | 0.001358 | UPF0591 membrane protein |
| 695 | c42060_g3 | 16 | 28 | 0 | 0 | 0 | 3.787 | 7.296 | 0 | 0 | 0 | -7.58878 | 0.00083 | 0.018666 | Wall-associated receptor kinase 2 |
| 696 | c52613_g3 | 28.98 | 95.84 | 0 | 0 | 0 | 2.135 | 7.668 | 0 | 0 | 0 | -9.09856 | 9.60E-06 | 0.000417 | Wall-associated receptor kinase 2 |
| 697 | c22507_g2 | 462 | 50 | 0 | 1 | 0 | 77.76 | 9.21 | 0 | 0.152 | 0 | -9.25723 | 5.99E-09 | 5.59E-07 | Zeatin O-glucosyltransferase |
