# Supplementary material for: Differential Gene Expression between Leaf and Rhizome in Atractylodes lancea: A Comparative Transcriptome Analysis
Source: Front Plant Sci. 2016 Mar 30;7:348. doi: 10.3389/fpls.2016.00348 (PMC4811964; doi:10.3389/fpls.2016.00348)
Supplement: Supplementary file 6 [file Table6.docx]

**Supplementary Table 6** List of rhizome-specific expressed genes in *A. lancea* detected by transcriptome sequencing.

| No. | Gene_ID | L1  Exp | L2  Exp | R1  Exp | R2  Exp | R3  Exp | L1  FPKM | L2  FPKM | R1  FPKM | R2  FPKM | R3  FPKM | logFC | PValue | FDR | Blast_hit |
| --- | --- | --- | --- | --- | --- | --- | --- | --- | --- | --- | --- | --- | --- | --- | --- |
| 1 | c33075_g1 | 0 | 0 | 146 | 47 | 60 | 0 | 0 | 21.933 | 5.889 | 5.967 | 9.434119 | 2.73E-05 | 0.001038 | (-)-beta-caryophyllene synthase |
| 2 | c37987_g1 | 1 | 0 | 176 | 891 | 22 | 0.057 | 0 | 14.475 | 52.229 | 2.175 | 9.088507 | 4.01E-06 | 0.000194 | (+)-pulegone reductase |
| 3 | c51564_g1 | 0 | 0 | 120 | 283 | 351 | 0 | 0 | 14.875 | 27.595 | 28.242 | 10.85573 | 2.52E-09 | 2.52E-07 | (E)-beta-farnesene synthase |
| 4 | c79295_g1 | 0 | 1 | 23 | 14 | 18 | 0 | 0.103 | 2.325 | 1.181 | 1.205 | 4.75888 | 0.02088 | 0.216334 | deacetylbaccatin III acetyltransferase |
| 5 | c46763_g2 | 0 | 0 | 85 | 95 | 130 | 0 | 0 | 5.397 | 5.003 | 5.458 | 9.61432 | 2.90E-06 | 0.000148 | 11S globulin seed storage protein 2 |
| 6 | c770_g1 | 0 | 0 | 389 | 266 | 60 | 0 | 0 | 62.497 | 35.647 | 6.379 | 10.9592 | 9.06E-08 | 6.63E-06 | 14 kDa proline-rich protein |
| 7 | c31020_g1 | 1 | 1 | 17 | 102 | 248 | 0.071 | 0.083 | 1.368 | 6.815 | 13.203 | 6.464321 | 0.000154 | 0.004512 | 14-3-3 protein |
| 8 | c37969_g1 | 0 | 0 | 97 | 175 | 75 | 0 | 0 | 5.197 | 7.709 | 2.612 | 9.826191 | 2.17E-06 | 0.000114 | 1-acyl-sn-glycerol-3-phosphate acyltransferase |
| 9 | c35343_g1 | 0 | 0 | 890 | 1423 | 294 | 0 | 0 | 80.275 | 108.153 | 17.828 | 12.77307 | 1.40E-13 | 2.98E-11 | 21 kDa protein-like |
| 10 | c34643_g1 | 1 | 1 | 45 | 141 | 17 | 0.071 | 0.072 | 3.24 | 8.427 | 0.809 | 5.799928 | 0.001181 | 0.024923 | 2-hydroxyisoflavanone dehydratase |
| 11 | c36898_g1 | 0 | 0 | 22 | 58 | 33 | 0 | 0 | 1.368 | 2.993 | 1.358 | 8.172993 | 0.000926 | 0.020446 | 3-ketoacyl-CoA synthase |
| 12 | c19639_g1 | 0 | 0 | 17 | 129 | 23 | 0 | 0 | 0.799 | 5.043 | 0.72 | 8.766569 | 0.000623 | 0.014733 | 3-ketoacyl-CoA synthase 6-like isoform 1 |
| 13 | c18484_g1 | 1 | 0 | 24 | 27 | 14 | 0.142 | 0 | 3.556 | 3.224 | 1.172 | 5.032375 | 0.014208 | 0.166192 | 40S ribosomal protein S4 |
| 14 | c37464_g1 | 0 | 0 | 110 | 204 | 30 | 0 | 0 | 4.618 | 7.086 | 0.833 | 9.852766 | 1.14E-05 | 0.000484 | ABC transporter G family member 8-like |
| 15 | c37816_g1 | 0 | 0 | 90 | 46 | 14 | 0 | 0 | 4.029 | 1.708 | 0.412 | 8.717445 | 0.00056 | 0.013509 | adenine/guanine permease AZG1 |
| 16 | c34505_g1 | 0 | 0 | 68 | 131 | 46 | 0 | 0 | 5.376 | 8.586 | 2.401 | 9.330713 | 2.61E-05 | 0.000997 | aintegumenta |
| 17 | c35173_g1 | 0 | 0 | 17 | 21 | 12 | 0 | 0 | 1.662 | 1.708 | 0.776 | 7.047342 | 0.009464 | 0.124823 | hydrolase domain-containing protein |
| 18 | c8043_g1 | 0 | 0 | 20 | 57 | 44 | 0 | 0 | 17.757 | 43.683 | 25.962 | 8.247357 | 0.000729 | 0.016762 | alpha-1,4-galacturonosyltransferase 1 |
| 19 | c32631_g1 | 0 | 0 | 17 | 19 | 20 | 0 | 0 | 3.976 | 2.115 | 3.032 | 7.175161 | 0.006992 | 0.100414 | alpha-glucosidase |
| 20 | c44437_g4 | 0 | 0 | 119 | 262 | 145 | 0 | 0 | 95.024 | 180.077 | 76.909 | 10.39921 | 7.25E-08 | 5.43E-06 | Alpha-isocomene synthase |
| 21 | c21060_g1 | 0 | 1 | 21 | 29 | 26 | 0 | 0.662 | 13.455 | 15.904 | 11.053 | 5.188032 | 0.008971 | 0.120306 | alpha-tubulin 5 |
| 22 | c58272_g1 | 1 | 0 | 15 | 43 | 25 | 0.925 | 0 | 14.78 | 36.692 | 16.381 | 5.320559 | 0.008359 | 0.114045 | alpha-tubulin, partial |
| 23 | c35037_g1 | 1 | 0 | 39 | 33 | 23 | 0.085 | 0 | 3.65 | 2.562 | 1.423 | 5.580452 | 0.004416 | 0.071245 | Alpha-xylosidase 1 |
| 24 | c37126_g1 | 0 | 0 | 254 | 231 | 153 | 0 | 0 | 10.698 | 8.052 | 4.261 | 10.72642 | 7.52E-09 | 6.90E-07 | ankyrin repeat domain-containing |
| 25 | c40786_g1 | 0 | 0 | 2154 | 1019 | 1174 | 0 | 0 | 109.12 | 45.957 | 42.4 | 13.51023 | 7.42E-19 | 3.47E-16 | Anthranilate N-benzoyltransferase |
| 26 | c13499_g1 | 0 | 0 | 78 | 68 | 128 | 0 | 0 | 4.239 | 3.056 | 4.593 | 9.427493 | 8.28E-06 | 0.000368 | ethylene-responsive transcription factor |
| 27 | c28561_g1 | 1 | 0 | 42 | 148 | 19 | 0.185 | 0 | 8.658 | 25.528 | 2.595 | 6.702188 | 0.000665 | 0.015518 | Arabinogalactan peptide 20 |
| 28 | c17043_g1 | 0 | 0 | 12 | 30 | 22 | 0 | 0 | 1.441 | 2.985 | 1.738 | 7.343005 | 0.005626 | 0.085587 | arginine/serine-rich splicing factor |
| 29 | c45890_g1 | 0 | 1 | 58 | 282 | 36 | 0 | 0.031 | 2.146 | 8.642 | 0.881 | 7.519565 | 5.72E-05 | 0.00195 | ATP binding protein |
| 30 | c39612_g1 | 0 | 0 | 74 | 12 | 139 | 0 | 0 | 4.618 | 0.622 | 7.56 | 9.117727 | 0.000189 | 0.005398 | Auxin-induced protein 5NG4 |
| 31 | c34123_g1 | 0 | 0 | 38 | 51 | 238 | 0 | 0 | 3.398 | 3.91 | 14.756 | 9.573967 | 2.24E-05 | 0.000872 | auxin-induced protein 5NG4 |
| 32 | c38704_g1 | 0 | 0 | 63 | 86 | 64 | 0 | 0 | 8.416 | 9.656 | 5.765 | 9.107044 | 3.28E-05 | 0.001215 | Auxin-induced protein 6B |
| 33 | c40381_g1 | 0 | 0 | 181 | 526 | 173 | 0 | 0 | 13.244 | 31.721 | 8.401 | 11.15513 | 1.86E-09 | 1.91E-07 | auxin-responsive protein IAA12-like |
| 34 | c28448_g1 | 1 | 0 | 235 | 83 | 1210 | 0.085 | 0 | 23.259 | 6.823 | 79.213 | 9.381906 | 3.39E-08 | 2.73E-06 | Avr9/Cf-9 rapidly elicited protein 1 |
| 35 | c46152_g2 | 1 | 0 | 112 | 146 | 642 | 0.285 | 0 | 11.929 | 7.717 | 31.962 | 8.631446 | 6.26E-08 | 4.78E-06 | BAG family molecular chaperone regulator |
| 36 | c18176_g1 | 0 | 0 | 47 | 67 | 40 | 0 | 0 | 2.64 | 3.12 | 1.488 | 8.651678 | 0.000203 | 0.00574 | basic 7S globulin-like |
| 37 | c63135_g1 | 0 | 0 | 14 | 76 | 21 | 0 | 0 | 0.799 | 3.615 | 0.8 | 8.155373 | 0.001763 | 0.03449 | BEL1-like homeodomain protein 11 |
| 38 | c52908_g3 | 0 | 0 | 15 | 14 | 12 | 0 | 0 | 2.493 | 1.947 | 1.318 | 6.756963 | 0.014281 | 0.166694 | beta-1,3-galactosyltransferase 6 |
| 39 | c38871_g1 | 0 | 0 | 140 | 224 | 172 | 0 | 0 | 7.848 | 8.531 | 5.231 | 10.42396 | 3.22E-08 | 2.61E-06 | beta-1,3-glucanase |
| 40 | c28775_g1 | 1 | 0 | 27 | 53 | 20 | 0.142 | 0 | 4.166 | 6.823 | 2.046 | 5.632115 | 0.004519 | 0.072401 | beta-1,3-glucanase |
| 41 | c40173_g1 | 1 | 0 | 92 | 336 | 45 | 0.071 | 0 | 4.597 | 12.034 | 1.124 | 7.875465 | 1.18E-05 | 0.000498 | beta-galactosidase 3-like |
| 42 | c45161_g1 | 0 | 0 | 235 | 1096 | 44 | 0 | 0 | 47.296 | 167.78 | 5.134 | 11.82926 | 1.03E-07 | 7.45E-06 | bidirectional sugar transporter N3 |
| 43 | c44518_g2 | 0 | 1 | 15 | 28 | 17 | 0 | 0.083 | 1.347 | 2.019 | 1.083 | 4.855959 | 0.01771 | 0.193912 | bidirectional sugar transporter SWEET14 |
| 44 | c43112_g1 | 1 | 1 | 129 | 341 | 100 | 0.028 | 0.041 | 4.923 | 10.757 | 2.498 | 7.267821 | 1.04E-06 | 5.91E-05 | BTB/POZ domain-containing protein |
| 45 | c41442_g1 | 0 | 0 | 108 | 82 | 74 | 0 | 0 | 4.029 | 3.152 | 2.021 | 9.447388 | 9.20E-06 | 0.000402 | BTB/POZ domain-containing protein |
| 46 | c49212_g2 | 1 | 1 | 156 | 170 | 64 | 0.043 | 0.052 | 10.162 | 8.1 | 2.83 | 6.763471 | 9.72E-06 | 0.000421 | calmodulin binding protein |
| 47 | c35537_g2 | 1 | 1 | 18 | 11 | 68 | 0.199 | 0.228 | 4.008 | 2.051 | 10.034 | 4.581099 | 0.013384 | 0.159288 | CCHC-type integrase |
| 48 | c38726_g1 | 0 | 1 | 21 | 81 | 15 | 0 | 0.083 | 1.757 | 5.61 | 0.825 | 5.834948 | 0.004426 | 0.071338 | CDK |
| 49 | c35845_g1 | 0 | 1 | 227 | 424 | 619 | 0 | 0.114 | 21.06 | 33.093 | 40.799 | 9.189377 | 2.68E-11 | 3.86E-09 | ChNRRa |
| 50 | c15511_g1 | 1 | 0 | 541 | 601 | 149 | 0.142 | 0 | 85.43 | 79.162 | 15.573 | 9.368734 | 7.89E-10 | 8.69E-08 | class I heat shock family protein |
| 51 | c38847_g1 | 0 | 1 | 14 | 30 | 19 | 0 | 0.145 | 2.062 | 3.687 | 1.852 | 4.915402 | 0.015987 | 0.180245 | CLAVATA3/ESR (CLE)-related protein |
| 52 | c31534_g1 | 0 | 1 | 421 | 327 | 276 | 0 | 0.093 | 40.427 | 26.023 | 17.408 | 8.9872 | 1.87E-10 | 2.34E-08 | conserved hypothetical protein |
| 53 | c36698_g1 | 1 | 1 | 171 | 154 | 171 | 0.114 | 0.114 | 20.313 | 15.218 | 13.438 | 7.055471 | 5.43E-07 | 3.32E-05 | conserved hypothetical protein |
| 54 | c42823_g3 | 0 | 0 | 152 | 118 | 100 | 0 | 0 | 14.265 | 9.193 | 6.21 | 9.936681 | 8.55E-07 | 4.97E-05 | conserved hypothetical protein |
| 55 | c4496_g1 | 0 | 0 | 58 | 142 | 56 | 0 | 0 | 2.43 | 4.932 | 1.552 | 9.374856 | 2.05E-05 | 0.000808 | conserved hypothetical protein |
| 56 | c33153_g1 | 0 | 1 | 45 | 225 | 23 | 0 | 0.041 | 1.799 | 7.389 | 0.606 | 7.163328 | 0.000246 | 0.006761 | conserved hypothetical protein |
| 57 | c34303_g1 | 0 | 0 | 33 | 126 | 22 | 0 | 0 | 1.094 | 3.455 | 0.485 | 8.887881 | 0.000304 | 0.008109 | conserved hypothetical protein |
| 58 | c29256_g1 | 0 | 0 | 28 | 49 | 16 | 0 | 0 | 11.708 | 17.357 | 4.431 | 7.945186 | 0.00195 | 0.037431 | conserved hypothetical protein |
| 59 | c37721_g1 | 0 | 0 | 28 | 80 | 15 | 0 | 0 | 3.198 | 8.714 | 1.35 | 8.34215 | 0.001148 | 0.024364 | conserved hypothetical protein |
| 60 | c9256_g1 | 0 | 0 | 25 | 25 | 42 | 0 | 0 | 1.22 | 1.013 | 1.358 | 7.856891 | 0.001664 | 0.032949 | conserved hypothetical protein |
| 61 | c4534_g1 | 0 | 0 | 15 | 17 | 14 | 0 | 0 | 1.325 | 1.245 | 0.817 | 6.910355 | 0.011369 | 0.142096 | conserved hypothetical protein |
| 62 | c35464_g1 | 0 | 0 | 169 | 423 | 107 | 0 | 0 | 24.237 | 50.553 | 10.147 | 10.84181 | 2.74E-08 | 2.25E-06 | cyclin D3 |
| 63 | c42417_g1 | 1 | 1 | 102 | 172 | 39 | 0.057 | 0.062 | 9.531 | 12.409 | 2.312 | 6.438451 | 6.94E-05 | 0.002302 | cyclin-D4-1 |
| 64 | c31395_g1 | 0 | 0 | 40 | 65 | 26 | 0 | 0 | 2.493 | 3.36 | 1.075 | 8.433003 | 0.000506 | 0.012409 | cycloidea-like 8 |
| 65 | c29530_g1 | 1 | 1 | 14 | 23 | 21 | 0.185 | 0.197 | 2.788 | 3.838 | 2.773 | 3.939142 | 0.02892 | 0.269817 | cysteine protease-4 |
| 66 | c3888_g1 | 0 | 0 | 25 | 35 | 21 | 0 | 0 | 1.378 | 1.596 | 0.825 | 7.728419 | 0.00246 | 0.045002 | cytochrome P450 734A1-like |
| 67 | c42348_g2 | 0 | 0 | 28 | 107 | 322 | 0 | 0 | 4.86 | 15.489 | 36.958 | 10.04748 | 5.62E-06 | 0.000261 | Dehydrin ERD14 |
| 68 | c39657_g1 | 0 | 0 | 456 | 176 | 52 | 0 | 0 | 60.225 | 19.352 | 4.544 | 10.92399 | 3.16E-07 | 2.04E-05 | dicyanin blue copper protein precursor |
| 69 | c5745_g2 | 0 | 1 | 109 | 136 | 77 | 0 | 0.735 | 76.162 | 81.5 | 35.713 | 7.306943 | 1.18E-05 | 0.000498 | diminuto family protein |
| 70 | c36991_g1 | 1 | 0 | 354 | 549 | 477 | 0.114 | 0 | 28.613 | 37.019 | 23.512 | 9.371843 | 2.14E-12 | 3.67E-10 | DNA binding protein |
| 71 | c42188_g1 | 0 | 0 | 83 | 157 | 25 | 0 | 0 | 5.902 | 9.249 | 1.172 | 9.47353 | 4.23E-05 | 0.001511 | DNA binding protein |
| 72 | c21404_g1 | 0 | 1 | 62 | 100 | 90 | 0 | 0.145 | 8.984 | 12.082 | 8.635 | 6.903835 | 5.11E-05 | 0.001773 | DNA binding protein |
| 73 | c18729_g1 | 0 | 0 | 24 | 33 | 20 | 0 | 0 | 7.027 | 9.153 | 3.38 | 7.656243 | 0.002897 | 0.05092 | DNA binding protein |
| 74 | c24257_g1 | 0 | 0 | 71 | 41 | 61 | 0 | 0 | 13.507 | 6.584 | 7.075 | 8.822081 | 0.000114 | 0.003514 | DNA-binding protein ESCAROLA |
| 75 | c23583_g1 | 0 | 1 | 43 | 222 | 89 | 0 | 0.083 | 3.713 | 15.904 | 5.078 | 7.388595 | 2.62E-05 | 0.001 | dof zinc finger protein DOF3unkown4 |
| 76 | c37914_g3 | 1 | 0 | 80 | 178 | 122 | 0.342 | 0 | 29.486 | 55.453 | 29.803 | 7.509337 | 4.10E-06 | 0.000198 | dof zinc finger protein DOF4unkown6 |
| 77 | c32106_g1 | 1 | 0 | 50 | 28 | 31 | 0.171 | 0 | 9.615 | 4.501 | 3.946 | 5.779394 | 0.002836 | 0.050214 | E3 ubiquitin-protein ligase |
| 78 | c41410_g1 | 1 | 1 | 113 | 181 | 22 | 0.114 | 0.124 | 14.075 | 18.761 | 1.811 | 6.47197 | 0.000145 | 0.0043 | Early nodulin-like protein 1 |
| 79 | c51564_g2 | 0 | 0 | 30 | 78 | 118 | 0 | 0 | 4.913 | 10.901 | 13.074 | 9.099203 | 5.18E-05 | 0.001792 | E-beta-farnesene synthase 3 |
| 80 | c46436_g4 | 0 | 0 | 21 | 66 | 11 | 0 | 0 | 3.356 | 8.786 | 1.164 | 8.014497 | 0.00259 | 0.046805 | eIF4G eukaryotic initiation factor 4, |
| 81 | c733_g1 | 0 | 0 | 31 | 28 | 13 | 0 | 0 | 8.742 | 6.639 | 2.426 | 7.604207 | 0.003989 | 0.065773 | electron transporter |
| 82 | c23474_g1 | 0 | 1 | 79 | 284 | 21 | 0 | 0.041 | 3.629 | 10.797 | 0.639 | 7.571174 | 8.18E-05 | 0.002647 | Endoglucanase 11 |
| 83 | c47936_g2 | 0 | 0 | 210 | 286 | 280 | 0 | 0 | 50.757 | 57.983 | 44.817 | 10.95019 | 4.01E-10 | 4.67E-08 | epoxide hydrolase 2 |
| 84 | c24834_g1 | 0 | 0 | 188 | 121 | 112 | 0 | 0 | 15.685 | 8.379 | 6.177 | 10.1321 | 3.69E-07 | 2.34E-05 | Ethylene-responsive transcription factor |
| 85 | c34048_g1 | 1 | 0 | 58 | 64 | 31 | 0.142 | 0 | 8.931 | 8.219 | 3.161 | 6.267547 | 0.000785 | 0.017795 | ethylene-responsive transcription factor |
| 86 | c36697_g1 | 0 | 1 | 485 | 1638 | 97 | 0 | 0.083 | 37.05 | 103.836 | 4.924 | 10.10917 | 5.29E-09 | 4.98E-07 | Expansin-B15 |
| 87 | c24732_g1 | 0 | 0 | 12 | 58 | 18 | 0 | 0 | 1.252 | 5.011 | 1.237 | 7.820523 | 0.003304 | 0.05662 | formin-like protein 1-like |
| 88 | c48120_g2 | 0 | 0 | 33 | 82 | 74 | 0 | 0 | 20.629 | 43.842 | 30.7 | 8.884346 | 9.03E-05 | 0.002879 | Formin-like protein 2 |
| 89 | c34179_g1 | 0 | 0 | 40 | 13 | 11 | 0 | 0 | 18.82 | 19.934 | 3.485 | 7.479247 | 0.006852 | 0.098877 | fructan:fructan 1-fructosyltransferase |
| 90 | c46198_g5 | 0 | 1 | 16 | 17 | 25 | 0 | 0.166 | 5.102 | 5.578 | 5.07 | 4.779301 | 0.018984 | 0.203041 | gag-pol polyprotein, identical |
| 91 | c37217_g1 | 0 | 0 | 13 | 31 | 25 | 0 | 0 | 1.273 | 2.354 | 1.205 | 7.446704 | 0.004481 | 0.072035 | gag-pol polyprotein, identical |
| 92 | c32191_g2 | 1 | 0 | 31 | 69 | 15 | 0.1 | 0 | 3.65 | 6.751 | 1.164 | 5.849153 | 0.003668 | 0.061439 | GATA transcription factor 9 |
| 93 | c35417_g1 | 0 | 0 | 18 | 88 | 40 | 0 | 0 | 1.189 | 4.804 | 1.738 | 8.528773 | 0.000535 | 0.013026 | GDSL esterase/lipase At3g48460 |
| 94 | c16271_g1 | 0 | 0 | 50 | 102 | 30 | 0 | 0 | 23.091 | 39.988 | 9.185 | 8.907146 | 0.000146 | 0.004329 | GDSL esterase/lipase At4g10955 |
| 95 | c78134_g1 | 0 | 0 | 170 | 282 | 68 | 0 | 0 | 42.047 | 58.525 | 11.142 | 10.44018 | 3.03E-07 | 1.96E-05 | GDSL esterase/lipase At4g10955 |
| 96 | c36268_g1 | 0 | 0 | 44 | 46 | 20 | 0 | 0 | 3.293 | 2.857 | 0.986 | 8.206754 | 0.000956 | 0.021022 | gibberellin 3-beta-dioxygenase 4-like |
| 97 | c22194_g1 | 0 | 0 | 140 | 151 | 32 | 0 | 0 | 7.616 | 6.799 | 1.148 | 9.784992 | 1.10E-05 | 0.000469 | Glucan endo-1,3-beta-glucosidase |
| 98 | c36013_g1 | 0 | 1 | 14 | 109 | 11 | 0 | 0.176 | 2.441 | 15.856 | 1.269 | 6.022795 | 0.005482 | 0.083921 | Glycine-rich cell wall structural protein |
| 99 | c49973_g1 | 0 | 0 | 178 | 211 | 175 | 0 | 0 | 5.617 | 5.506 | 3.655 | 10.51285 | 1.58E-08 | 1.35E-06 | glycosyl hydrolase family 3 family |
| 100 | c29967_g1 | 0 | 1 | 14 | 12 | 11 | 0 | 0.166 | 2.367 | 1.692 | 1.229 | 4.188494 | 0.047696 | 0.369591 | growth-regulating factor 8-like |
| 101 | c39697_g1 | 1 | 0 | 12 | 13 | 24 | 0.1 | 0 | 1.252 | 1.133 | 1.666 | 4.536628 | 0.029743 | 0.275694 | GTP-binding ADP-ribosylation factor |
| 102 | c33244_g1 | 0 | 0 | 172 | 140 | 51 | 0 | 0 | 7.206 | 7.222 | 1.197 | 9.953077 | 2.88E-06 | 0.000147 | Heat shock 70 kDa protein 2 |
| 103 | c30841_g2 | 1 | 0 | 107 | 70 | 82 | 0.114 | 0 | 14.18 | 7.725 | 7.188 | 7.006437 | 4.38E-05 | 0.001554 | heat shock protein |
| 104 | c41327_g1 | 0 | 0 | 47 | 16 | 11 | 0 | 0 | 3.198 | 1.66 | 0.566 | 7.695345 | 0.004829 | 0.076174 | Heat shock protein 90 homolog |
| 105 | c45259_g1 | 0 | 0 | 192 | 153 | 74 | 0 | 0 | 13.76 | 8.275 | 3.396 | 10.14828 | 6.87E-07 | 4.09E-05 | Heavy-metal-associated protein |
| 106 | c68474_g1 | 1 | 0 | 24 | 84 | 11 | 0.214 | 0 | 5.639 | 16.535 | 1.714 | 5.89208 | 0.004846 | 0.076387 | high mobility group B protein 7 |
| 107 | c38449_g1 | 1 | 1 | 26 | 103 | 49 | 0.171 | 0.186 | 4.786 | 15.848 | 5.975 | 5.548119 | 0.00104 | 0.022529 | Histone H3 |
| 108 | c37054_g1 | 1 | 0 | 145 | 192 | 29 | 0.299 | 0 | 46.749 | 52.173 | 6.193 | 7.555833 | 3.09E-05 | 0.001157 | Histone H3 |
| 109 | c12908_g1 | 0 | 0 | 38 | 157 | 34 | 0 | 0 | 7.437 | 25.712 | 4.407 | 9.216541 | 9.18E-05 | 0.002915 | histone H4 |
| 110 | c35576_g1 | 0 | 0 | 203 | 279 | 109 | 0 | 0 | 16.158 | 21.347 | 6.266 | 10.61619 | 3.34E-08 | 2.69E-06 | Histone-lysine N-methyltransferase |
| 111 | c44573_g3 | 1 | 1 | 55 | 85 | 85 | 0.142 | 0.155 | 8.879 | 11.443 | 9.08 | 5.884574 | 0.00017 | 0.004914 | homeobox leucine-zipper protein |
| 112 | c20620_g1 | 0 | 0 | 138 | 181 | 75 | 0 | 0 | 19.367 | 21.155 | 6.962 | 10.03172 | 8.76E-07 | 5.08E-05 | HVA22-like protein a |
| 113 | c35102_g1 | 0 | 0 | 13 | 39 | 17 | 0 | 0 | 1.894 | 4.972 | 2.361 | 7.473697 | 0.005085 | 0.079347 | hypothetical protein ARALYDRAFT |
| 114 | c8987_g1 | 0 | 1 | 60 | 57 | 92 | 0 | 0.103 | 6.385 | 5.035 | 6.468 | 6.624002 | 0.000153 | 0.004489 | hypothetical protein MTR_1g110410 |
| 115 | c47428_g1 | 0 | 1 | 19 | 29 | 40 | 0 | 0.331 | 6.154 | 7.916 | 8.579 | 5.358052 | 0.006331 | 0.093587 | hypothetical protein MTR_3g110530 |
| 116 | c40955_g1 | 0 | 0 | 153 | 60 | 42 | 0 | 0 | 25.983 | 8.507 | 4.722 | 9.466647 | 3.11E-05 | 0.001163 | hypothetical protein MTR_5g080260 |
| 117 | c32023_g1 | 1 | 1 | 17 | 114 | 30 | 0.256 | 0.041 | 1.389 | 4.485 | 0.76 | 5.415103 | 0.002716 | 0.04857 | hypothetical protein MTR_5g095330 |
| 118 | c29526_g1 | 1 | 1 | 21 | 38 | 14 | 0.1 | 0.114 | 2.314 | 3.487 | 1.019 | 4.318546 | 0.018199 | 0.197703 | hypothetical protein POPTR_0001s25390g |
| 119 | c32565_g1 | 0 | 1 | 422 | 453 | 108 | 0 | 0.093 | 35.63 | 33.077 | 6.21 | 8.968003 | 1.09E-08 | 9.68E-07 | hypothetical protein POPTR_0006s00790g |
| 120 | c40306_g1 | 0 | 0 | 127 | 178 | 89 | 0 | 0 | 5.07 | 5.881 | 2.353 | 10.01702 | 6.62E-07 | 3.95E-05 | hypothetical protein POPTR_0006s15580g |
| 121 | c36026_g2 | 0 | 0 | 41 | 150 | 15 | 0 | 0 | 4.639 | 14.117 | 1.124 | 9.089402 | 0.000282 | 0.007609 | hypothetical protein POPTR_0008s07760g |
| 122 | c269_g1 | 0 | 0 | 19 | 16 | 12 | 0 | 0 | 2.22 | 1.556 | 0.93 | 6.969357 | 0.01102 | 0.138894 | hypothetical protein POPTR_0010s11290g |
| 123 | c1015_g1 | 0 | 0 | 109 | 49 | 23 | 0 | 0 | 9.299 | 3.471 | 1.302 | 8.98126 | 0.0002 | 0.005674 | hypothetical protein POPTR_0012s08540g |
| 124 | c36342_g2 | 1 | 0 | 547 | 126 | 1177 | 0.114 | 0 | 67.673 | 12.968 | 96.33 | 9.733986 | 4.18E-10 | 4.84E-08 | hypothetical protein POPTR_0014s01770g |
| 125 | c2304_g1 | 1 | 0 | 22 | 15 | 27 | 0.1 | 0 | 2.325 | 1.333 | 1.884 | 4.957335 | 0.014644 | 0.169272 | hypothetical protein POPTR_0014s09570g |
| 126 | c39049_g1 | 1 | 0 | 167 | 177 | 81 | 0.057 | 0 | 9.573 | 8.355 | 3.016 | 7.744071 | 2.22E-06 | 0.000117 | hypothetical protein POPTR_0015s09180g |
| 127 | c34371_g1 | 0 | 0 | 131 | 65 | 1176 | 0 | 0 | 6.449 | 2.649 | 38.252 | 11.59992 | 8.97E-08 | 6.58E-06 | hypothetical protein POPTR_0015s13020g |
| 128 | c40022_g1 | 1 | 1 | 222 | 83 | 198 | 0.142 | 0.155 | 34.325 | 10.701 | 20.262 | 7.087031 | 1.44E-06 | 7.91E-05 | hypothetical protein POPTR_0018s06230g |
| 129 | c49297_g6 | 1 | 1 | 44 | 29 | 16 | 0.1 | 0.103 | 4.65 | 2.546 | 1.116 | 4.652117 | 0.009829 | 0.128442 | hypothetical protein RCOM_0884570 |
| 130 | c49297_g3 | 0 | 0 | 26 | 26 | 20 | 0 | 0 | 1.715 | 1.42 | 0.873 | 7.566861 | 0.003447 | 0.058426 | hypothetical protein RCOM_0884570 |
| 131 | c18685_g1 | 0 | 0 | 29 | 42 | 15 | 0 | 0 | 9.036 | 11.02 | 3.097 | 7.840265 | 0.0024 | 0.044163 | hypothetical protein RCOM_1355100 |
| 132 | c45059_g1 | 1 | 1 | 170 | 155 | 68 | 0.085 | 0.062 | 13.076 | 9.4 | 2.862 | 6.779907 | 8.43E-06 | 0.000373 | hypothetical protein RCOM_1592640 |
| 133 | c44206_g1 | 0 | 0 | 89 | 262 | 70 | 0 | 0 | 2.851 | 6.943 | 1.48 | 10.10054 | 1.54E-06 | 8.37E-05 | hypothetical protein VITISV_000145 |
| 134 | c50513_g2 | 1 | 1 | 42 | 39 | 24 | 0.071 | 0.072 | 3.114 | 2.394 | 1.172 | 4.858116 | 0.005147 | 0.080052 | hypothetical protein VITISV_000333 |
| 135 | c45205_g2 | 0 | 0 | 23 | 61 | 34 | 0 | 0 | 1.988 | 4.373 | 1.941 | 8.236213 | 0.000781 | 0.017744 | hypothetical protein VITISV_000834 |
| 136 | c23468_g1 | 1 | 0 | 13 | 96 | 111 | 0.157 | 0 | 2.157 | 13.303 | 12.201 | 6.641574 | 0.000547 | 0.013267 | hypothetical protein VITISV_008439 |
| 137 | c47343_g1 | 0 | 0 | 48 | 55 | 24 | 0 | 0 | 2.998 | 3.16 | 1.043 | 8.407034 | 0.000551 | 0.013331 | hypothetical protein VITISV_009255 |
| 138 | c39546_g1 | 0 | 0 | 118 | 535 | 14 | 0 | 0 | 6.154 | 23.11 | 0.485 | 10.78988 | 8.96E-06 | 0.000394 | hypothetical protein VITISV_011425 |
| 139 | c53153_g2 | 0 | 0 | 150 | 427 | 77 | 0 | 0 | 54.755 | 131.686 | 18.629 | 10.7512 | 1.32E-07 | 9.27E-06 | hypothetical protein VITISV_011450 |
| 140 | c30082_g1 | 0 | 0 | 125 | 55 | 47 | 0 | 0 | 22.459 | 8.259 | 5.587 | 9.278554 | 4.19E-05 | 0.001498 | hypothetical protein VITISV_014692 |
| 141 | c36270_g1 | 1 | 1 | 111 | 208 | 112 | 0.043 | 0.041 | 4.523 | 7.006 | 3.016 | 6.852318 | 2.98E-06 | 0.000151 | hypothetical protein VITISV_016729 |
| 142 | c39834_g1 | 0 | 0 | 72 | 180 | 81 | 0 | 0 | 2.272 | 4.7 | 1.69 | 9.745559 | 3.55E-06 | 0.000176 | hypothetical protein VITISV_018267 |
| 143 | c42564_g1 | 0 | 0 | 237 | 32 | 31 | 0 | 0 | 33.379 | 3.759 | 2.886 | 9.755959 | 7.20E-05 | 0.00238 | hypothetical protein VITISV_018480 |
| 144 | c42221_g1 | 1 | 0 | 12 | 21 | 27 | 0.071 | 0 | 0.863 | 1.245 | 1.277 | 4.825045 | 0.018577 | 0.20007 | hypothetical protein VITISV_019713 |
| 145 | c38025_g1 | 0 | 0 | 150 | 319 | 20 | 0 | 0 | 4.124 | 7.254 | 0.364 | 10.36755 | 6.99E-06 | 0.000317 | hypothetical protein VITISV_021289 |
| 146 | c38122_g1 | 0 | 0 | 35 | 22 | 79 | 0 | 0 | 5.533 | 1.899 | 8.102 | 8.384989 | 0.000578 | 0.013846 | hypothetical protein VITISV_026093 |
| 147 | c32304_g2 | 1 | 0 | 96 | 204 | 42 | 0.157 | 0 | 16.263 | 28.856 | 4.714 | 7.421513 | 2.81E-05 | 0.001064 | hypothetical protein VITISV_030359 |
| 148 | c24257_g2 | 0 | 0 | 55 | 40 | 89 | 0 | 0 | 6.564 | 3.974 | 7.026 | 8.853567 | 9.35E-05 | 0.002964 | hypothetical protein VITISV_032602 |
| 149 | c53123_g3 | 0 | 1 | 180 | 300 | 62 | 0 | 0.279 | 50.578 | 70.879 | 11.538 | 8.085169 | 1.40E-06 | 7.70E-05 | hypothetical protein VITISV_036996 |
| 150 | c38323_g1 | 0 | 0 | 30 | 33 | 14 | 0 | 0 | 3.23 | 2.649 | 0.97 | 7.691373 | 0.003227 | 0.055538 | hypothetical protein VITISV_037041 |
| 151 | c22911_g1 | 0 | 1 | 438 | 225 | 305 | 0 | 0.072 | 31.033 | 13.215 | 14.287 | 8.904985 | 6.02E-10 | 6.79E-08 | hypothetical protein VITISV_037591 |
| 152 | c53687_g2 | 0 | 1 | 112 | 230 | 89 | 0 | 0.621 | 67.273 | 118.04 | 35.471 | 7.716761 | 2.52E-06 | 0.000131 | hypothetical protein VITISV_042402 |
| 153 | c14895_g1 | 0 | 0 | 59 | 46 | 15 | 0 | 0 | 4.829 | 3.12 | 0.809 | 8.36518 | 0.000939 | 0.020705 | hypothetical protein VITISV_042556 |
| 154 | c23056_g1 | 0 | 0 | 18 | 43 | 16 | 0 | 0 | 1.315 | 2.554 | 0.728 | 7.650432 | 0.003654 | 0.061241 | hypothetical protein VITISV_043672 |
| 155 | c41266_g1 | 0 | 0 | 238 | 612 | 76 | 0 | 0 | 8.616 | 18.322 | 1.819 | 11.26748 | 2.27E-08 | 1.88E-06 | inactive receptor kinase At5g58300 |
| 156 | c44218_g1 | 0 | 0 | 433 | 720 | 543 | 0 | 0 | 16.432 | 22.607 | 13.624 | 12.08388 | 2.17E-14 | 5.30E-12 | Indole-3-acetic acid-amido synthetase |
| 157 | c31753_g1 | 1 | 1 | 36 | 118 | 34 | 0.114 | 0.114 | 2.293 | 7.15 | 1.649 | 5.659823 | 0.000894 | 0.019883 | Indole-3-acetic acid-amido synthetase |
| 158 | c25312_g1 | 0 | 0 | 60 | 399 | 35 | 0 | 0 | 3.524 | 19.447 | 1.326 | 10.33228 | 9.15E-06 | 0.000401 | Interactor of constitutive active ROPs 4 |
| 159 | c30606_g1 | 1 | 0 | 105 | 363 | 25 | 0.071 | 0 | 8.416 | 24.148 | 1.326 | 7.950356 | 2.61E-05 | 0.000996 | isoflavone reductase-like protein-like |
| 160 | c22324_g1 | 0 | 0 | 25 | 110 | 16 | 0 | 0 | 1.462 | 5.315 | 0.614 | 8.626719 | 0.000772 | 0.017583 | kelch repeat protein |
| 161 | c27310_g1 | 1 | 1 | 110 | 155 | 78 | 0.028 | 0.031 | 3.861 | 4.493 | 1.811 | 6.545496 | 1.49E-05 | 0.000613 | kinase-like protein TMKL1-like |
| 162 | c9883_g1 | 1 | 0 | 13 | 11 | 11 | 0.1 | 0 | 1.399 | 0.982 | 0.784 | 4.123968 | 0.052065 | 0.390025 | Kinesin-like polypeptides |
| 163 | c32980_g1 | 1 | 0 | 1133 | 1661 | 1152 | 0.043 | 0 | 50.484 | 61.207 | 33.894 | 10.90667 | 2.31E-19 | 1.17E-16 | knotted-1-like protein 2 |
| 164 | c43762_g1 | 1 | 1 | 53 | 98 | 100 | 0.043 | 0.041 | 2.377 | 3.639 | 2.959 | 6.029086 | 0.000105 | 0.003284 | laccase-3-like |
| 165 | c38532_g2 | 0 | 0 | 16 | 386 | 30 | 0 | 0 | 0.579 | 11.451 | 0.712 | 10.1192 | 8.82E-05 | 0.002825 | Leucine-rich repeat receptor protein kinase |
| 166 | c25160_g1 | 1 | 0 | 16 | 33 | 16 | 0.085 | 0 | 1.431 | 2.442 | 0.946 | 4.9978 | 0.014803 | 0.170712 | leucine-rich repeat receptor protein kinase |
| 167 | c37686_g1 | 0 | 1 | 19 | 106 | 18 | 0 | 0.041 | 0.768 | 3.599 | 0.485 | 6.113427 | 0.002926 | 0.05135 | leucine-rich repeat receptor protein kinase |
| 168 | c50491_g9 | 0 | 0 | 39.51 | 191.84 | 91 | 0 | 0 | 6.501 | 26.342 | 9.913 | 9.670259 | 9.44E-06 | 0.000411 | leucine-rich repeat receptor protein kinase |
| 169 | c45678_g1 | 0 | 1 | 76 | 337 | 42 | 0 | 0.021 | 1.894 | 6.959 | 0.695 | 7.798619 | 2.01E-05 | 0.000793 | leucine-rich repeat receptor protein kinase |
| 170 | c37540_g1 | 0 | 1 | 52 | 65 | 29 | 0 | 0.041 | 2.072 | 2.139 | 0.76 | 6.179071 | 0.001009 | 0.021978 | leucine-rich repeat transmembrane kinase |
| 171 | c42465_g4 | 0 | 1 | 19 | 42 | 24 | 0 | 0.072 | 1.462 | 2.689 | 1.221 | 5.350391 | 0.007151 | 0.102197 | Lignin-forming anionic peroxidase |
| 172 | c58755_g1 | 0 | 0 | 16 | 27 | 26 | 0 | 0 | 1.546 | 2.171 | 1.666 | 7.453449 | 0.004112 | 0.06747 | LINE-1 retrotransposable element |
| 173 | c38707_g1 | 1 | 1 | 83 | 62 | 120 | 0.171 | 0.176 | 15.022 | 9.377 | 14.376 | 6.118786 | 7.55E-05 | 0.002481 | lipid binding protein |
| 174 | c34893_g1 | 0 | 0 | 60 | 24 | 11 | 0 | 0 | 8.679 | 3.104 | 1.213 | 8.061342 | 0.002499 | 0.045485 | LOB domain-containing protein |
| 175 | c43316_g1 | 0 | 0 | 19 | 56 | 31 | 0 | 0 | 1.757 | 4.301 | 1.9 | 8.077339 | 0.001251 | 0.026193 | long chain acyl-CoA synthetase 2 |
| 176 | c49994_g3 | 0 | 0 | 92 | 190 | 40 | 0 | 0 | 5.386 | 9.225 | 1.552 | 9.741171 | 1.00E-05 | 0.000433 | LRR receptor-like serine/threonine kinase |
| 177 | c33307_g1 | 0 | 0 | 407 | 511 | 172 | 0 | 0 | 23.143 | 18.641 | 5.288 | 11.51213 | 1.48E-10 | 1.89E-08 | Lysine histidine transporter-like 8 |
| 178 | c44274_g1 | 0 | 0 | 79 | 212 | 27 | 0 | 0 | 5.723 | 13.582 | 1.294 | 9.72364 | 2.44E-05 | 0.000941 | Lysine histidine transporter-like 8 |
| 179 | c24871_g1 | 0 | 0 | 15 | 17 | 11 | 0 | 0 | 5.912 | 5.666 | 2.87 | 6.829633 | 0.013279 | 0.158535 | major facilitator super family |
| 180 | c32747_g1 | 0 | 0 | 22 | 46 | 87 | 0 | 0 | 2.325 | 4.046 | 6.088 | 8.549273 | 0.000345 | 0.009023 | Major pollen allergen Ory s 1 precursor |
| 181 | c47242_g6 | 1 | 1 | 31 | 93 | 37 | 0.157 | 0.176 | 5.333 | 13.375 | 4.221 | 5.425396 | 0.001429 | 0.029164 | mature anther-specific protein LAT61 |
| 182 | c46381_g1 | 0 | 1 | 298 | 550 | 196 | 0 | 0.114 | 33.652 | 51.774 | 15.403 | 9.004369 | 8.89E-10 | 9.74E-08 | Metal tolerance protein 4 |
| 183 | c41533_g1 | 0 | 0 | 29 | 129 | 49 | 0 | 0 | 1.347 | 4.94 | 1.496 | 9.04412 | 0.000108 | 0.003365 | Methyl-CpG-binding domain-containing |
| 184 | c32475_g1 | 1 | 0 | 13 | 32 | 26 | 0.071 | 0 | 0.968 | 1.971 | 1.277 | 5.081898 | 0.012284 | 0.150074 | methyltransferase PMT16 |
| 185 | c22641_g1 | 0 | 0 | 17 | 14 | 30.27 | 0 | 0 | 1.725 | 1.628 | 2.709 | 7.26076 | 0.006112 | 0.091275 | methyltransferase PMT26 |
| 186 | c33812_g1 | 0 | 1 | 562 | 845 | 283 | 0 | 0.3 | 167.399 | 211.861 | 55.846 | 9.715399 | 6.17E-12 | 1.00E-09 | Mini zinc finger protein 1 |
| 187 | c46327_g3 | 0 | 0 | 23 | 24 | 41 | 0 | 0 | 3.45 | 2.913 | 3.946 | 7.788379 | 0.001989 | 0.037992 | MLO-like protein 12 |
| 188 | c18994_g1 | 0 | 0 | 46 | 111 | 243 | 0 | 0 | 27.414 | 56.514 | 96.087 | 9.895074 | 2.57E-06 | 0.000133 | Myb-like protein X |
| 189 | c47266_g3 | 0 | 1 | 20 | 34 | 74 | 0 | 0.342 | 6.796 | 9.752 | 16.672 | 5.851688 | 0.002355 | 0.043473 | NBS-LRR protein |
| 190 | c33341_g1 | 0 | 0 | 1288 | 1395 | 1569 | 0 | 0 | 201.261 | 181.793 | 162.234 | 13.4095 | 1.23E-21 | 7.93E-19 | nonspecific lipid transfer protein |
| 191 | c28135_g1 | 0 | 0 | 136 | 251 | 31 | 0 | 0 | 31.632 | 48.941 | 4.778 | 10.13795 | 4.99E-06 | 0.000235 | non-specific lipid-transfer protein |
| 192 | c27882_g1 | 0 | 0 | 47 | 23 | 48 | 0 | 0 | 6.606 | 2.697 | 4.463 | 8.255994 | 0.000709 | 0.01636 | Ocs element-binding factor |
| 193 | c30961_g1 | 0 | 0 | 47 | 160 | 68 | 0 | 0 | 2.525 | 7.118 | 2.418 | 9.458077 | 1.72E-05 | 0.000694 | hydroxypalmitate O-feruloyl transferase |
| 194 | c37834_g1 | 0 | 1 | 54 | 60 | 37 | 0 | 0.135 | 7.469 | 6.847 | 3.355 | 6.217486 | 0.000762 | 0.017394 | ORA |
| 195 | c35647_g1 | 1 | 0 | 15 | 85 | 117 | 0.071 | 0 | 1.22 | 5.722 | 6.274 | 6.615524 | 0.000496 | 0.012209 | ORA |
| 196 | c44607_g1 | 0 | 0 | 40 | 48 | 52 | 0 | 0 | 3.471 | 3.455 | 3.072 | 8.483864 | 0.000289 | 0.007788 | Os10g0357350 |
| 197 | c27466_g1 | 0 | 0 | 38 | 124 | 71 | 0 | 0 | 3.892 | 10.566 | 4.819 | 9.203772 | 3.67E-05 | 0.001341 | PDF |
| 198 | c43821_g2 | 1 | 1 | 74 | 16 | 104 | 0.057 | 0.062 | 4.471 | 0.798 | 4.156 | 5.666039 | 0.001074 | 0.023107 | Pectate lyase |
| 199 | c43821_g3 | 1 | 0 | 66 | 339 | 22 | 0.043 | 0 | 3.461 | 11.707 | 0.606 | 7.729797 | 7.68E-05 | 0.002518 | pectate lyase |
| 200 | c46512_g1 | 0 | 1 | 139 | 294 | 79 | 0 | 0.093 | 14.044 | 23.206 | 4.956 | 7.979951 | 1.36E-06 | 7.50E-05 | pectate lyase 18-like |
| 201 | c43821_g5 | 0 | 0 | 41 | 154 | 13 | 0 | 0 | 1.809 | 5.634 | 0.38 | 9.105268 | 0.000317 | 0.008404 | pectate lyase 8 |
| 202 | c34453_g1 | 1 | 0 | 239 | 374 | 493 | 0.214 | 0 | 72.796 | 80.135 | 120.174 | 9.018708 | 5.80E-11 | 7.86E-09 | asparagine amidase A |
| 203 | c48336_g3 | 0 | 0 | 71 | 84 | 33 | 0 | 0 | 16.61 | 15.441 | 5.401 | 8.974947 | 9.58E-05 | 0.003031 | peptidyl-prolyl cis-trans isomerase |
| 204 | c22399_g1 | 0 | 1 | 62 | 103 | 19 | 0 | 0.072 | 4.418 | 6.081 | 0.897 | 6.529459 | 0.000683 | 0.015873 | peroxidase 12-like |
| 205 | c32159_g1 | 1 | 0 | 260 | 672 | 149 | 0.071 | 0 | 18.704 | 41.392 | 7.924 | 9.066433 | 5.14E-09 | 4.85E-07 | Peroxidase 72 precursor |
| 206 | c21949_g1 | 0 | 0 | 12 | 37 | 27 | 0 | 0 | 0.705 | 1.796 | 1.043 | 7.579761 | 0.00375 | 0.062547 | PHD finger protein MALE STERILITY 1 |
| 207 | c38136_g3 | 1 | 1 | 15 | 14 | 12 | 0.1 | 0.114 | 1.704 | 1.317 | 0.897 | 3.485722 | 0.059864 | 0.421323 | Phosphate transporter PHO1 homolog 3 |
| 208 | c35122_g1 | 0 | 0 | 82 | 41 | 17 | 0 | 0 | 13.381 | 5.402 | 1.722 | 8.608713 | 0.000592 | 0.014147 | phospholipase A2 homolog 3 |
| 209 | c49463_g3 | 0 | 1 | 532 | 227 | 146 | 0 | 0.083 | 58.805 | 20.221 | 9.104 | 8.872886 | 1.50E-08 | 1.30E-06 | pistil-specific extensin-like protein |
| 210 | c48598_g2 | 0 | 1 | 68 | 60 | 40 | 0 | 0.186 | 6.543 | 5.714 | 2.903 | 6.383666 | 0.000474 | 0.011776 | Pleiotropic drug resistance protein 3 |
| 211 | c39630_g1 | 0 | 1 | 129 | 118 | 33 | 0 | 0.29 | 10.551 | 9.384 | 1.69 | 7.160088 | 7.14E-05 | 0.002365 | Pollen-specific protein-like At4g18596 |
| 212 | c43461_g1 | 0 | 0 | 528 | 486 | 164 | 0 | 0 | 58.405 | 44.329 | 12.169 | 11.64551 | 1.39E-10 | 1.77E-08 | polygalacturonase At1g48100-like |
| 213 | c51808_g2 | 0 | 0 | 54 | 176 | 13 | 0 | 0 | 9.173 | 24.954 | 1.463 | 9.337202 | 0.000181 | 0.005212 | polygalacturonase inhibiting protein |
| 214 | c28315_g1 | 0 | 0 | 14 | 18 | 171 | 0 | 0 | 0.694 | 0.734 | 5.595 | 8.844832 | 0.000691 | 0.016026 | polyphenol oxidase precursor |
| 215 | c42160_g1 | 0 | 1 | 297 | 930 | 389 | 0 | 0.031 | 16.316 | 28.345 | 8.425 | 9.598248 | 1.27E-11 | 1.94E-09 | potassium transporter 8 |
| 216 | c42237_g1 | 1 | 1 | 249 | 115 | 32 | 0.199 | 0.103 | 46.434 | 11.38 | 3.121 | 6.855223 | 4.35E-05 | 0.001547 | protein ABIL5 |
| 217 | c38334_g1 | 1 | 0 | 130 | 70 | 42 | 0.028 | 0 | 4.134 | 1.843 | 0.881 | 6.969895 | 0.000115 | 0.003544 | Protein argonaute 7 |
| 218 | c36862_g1 | 0 | 0 | 24 | 55 | 52 | 0 | 0 | 1.01 | 1.915 | 1.447 | 8.357827 | 0.000493 | 0.012166 | Protein ECERIFERUM 1 |
| 219 | c43678_g1 | 0 | 0 | 16 | 82 | 19 | 0 | 0 | 1.22 | 5.179 | 0.954 | 8.239827 | 0.001551 | 0.031144 | protein FAM179B-like |
| 220 | c33084_g1 | 0 | 0 | 12 | 39 | 14 | 0 | 0 | 0.926 | 2.506 | 0.72 | 7.394305 | 0.006473 | 0.095157 | protein FD-like |
| 221 | c38256_g3 | 1 | 0 | 26 | 23 | 27 | 0.128 | 0 | 3.787 | 2.793 | 2.604 | 5.218629 | 0.008612 | 0.116558 | protein GLUTAMINE DUMPER |
| 222 | c48982_g1 | 0 | 0 | 122 | 336 | 49 | 0 | 0 | 5.123 | 11.747 | 1.35 | 10.3916 | 1.29E-06 | 7.18E-05 | protein IQ-DOMAIN 1-like |
| 223 | c41332_g2 | 0 | 0 | 122 | 168 | 246 | 0 | 0 | 8.784 | 9.927 | 11.716 | 10.38244 | 3.89E-08 | 3.09E-06 | protein phosphatase 2C 75 |
| 224 | c44549_g1 | 0 | 0 | 32 | 33 | 31 | 0 | 0 | 3.335 | 2.857 | 2.135 | 7.963624 | 0.001259 | 0.026308 | protein S-acyltransferase 8-like |
| 225 | c37181_g1 | 0 | 1 | 88 | 173 | 70 | 0 | 0.041 | 3.829 | 6.272 | 2.021 | 7.336013 | 1.47E-05 | 0.000605 | protein SHI RELATED SEQUENCE |
| 226 | c31833_g1 | 0 | 0 | 72 | 53 | 83 | 0 | 0 | 7.921 | 4.916 | 6.177 | 9.061532 | 3.88E-05 | 0.001401 | protein SHI RELATED SEQUENCE |
| 227 | c39301_g2 | 0 | 1 | 118 | 88 | 19 | 0 | 0.052 | 6.459 | 3.99 | 0.687 | 6.865679 | 0.000332 | 0.008736 | protein SRG1-like |
| 228 | c52270_g1 | 0 | 0 | 88 | 179 | 73 | 0 | 0 | 4.124 | 6.935 | 2.256 | 9.792302 | 2.85E-06 | 0.000146 | STRUBBELIG-RECEPTOR FAMILY |
| 229 | c34743_g1 | 0 | 0 | 32 | 36 | 21 | 0 | 0 | 3.345 | 3.112 | 1.455 | 7.880634 | 0.001822 | 0.035445 | protein terminal ear1 homolog |
| 230 | c44215_g1 | 0 | 0 | 66 | 193 | 31 | 0 | 0 | 1.841 | 4.445 | 0.574 | 9.580884 | 3.03E-05 | 0.001138 | protein timeless homolog |
| 231 | c37955_g1 | 0 | 0 | 191 | 325 | 114 | 0 | 0 | 11.898 | 18.242 | 5.037 | 10.69979 | 2.36E-08 | 1.95E-06 | protein trichome birefringence-like 36 |
| 232 | c37563_g1 | 0 | 1 | 12 | 82 | 21 | 0 | 0.072 | 0.936 | 5.291 | 1.083 | 5.779776 | 0.005416 | 0.083201 | Protein trichome birefringence-like 42 |
| 233 | c44240_g2 | 0 | 0 | 163 | 359 | 17 | 0 | 0 | 12.487 | 22.815 | 0.865 | 10.5091 | 6.71E-06 | 0.000305 | protein UPSTREAM OF FLC-like |
| 234 | c23826_g1 | 0 | 1 | 75 | 38 | 70 | 0 | 0.083 | 6.301 | 2.649 | 3.889 | 6.474943 | 0.000339 | 0.008884 | protein with unknown function |
| 235 | c34961_g1 | 0 | 0 | 49 | 198 | 30 | 0 | 0 | 4.839 | 16.112 | 1.965 | 9.50267 | 5.19E-05 | 0.001795 | protein with unknown function |
| 236 | c34526_g1 | 1 | 0 | 42 | 66 | 35 | 0.071 | 0 | 3.545 | 4.628 | 1.957 | 6.141038 | 0.001018 | 0.022144 | Protein XRI1 |
| 237 | c38639_g1 | 1 | 1 | 54 | 54 | 33 | 0.1 | 0.114 | 5.07 | 4.541 | 2.135 | 5.277404 | 0.001714 | 0.033752 | protein XRI1-like |
| 238 | c45067_g1 | 0 | 1 | 45 | 176 | 89 | 0 | 0.041 | 1.778 | 5.778 | 2.321 | 7.194573 | 3.59E-05 | 0.001317 | pto-interacting protein 1 |
| 239 | c2229_g1 | 0 | 0 | 36 | 32 | 11 | 0 | 0 | 4.523 | 3.407 | 1.051 | 7.752371 | 0.003416 | 0.058084 | putative polyprotein |
| 240 | c44499_g1 | 0 | 0 | 60 | 26 | 133 | 0 | 0 | 5.27 | 2.338 | 6.048 | 9.067889 | 8.81E-05 | 0.002822 | pyruvate kinase |
| 241 | c13201_g1 | 0 | 0 | 21 | 16 | 18 | 0 | 0 | 1.525 | 0.966 | 0.865 | 7.173727 | 0.007353 | 0.10414 | r2r3-myb transcription factor |
| 242 | c29237_g1 | 0 | 0 | 217 | 179 | 144 | 0 | 0 | 12.834 | 8.77 | 5.636 | 10.48058 | 3.42E-08 | 2.75E-06 | R2R3-MYB transcription factor |
| 243 | c42933_g1 | 0 | 0 | 19 | 281 | 421 | 0 | 0 | 0.936 | 11.675 | 14.004 | 10.72673 | 1.29E-06 | 7.14E-05 | ras-GTPase-activating protein |
| 244 | c38532_g1 | 0 | 0 | 11 | 187 | 17 | 0 | 0 | 1.147 | 16.136 | 1.164 | 9.114921 | 0.000729 | 0.016764 | Receptor-like protein kinase 2 |
| 245 | c29704_g1 | 0 | 1 | 22 | 63 | 43 | 0 | 0.103 | 2.22 | 5.275 | 2.862 | 5.914577 | 0.001957 | 0.037552 | receptor-like protein kinase |
| 246 | c22953_g1 | 1 | 1 | 22 | 38 | 17 | 0.1 | 0.114 | 2.504 | 3.591 | 1.277 | 4.388151 | 0.014746 | 0.170186 | receptor-like serine/threonine-protein kinase |
| 247 | c43724_g1 | 0 | 0 | 60 | 84 | 86 | 0 | 0 | 5.365 | 6.232 | 5.191 | 9.19202 | 2.03E-05 | 0.0008 | Remorin |
| 248 | c48729_g1 | 0 | 0 | 121 | 360 | 96 | 0 | 0 | 6.491 | 15.681 | 2.466 | 10.55456 | 1.49E-07 | 1.04E-05 | reticulon-like protein B21-like |
| 249 | c32681_g2 | 0 | 0 | 76 | 388 | 13 | 0 | 0 | 8.573 | 36.397 | 0.97 | 10.30088 | 3.32E-05 | 0.001229 | Rho GDP-dissociation inhibitor 1 |
| 250 | c39659_g1 | 0 | 0 | 54 | 267 | 32 | 0 | 0 | 5.439 | 24.874 | 2.66 | 9.850603 | 2.40E-05 | 0.000928 | ribonucleoside-diphosphate reductase |
| 251 | c30254_g1 | 0 | 1 | 109 | 208 | 42 | 0 | 0.041 | 5.049 | 7.972 | 1.286 | 7.48332 | 2.14E-05 | 0.000838 | Rop guanine nucleotide exchange factor 1 |
| 252 | c27796_g1 | 0 | 0 | 22 | 95 | 24 | 0 | 0 | 1.536 | 5.482 | 1.108 | 8.510905 | 0.000711 | 0.016404 | salicylate O-methyltransferase-like |
| 253 | c40512_g1 | 1 | 0 | 195 | 624 | 89 | 0.043 | 0 | 9.405 | 24.922 | 2.838 | 8.818851 | 1.02E-07 | 7.42E-06 | Salutaridinol 7-O-acetyltransferase |
| 254 | c45922_g6 | 0 | 0 | 33 | 32 | 26 | 0 | 0 | 14.696 | 12.09 | 7.673 | 7.901958 | 0.00156 | 0.031255 | Serine carboxypeptidase-like 18 |
| 255 | c45064_g1 | 0 | 0 | 187 | 121 | 24 | 0 | 0 | 10.288 | 5.514 | 0.873 | 9.859497 | 1.77E-05 | 0.000711 | serine incorporator 3-like |
| 256 | c36518_g1 | 1 | 0 | 45 | 46 | 66 | 0.057 | 0 | 6.122 | 2.25 | 9.04 | 6.232289 | 0.000621 | 0.014706 | serine/threonine-protein kinase |
| 257 | c52874_g3 | 1 | 1 | 50 | 70 | 100 | 0.456 | 0.507 | 23.154 | 27.196 | 31.517 | 5.829592 | 0.000215 | 0.006046 | serine/threonine-protein kinase |
| 258 | c35658_g1 | 0 | 0 | 48 | 143 | 21 | 0 | 0 | 3.566 | 8.81 | 1.035 | 9.131001 | 0.000156 | 0.00456 | serine/threonine-protein phosphatase |
| 259 | c40998_g1 | 1 | 0 | 1091 | 1455 | 136 | 0.214 | 0 | 249.893 | 279.356 | 20.561 | 10.43578 | 1.50E-10 | 1.91E-08 | snakin-2 isoform 1 |
| 260 | c38246_g1 | 0 | 0 | 11 | 26 | 40 | 0 | 0 | 2.262 | 3.727 | 5.571 | 7.5555 | 0.00391 | 0.064679 | Sodium/hydrogen exchanger 4 |
| 261 | c40695_g1 | 0 | 0 | 41 | 38 | 28 | 0 | 0 | 4.608 | 3.551 | 2.086 | 8.145062 | 0.000902 | 0.019997 | sodium/hydrogen exchanger 4-like |
| 262 | c48058_g2 | 0 | 0 | 92 | 87 | 28 | 0 | 0 | 17.967 | 14.204 | 3.622 | 9.137884 | 7.78E-05 | 0.002541 | squamosa promoter-binding-like protein |
| 263 | c25108_g1 | 1 | 0 | 140 | 210 | 346 | 0.057 | 0 | 8.016 | 10.023 | 13.09 | 8.335302 | 2.07E-08 | 1.73E-06 | squamosa promoter-binding-like protein |
| 264 | c43260_g1 | 0 | 0 | 88 | 166 | 95 | 0 | 0 | 12.518 | 18.37 | 7.988 | 9.814865 | 1.62E-06 | 8.75E-05 | SQUINT family protein |
| 265 | c38109_g1 | 1 | 0 | 25 | 111 | 11 | 0.043 | 0 | 1.105 | 4.07 | 0.323 | 6.192961 | 0.0032 | 0.055208 | Structural maintenance of chromosome |
| 266 | c32612_g1 | 0 | 0 | 45 | 98 | 33 | 0 | 0 | 5.902 | 10.701 | 2.862 | 8.849228 | 0.000157 | 0.004603 | subtilase family protein |
| 267 | c38017_g2 | 0 | 0 | 70 | 132 | 28 | 0 | 0 | 27.141 | 43.3 | 7.196 | 9.26108 | 6.18E-05 | 0.002081 | subtilisin-like protease |
| 268 | c36764_g1 | 1 | 1 | 90 | 14 | 20 | 0.171 | 0.197 | 17.305 | 2.25 | 2.547 | 5.184591 | 0.006269 | 0.092976 | sucrose synthase 2 |
| 269 | c33311_g1 | 0 | 0 | 62 | 11 | 63 | 0 | 0 | 9.531 | 1.412 | 6.404 | 8.460647 | 0.000835 | 0.018743 | sulfotransferase 16-like |
| 270 | c55262_g1 | 0 | 0 | 28 | 84 | 15 | 0 | 0 | 2.178 | 5.426 | 0.776 | 8.387444 | 0.001072 | 0.023079 | syntaxin-related protein KNOLLE |
| 271 | c51159_g2 | 0 | 0 | 70 | 53 | 164 | 0 | 0 | 6.743 | 4.237 | 10.446 | 9.458695 | 1.12E-05 | 0.000477 | TAGL12 transcription factor |
| 272 | c13344_g1 | 0 | 0 | 12 | 23 | 18 | 0 | 0 | 3.503 | 5.65 | 3.485 | 7.082756 | 0.008503 | 0.115358 | TED4 |
| 273 | c39474_g1 | 0 | 0 | 112 | 681 | 20 | 0 | 0 | 11.267 | 65.061 | 1.286 | 11.06533 | 3.74E-06 | 0.000184 | terminal flower 1 |
| 274 | c32304_g1 | 0 | 0 | 55 | 82 | 25 | 0 | 0 | 24.206 | 30.611 | 7.293 | 8.756709 | 0.000232 | 0.006439 | thaumatin-like protein |
| 275 | c44149_g1 | 0 | 0 | 253 | 821 | 101 | 0 | 0 | 12.687 | 34.075 | 3.501 | 11.60036 | 5.67E-09 | 5.31E-07 | thaumatin-like protein 1-like |
| 276 | c40645_g1 | 0 | 1 | 131 | 348 | 264 | 0 | 0.414 | 18.157 | 41.879 | 28.097 | 8.44751 | 1.42E-08 | 1.23E-06 | thioredoxin h |
| 277 | c13352_g1 | 0 | 0 | 68 | 136 | 25 | 0 | 0 | 8.637 | 11.14 | 1.633 | 9.255995 | 7.48E-05 | 0.00246 | transcription factor |
| 278 | c31332_g1 | 0 | 0 | 54 | 73 | 18 | 0 | 0 | 3.061 | 3.431 | 0.671 | 8.611354 | 0.000458 | 0.011447 | Transcription factor bHLH30 |
| 279 | c43623_g1 | 0 | 0 | 160 | 261 | 114 | 0 | 0 | 10.299 | 14.372 | 5.053 | 10.45565 | 6.76E-08 | 5.11E-06 | transcription factor bHLH93 |
| 280 | c14813_g1 | 0 | 0 | 43 | 51 | 48 | 0 | 0 | 9.878 | 9.823 | 7.301 | 8.516131 | 0.000266 | 0.007231 | transcription factor bHLH94-like |
| 281 | c27975_g1 | 1 | 1 | 32 | 58 | 50 | 0.071 | 0.072 | 3.24 | 4.708 | 4.77 | 5.202769 | 0.001829 | 0.035544 | transcription factor DcMYB2 |
| 282 | c58447_g1 | 0 | 0 | 11 | 26 | 20 | 0 | 0 | 0.926 | 1.803 | 1.108 | 7.176664 | 0.007661 | 0.107247 | transcription factor HEC1 |
| 283 | c22026_g1 | 1 | 1 | 12 | 18 | 12 | 0.214 | 0.238 | 2.809 | 3.535 | 1.86 | 3.504241 | 0.059958 | 0.421806 | transcription factor KAN2 |
| 284 | c41751_g2 | 0 | 0 | 157 | 37 | 400 | 0 | 0 | 11.898 | 2.322 | 20.626 | 10.48613 | 9.40E-07 | 5.40E-05 | Transcription factor MYB108 |
| 285 | c31153_g1 | 1 | 0 | 41 | 22 | 18 | 0.157 | 0 | 7.027 | 3.152 | 2.046 | 5.377375 | 0.007962 | 0.110296 | transcription factor RF2b |
| 286 | c35142_g2 | 1 | 0 | 85 | 108 | 38 | 0.071 | 0 | 6.217 | 6.464 | 1.811 | 6.866664 | 0.000133 | 0.004009 | transcription factor TGA1-like |
| 287 | c3525_g1 | 0 | 1 | 11 | 31 | 13 | 0 | 0.135 | 1.494 | 3.495 | 1.164 | 4.730235 | 0.024486 | 0.241303 | transmembrane 9 superfamily member |
| 288 | c21551_g1 | 0 | 0 | 86 | 260 | 88 | 0 | 0 | 16.369 | 41.36 | 11.085 | 10.13266 | 9.28E-07 | 5.34E-05 | triacylglycerol lipase |
| 289 | c50312_g1 | 0 | 0 | 89.85 | 134.01 | 195.59 | 0 | 0 | 7.448 | 7.892 | 8.393 | 10.02578 | 3.52E-07 | 2.24E-05 | Two-pore potassium channel 1 |
| 290 | c33787_g1 | 0 | 1 | 37 | 159 | 22 | 0 | 0.062 | 2.367 | 8.443 | 0.93 | 6.735695 | 0.000593 | 0.014163 | U-box domain-containing protein 30 |
| 291 | c36643_g1 | 0 | 0 | 28 | 13 | 53 | 0 | 0 | 4.429 | 1.716 | 5.547 | 7.868298 | 0.002217 | 0.041516 | uclacyanin-3-like |
| 292 | c38116_g1 | 0 | 1 | 284 | 375 | 137 | 0 | 0.466 | 34.788 | 37.482 | 13.357 | 8.633053 | 1.19E-08 | 1.04E-06 | Uncharacterized protein |
| 293 | c36613_g1 | 0 | 0 | 49 | 89 | 39 | 0 | 0 | 5.617 | 8.491 | 2.967 | 8.854667 | 0.000121 | 0.003684 | Uncharacterized protein |
| 294 | c24044_g1 | 0 | 0 | 48 | 48 | 39 | 0 | 0 | 12.718 | 10.685 | 6.848 | 8.467018 | 0.000342 | 0.008949 | Uncharacterized protein |
| 295 | c41924_g2 | 0 | 0 | 41 | 61.57 | 28 | 0 | 0 | 6.533 | 8.18 | 2.951 | 8.431242 | 0.000472 | 0.01174 | Uncharacterized protein |
| 296 | c59907_g1 | 1 | 1 | 39 | 65 | 44 | 0.214 | 0.238 | 9.068 | 12.672 | 6.776 | 5.305033 | 0.001396 | 0.028601 | Uncharacterized protein |
| 297 | c76850_g1 | 0 | 0 | 34 | 52 | 19 | 0 | 0 | 3.156 | 3.998 | 1.164 | 8.122854 | 0.00123 | 0.025821 | Uncharacterized protein |
| 298 | c11505_g1 | 0 | 0 | 26 | 16 | 15 | 0 | 0 | 3.114 | 1.596 | 1.189 | 7.255827 | 0.006815 | 0.098518 | Uncharacterized protein |
| 299 | c34065_g1 | 1 | 0 | 26 | 24 | 83 | 0.142 | 0 | 4.229 | 3.256 | 8.942 | 5.922665 | 0.00227 | 0.042292 | Uncharacterized protein |
| 300 | c53829_g3 | 0 | 0 | 25 | 16 | 17 | 0 | 0 | 7.627 | 4.11 | 3.436 | 7.268568 | 0.006446 | 0.094843 | Uncharacterized protein |
| 301 | c34835_g1 | 1 | 1 | 23 | 25 | 24 | 0.085 | 0.103 | 2.335 | 2.107 | 1.609 | 4.273673 | 0.016047 | 0.180628 | Uncharacterized protein |
| 302 | c45480_g1 | 0 | 1 | 23 | 174 | 103 | 0 | 0.217 | 3.387 | 24.634 | 11.126 | 7.116258 | 0.000102 | 0.003194 | Uncharacterized protein |
| 303 | c44694_g2 | 0 | 0 | 18 | 15 | 12 | 0 | 0 | 5.197 | 3.423 | 2.159 | 6.903575 | 0.012101 | 0.148486 | Uncharacterized protein |
| 304 | c33771_g1 | 0 | 0 | 40 | 72 | 56 | 0 | 0 | 3.798 | 5.754 | 3.582 | 8.74398 | 0.000131 | 0.00396 | uncharacterized protein At3g17950 |
| 305 | c38857_g1 | 0 | 0 | 115 | 110 | 81 | 0 | 0 | 10.604 | 8.427 | 4.94 | 9.656076 | 3.30E-06 | 0.000165 | Uncharacterized protein At3g27210 |
| 306 | c48150_g2 | 0 | 1 | 39 | 147 | 19 | 0 | 0.114 | 4.408 | 13.813 | 1.423 | 6.653708 | 0.000744 | 0.017056 | uncharacterized protein At5g01610 |
| 307 | c53435_g7 | 0 | 1 | 19 | 12 | 24 | 0 | 0.59 | 10.814 | 5.833 | 9.064 | 4.71776 | 0.022099 | 0.225161 | uncharacterized protein LOC100240792 |
| 308 | c33480_g1 | 0 | 0 | 188 | 250 | 21 | 0 | 0 | 47.601 | 52.868 | 3.347 | 10.29843 | 6.38E-06 | 0.000293 | uncharacterized protein LOC100241009 |
| 309 | c30014_g1 | 0 | 0 | 33 | 26 | 47 | 0 | 0 | 4.271 | 2.801 | 4.018 | 8.072848 | 0.000993 | 0.021699 | uncharacterized protein LOC100243168 |
| 310 | c3479_g1 | 0 | 0 | 78 | 45 | 52 | 0 | 0 | 5.449 | 2.609 | 2.401 | 8.859515 | 0.000107 | 0.003329 | uncharacterized protein LOC100243219 |
| 311 | c13138_g1 | 1 | 0 | 12 | 28 | 15 | 0.1 | 0 | 1.378 | 2.681 | 1.14 | 4.746049 | 0.023325 | 0.233916 | uncharacterized protein LOC100244201 |
| 312 | c51360_g2 | 0 | 0 | 26 | 37 | 24 | 0 | 0 | 3.692 | 4.373 | 2.256 | 7.825061 | 0.001924 | 0.037066 | uncharacterized protein LOC100244412 |
| 313 | c15506_g1 | 0 | 0 | 22 | 50 | 24 | 0 | 0 | 2.241 | 4.182 | 1.633 | 7.956491 | 0.001613 | 0.03218 | uncharacterized protein LOC100244711 |
| 314 | c30791_g1 | 0 | 0 | 12 | 26 | 12 | 0 | 0 | 1.02 | 1.843 | 0.687 | 7.02512 | 0.010475 | 0.134318 | uncharacterized protein LOC100245492 |
| 315 | c35296_g1 | 0 | 1 | 54 | 54 | 21 | 0 | 0.062 | 3.272 | 2.705 | 0.841 | 6.022647 | 0.001863 | 0.036075 | uncharacterized protein LOC100245695 |
| 316 | c27311_g1 | 0 | 1 | 308 | 447 | 351 | 0 | 0.072 | 22.891 | 28.177 | 17.028 | 9.055949 | 4.32E-11 | 5.95E-09 | uncharacterized protein LOC100245764 |
| 317 | c49901_g1 | 0 | 0 | 73 | 105 | 69 | 0 | 0 | 2.809 | 3.304 | 1.625 | 9.32528 | 1.46E-05 | 0.0006 | uncharacterized protein LOC100248222 |
| 318 | c50857_g1 | 1 | 0 | 455 | 442 | 73 | 0.057 | 0 | 31.38 | 24.985 | 2.886 | 8.97756 | 5.31E-08 | 4.11E-06 | uncharacterized protein LOC100250572 |
| 319 | c34163_g1 | 0 | 0 | 17 | 21 | 16 | 0 | 0 | 1.915 | 1.971 | 1.197 | 7.139403 | 0.007586 | 0.106455 | uncharacterized protein LOC100252554 |
| 320 | c38244_g2 | 0 | 0 | 56 | 156 | 63 | 0 | 0 | 4.818 | 11.14 | 3.582 | 9.470164 | 1.44E-05 | 0.000596 | uncharacterized protein LOC100253812 |
| 321 | c38446_g1 | 1 | 0 | 75 | 70 | 82 | 0.028 | 0 | 2.746 | 2.123 | 1.989 | 6.786765 | 8.28E-05 | 0.002674 | uncharacterized protein LOC100254987 |
| 322 | c13219_g1 | 0 | 1 | 12 | 27 | 15 | 0 | 0.103 | 1.315 | 2.458 | 1.092 | 4.699694 | 0.023288 | 0.233858 | uncharacterized protein LOC100257562 |
| 323 | c45180_g1 | 0 | 0 | 170 | 300 | 17 | 0 | 0 | 31.696 | 47.154 | 2.409 | 10.37264 | 8.45E-06 | 0.000374 | uncharacterized protein LOC100257934 |
| 324 | c46398_g3 | 1 | 0 | 59 | 272 | 24 | 0.057 | 0 | 3.692 | 14.109 | 0.995 | 7.462511 | 0.000113 | 0.003494 | uncharacterized protein LOC100262872 |
| 325 | c33894_g1 | 0 | 0 | 14 | 71 | 25 | 0 | 0 | 0.778 | 3.256 | 0.962 | 8.133679 | 0.001647 | 0.03271 | uncharacterized protein LOC100263016 |
| 326 | c33967_g1 | 0 | 0 | 90 | 243 | 45 | 0 | 0 | 4.587 | 10.167 | 1.52 | 9.962489 | 5.18E-06 | 0.000243 | uncharacterized protein LOC100267160 |
| 327 | c51212_g1 | 1 | 0 | 54 | 124 | 115 | 0.043 | 0 | 3.861 | 6.089 | 3.582 | 7.111636 | 2.70E-05 | 0.001028 | uncharacterized protein LOC100268097 |
| 328 | c36426_g1 | 0 | 0 | 23 | 44 | 56 | 0 | 0 | 3.503 | 6.169 | 5.935 | 8.254102 | 0.000627 | 0.014811 | uncharacterized protein LOC100305838 |
| 329 | c44491_g1 | 0 | 0 | 178 | 551 | 170 | 0 | 0 | 4.197 | 10.781 | 2.604 | 11.18588 | 2.08E-09 | 2.13E-07 | uncharacterized protein LOC100381735 |
| 330 | c44221_g1 | 1 | 1 | 610 | 515 | 522 | 0.085 | 0.052 | 37.839 | 25.967 | 24.838 | 8.799021 | 4.74E-13 | 9.09E-11 | uncharacterized protein LOC101218342 |
| 331 | c32179_g1 | 0 | 1 | 14 | 26 | 11 | 0 | 0.217 | 2.977 | 4.628 | 1.552 | 4.643472 | 0.026639 | 0.255625 | uncharacterized protein LOC101229117 |
| 332 | c7231_g1 | 0 | 1 | 59 | 38 | 20 | 0 | 0.083 | 5.218 | 2.785 | 1.172 | 5.899091 | 0.002664 | 0.047831 | uncharacterized protein LOC101245645 |
| 333 | c37044_g1 | 1 | 0 | 46 | 80 | 11 | 0.043 | 0 | 1.894 | 2.729 | 0.299 | 6.127615 | 0.002651 | 0.047702 | uncharacterized protein LOC101246082 |
| 334 | c50949_g2 | 0 | 0 | 70 | 147 | 45 | 0 | 0 | 3.482 | 6.081 | 1.447 | 9.428689 | 2.12E-05 | 0.000833 | uncharacterized protein LOC101246157 |
| 335 | c18606_g1 | 0 | 1 | 57 | 59 | 34 | 0 | 0.124 | 7.132 | 6.145 | 2.814 | 6.217169 | 0.000815 | 0.018383 | uncharacterized protein LOC101251810 |
| 336 | c35462_g1 | 0 | 0 | 15 | 49 | 28 | 0 | 0 | 1.757 | 4.788 | 2.175 | 7.867214 | 0.002094 | 0.039635 | uncharacterized protein LOC101251810 |
| 337 | c42602_g1 | 1 | 0 | 77 | 136 | 34 | 0.185 | 0 | 11.214 | 9.217 | 4.633 | 6.956589 | 0.000129 | 0.003902 | uncharacterized protein LOC101253506 |
| 338 | c23190_g1 | 0 | 0 | 24 | 30 | 16 | 0 | 0 | 1.82 | 1.891 | 0.809 | 7.533438 | 0.003934 | 0.065034 | uncharacterized protein LOC101254679 |
| 339 | c27116_g1 | 1 | 0 | 11 | 18 | 16 | 0.242 | 0 | 2.893 | 3.974 | 2.789 | 4.44581 | 0.032725 | 0.291766 | uncharacterized protein LOC101255523 |
| 340 | c42733_g1 | 0 | 0 | 303 | 521 | 17 | 0 | 0 | 71.228 | 106.932 | 2.482 | 11.16648 | 1.21E-06 | 6.78E-05 | uncharacterized protein LOC101257656 |
| 341 | c30089_g1 | 1 | 0 | 19 | 46 | 30 | 0.128 | 0 | 2.262 | 4.533 | 2.329 | 5.51525 | 0.005009 | 0.078475 | uncharacterized protein LOC101259876 |
| 342 | c49414_g4 | 0 | 1 | 57 | 98 | 43 | 0 | 0.062 | 3.882 | 5.538 | 1.941 | 6.598499 | 0.000248 | 0.006811 | uncharacterized protein LOC101262169 |
| 343 | c37405_g1 | 0 | 0 | 52 | 97 | 63 | 0 | 0 | 3.756 | 5.674 | 3.008 | 9.089153 | 3.96E-05 | 0.001427 | uncharacterized protein LOC101264968 |
| 344 | c45851_g2 | 0 | 0 | 17 | 45 | 20 | 0 | 0 | 0.968 | 2.123 | 5.256 | 7.726399 | 0.002979 | 0.051984 | uncharacterized protein LOC101264968 |
| 345 | c32390_g1 | 1 | 0 | 138 | 127 | 130 | 0.1 | 0 | 14.759 | 11.292 | 9.201 | 7.596034 | 1.86E-06 | 9.96E-05 | uncharacterized protein LOC101303530 |
| 346 | c16878_g1 | 1 | 0 | 94 | 85 | 116 | 0.769 | 0 | 78.224 | 60.951 | 64.134 | 7.153445 | 1.69E-05 | 0.000685 | uncharacterized protein LOC101308840 |
| 347 | c51995_g3 | 1 | 0 | 29 | 67 | 13 | 0.157 | 0 | 4.829 | 9.305 | 1.431 | 5.774002 | 0.004692 | 0.074459 | uncharacterized protein LOC101311909 |
| 348 | c15750_g1 | 0 | 1 | 192 | 525 | 21 | 0 | 0.124 | 23.869 | 54.32 | 1.73 | 8.532307 | 6.81E-06 | 0.000309 | unknown |
| 349 | c7181_g1 | 0 | 0 | 12 | 14 | 14 | 0 | 0 | 1.915 | 1.859 | 1.48 | 6.694322 | 0.015195 | 0.173579 | unknown |
| 350 | c18604_g1 | 0 | 0 | 1140 | 988 | 548 | 0 | 0 | 125.93 | 90.733 | 40.039 | 12.80917 | 3.56E-16 | 1.14E-13 | unkown |
| 351 | c24818_g1 | 1 | 0 | 1126 | 2285 | 270 | 0.555 | 0 | 681.723 | 1182.2 | 108.458 | 10.86372 | 1.93E-12 | 3.33E-10 | unkown |
| 352 | c24601_g1 | 1 | 0 | 529 | 315 | 210 | 0.128 | 0 | 74.931 | 37.179 | 19.68 | 9.075762 | 7.89E-10 | 8.69E-08 | unkown |
| 353 | c38908_g1 | 1 | 1 | 500 | 337 | 317 | 0.456 | 0.507 | 245.917 | 140.943 | 103.388 | 8.310484 | 1.24E-10 | 1.60E-08 | unkown |
| 354 | c31606_g1 | 1 | 0 | 390 | 560 | 706 | 0.256 | 0 | 107.153 | 127.353 | 128.485 | 9.609882 | 1.78E-13 | 3.70E-11 | unkown |
| 355 | c29916_g1 | 0 | 0 | 185 | 53 | 61 | 0 | 0 | 398.335 | 101.219 | 87.986 | 9.691682 | 1.40E-05 | 0.000581 | unkown |
| 356 | c50827_g7 | 0 | 0 | 165 | 81 | 34 | 0 | 0 | 34.262 | 16.391 | 6.557 | 9.608693 | 2.34E-05 | 0.000909 | unkown |
| 357 | c43220_g4 | 0 | 0 | 158 | 22 | 216 | 0 | 0 | 66.421 | 7.836 | 60.196 | 9.966958 | 9.37E-06 | 0.000408 | unkown |
| 358 | c32955_g2 | 1 | 0 | 155 | 554 | 172 | 0.043 | 0 | 6.427 | 19.344 | 4.884 | 8.742621 | 1.81E-08 | 1.54E-06 | unkown |
| 359 | c45901_g4 | 0 | 1 | 136 | 81 | 96 | 0 | 0.259 | 35.683 | 17.899 | 16.729 | 7.272677 | 1.39E-05 | 0.000577 | unkown |
| 360 | c47515_g1 | 0 | 0 | 135 | 153 | 379 | 0 | 0 | 6.901 | 6.472 | 12.799 | 10.66419 | 1.13E-08 | 9.99E-07 | unkown |
| 361 | c41179_g1 | 0 | 0 | 128 | 208 | 164 | 0 | 0 | 18.588 | 25.177 | 15.758 | 10.32079 | 6.18E-08 | 4.73E-06 | unkown |
| 362 | c41970_g1 | 1 | 0 | 119 | 56 | 91 | 0.185 | 0 | 17.263 | 6.663 | 9.46 | 7.047024 | 4.60E-05 | 0.001623 | unkown |
| 363 | c24524_g1 | 1 | 0 | 112 | 63 | 23 | 0.299 | 0 | 35.956 | 17.045 | 4.892 | 6.700366 | 0.000445 | 0.011166 | unkown |
| 364 | c36161_g1 | 0 | 0 | 112 | 208 | 25 | 0 | 0 | 26.983 | 42.031 | 3.986 | 9.861439 | 1.53E-05 | 0.000628 | unkown |
| 365 | c39678_g1 | 1 | 0 | 104 | 20 | 174 | 0.043 | 0 | 5.796 | 0.942 | 6.468 | 7.130622 | 0.000125 | 0.00379 | unkown |
| 366 | c52006_g3 | 0 | 0 | 103 | 71 | 23 | 0 | 0 | 8.342 | 4.636 | 1.229 | 9.088143 | 0.000125 | 0.003798 | unkown |
| 367 | c36039_g2 | 0 | 0 | 91 | 118 | 93 | 0 | 0 | 18.399 | 19.966 | 12.452 | 9.609608 | 3.37E-06 | 0.000168 | unkown |
| 368 | c49127_g1 | 0 | 0 | 90 | 291 | 83 | 0 | 0 | 2.546 | 6.807 | 1.552 | 10.23366 | 8.15E-07 | 4.77E-05 | unkown |
| 369 | c48872_g2 | 0 | 0 | 87 | 110 | 42 | 0 | 0 | 17.399 | 18.402 | 5.563 | 9.317655 | 2.73E-05 | 0.001037 | unkown |
| 370 | c31165_g1 | 0 | 0 | 81 | 489 | 40 | 0 | 0 | 6.312 | 31.601 | 2.062 | 10.6403 | 2.77E-06 | 0.000142 | unkown |
| 371 | c38284_g1 | 0 | 0 | 76 | 18 | 35 | 0 | 0 | 20.208 | 4.022 | 6.161 | 8.458586 | 0.000755 | 0.017281 | unkown |
| 372 | c7086_g1 | 0 | 0 | 68 | 93 | 91 | 0 | 0 | 2.746 | 3.112 | 2.434 | 9.328786 | 1.15E-05 | 0.00049 | unkown |
| 373 | c37233_g2 | 1 | 0 | 66 | 92 | 23 | 0.185 | 0 | 13.76 | 16.048 | 3.178 | 6.523653 | 0.00057 | 0.013676 | unkown |
| 374 | c51671_g5 | 0 | 0 | 66 | 111 | 57 | 0 | 0 | 8.005 | 11.204 | 4.576 | 9.252584 | 2.37E-05 | 0.000917 | unkown |
| 375 | c53036_g4 | 0 | 0 | 65 | 31 | 20 | 0 | 0 | 21.386 | 8.419 | 4.002 | 8.320988 | 0.000953 | 0.020966 | unkown |
| 376 | c12989_g1 | 0 | 0 | 52 | 20 | 75 | 0 | 0 | 1.988 | 0.71 | 1.989 | 8.537283 | 0.000409 | 0.010421 | unkown |
| 377 | c32612_g2 | 1 | 0 | 50 | 118 | 34 | 0.171 | 0 | 9.415 | 18.577 | 4.237 | 6.645226 | 0.000331 | 0.008716 | unkown |
| 378 | c53456_g4 | 0 | 1 | 48 | 58 | 56 | 0 | 0.548 | 25.605 | 26.358 | 19.809 | 6.281409 | 0.000506 | 0.012412 | unkown |
| 379 | c53122_g6 | 0 | 0 | 47 | 30 | 140 | 0 | 0 | 24.384 | 13.255 | 48.173 | 9.030886 | 9.50E-05 | 0.00301 | unkown |
| 380 | c43607_g1 | 0 | 1 | 47 | 60 | 40 | 0 | 0.373 | 7.522 | 6.28 | 5.215 | 6.163875 | 0.000829 | 0.018647 | unkown |
| 381 | c42348_g1 | 0 | 0 | 45 | 104 | 173 | 0 | 0 | 35.42 | 69.386 | 89.894 | 9.606844 | 6.08E-06 | 0.00028 | unkown |
| 382 | c50657_g2 | 1 | 0 | 43 | 174 | 16 | 0.199 | 0 | 9.668 | 32.782 | 2.385 | 6.86032 | 0.000618 | 0.01465 | unkown |
| 383 | c38761_g1 | 1 | 0 | 41 | 52 | 47 | 0.071 | 0 | 9.152 | 5.522 | 6.274 | 6.089029 | 0.000984 | 0.021543 | unkown |
| 384 | c12454_g1 | 1 | 0 | 40 | 53 | 28 | 0.171 | 0 | 7.595 | 8.411 | 3.517 | 5.912458 | 0.001985 | 0.037948 | unkown |
| 385 | c53580_g2 | 1 | 0 | 39 | 81 | 37 | 0.1 | 0 | 4.513 | 7.788 | 2.83 | 6.267027 | 0.000778 | 0.017705 | unkown |
| 386 | c27994_g1 | 1 | 0 | 39 | 15 | 22 | 0.555 | 0 | 27.93 | 8.267 | 10.123 | 5.272525 | 0.009988 | 0.129899 | unkown |
| 387 | c47034_g1 | 0 | 0 | 39 | 36 | 104 | 0 | 0 | 8.142 | 6.432 | 12.12 | 8.770324 | 0.000157 | 0.004583 | unkown |
| 388 | c33614_g1 | 0 | 1 | 38 | 77 | 140 | 0 | 0.145 | 5.691 | 9.608 | 13.866 | 6.849753 | 9.84E-05 | 0.003107 | unkown |
| 389 | c49428_g5 | 1 | 0 | 37 | 26 | 25 | 0.199 | 0 | 8.268 | 4.868 | 3.703 | 5.463205 | 0.005523 | 0.08437 | unkown |
| 390 | c34428_g1 | 0 | 0 | 36 | 17 | 121 | 0 | 0 | 21.797 | 8.794 | 48.61 | 8.697952 | 0.000417 | 0.010584 | unkown |
| 391 | c71740_g1 | 0 | 0 | 35 | 38 | 33 | 0 | 0 | 4.723 | 4.269 | 2.943 | 8.108057 | 0.000903 | 0.020016 | unkown |
| 392 | c46214_g2 | 1 | 1 | 34 | 26 | 16 | 0.47 | 0.528 | 17.515 | 11.403 | 5.466 | 4.407749 | 0.014728 | 0.170004 | unkown |
| 393 | c30879_g1 | 0 | 0 | 30 | 27 | 12 | 0 | 0 | 4.334 | 4.509 | 1.148 | 7.545439 | 0.004579 | 0.073167 | unkown |
| 394 | c48464_g2 | 0 | 0 | 30 | 25 | 18 | 0 | 0 | 4.702 | 3.264 | 1.868 | 7.604905 | 0.003435 | 0.058318 | unkown |
| 395 | c51202_g3 | 0 | 0 | 29 | 18 | 26 | 0 | 0 | 10.846 | 5.69 | 6.444 | 7.577055 | 0.003384 | 0.057644 | unkown |
| 396 | c67363_g1 | 0 | 0 | 28 | 102 | 36 | 0 | 0 | 1.536 | 4.628 | 1.302 | 8.73783 | 0.000267 | 0.00725 | unkown |
| 397 | c30683_g1 | 1 | 1 | 27 | 73 | 30 | 0.356 | 0.393 | 3.503 | 6.719 | 2.798 | 5.120945 | 0.003113 | 0.053971 | unkown |
| 398 | c22919_g1 | 0 | 0 | 26 | 29 | 49 | 0 | 0 | 29.728 | 29.446 | 37.654 | 8.024337 | 0.001085 | 0.023306 | unkown |
| 399 | c31736_g1 | 1 | 0 | 25 | 11 | 19 | 0.399 | 0 | 10.456 | 4.086 | 5.231 | 4.78271 | 0.022142 | 0.225549 | unkown |
| 400 | c57290_g1 | 0 | 0 | 25 | 14 | 12 | 0 | 0 | 43.004 | 21.203 | 13.794 | 7.109767 | 0.009533 | 0.125395 | unkown |
| 401 | c51831_g5 | 0 | 0 | 24.28 | 18 | 25 | 0 | 0 | 3.524 | 2.155 | 2.506 | 7.441149 | 0.004338 | 0.070226 | unkown |
| 402 | c41763_g1 | 0 | 0 | 23 | 12 | 51 | 0 | 0 | 1.83 | 0.79 | 2.684 | 7.726234 | 0.003171 | 0.054814 | unkown |
| 403 | c36280_g1 | 0 | 0 | 23 | 17 | 13 | 0 | 0 | 3.629 | 4.844 | 1.86 | 7.150451 | 0.00811 | 0.11152 | unkown |
| 404 | c1193_g1 | 0 | 1 | 23 | 15 | 25 | 0 | 0.611 | 13.602 | 7.573 | 9.808 | 4.926214 | 0.015352 | 0.174716 | unkown |
| 405 | c43606_g6 | 1 | 1 | 22 | 16 | 18 | 0.256 | 0.528 | 11.445 | 8.355 | 7.309 | 3.93217 | 0.029986 | 0.277276 | unkown |
| 406 | c45721_g1 | 0 | 0 | 21.46 | 31.21 | 115.78 | 0 | 0 | 2.619 | 3.176 | 9.363 | 8.627899 | 0.000416 | 0.010556 | unkown |
| 407 | c50141_g3 | 0 | 0 | 21 | 57 | 39 | 0 | 0 | 35.451 | 84.692 | 43.985 | 8.209645 | 0.000807 | 0.018248 | unkown |
| 408 | c68543_g1 | 1 | 1 | 21 | 61 | 24 | 0.27 | 0.29 | 6.059 | 14.811 | 4.593 | 4.8262 | 0.006497 | 0.095438 | unkown |
| 409 | c46173_g1 | 1 | 0 | 20 | 28 | 94 | 0.171 | 0 | 9.888 | 10.925 | 31.274 | 5.99347 | 0.002176 | 0.040943 | unkown |
| 410 | c43145_g1 | 0 | 1 | 18 | 81 | 18 | 0 | 0.093 | 1.799 | 9.839 | 1.536 | 5.823114 | 0.004328 | 0.070069 | unkown |
| 411 | c62593_g1 | 1 | 1 | 18 | 52 | 51 | 0.043 | 0.052 | 0.873 | 2.083 | 1.633 | 4.958541 | 0.004068 | 0.066888 | unkown |
| 412 | c66788_g1 | 0 | 0 | 18 | 34 | 15 | 0 | 0 | 4.292 | 6.799 | 2.369 | 7.454924 | 0.004886 | 0.076916 | unkown |
| 413 | c16077_g1 | 0 | 0 | 18 | 34 | 48 | 0 | 0 | 19.63 | 32.191 | 34.832 | 7.949053 | 0.001413 | 0.0289 | unkown |
| 414 | c663_g1 | 0 | 0 | 18 | 14 | 28 | 0 | 0 | 25.962 | 17.684 | 26.941 | 7.247921 | 0.006332 | 0.093587 | unkown |
| 415 | c51254_g4 | 0 | 0 | 18 | 33 | 65 | 0 | 0 | 1.189 | 1.803 | 2.83 | 8.136375 | 0.001039 | 0.022529 | unkown |
| 416 | c68630_g1 | 0 | 0 | 18 | 44 | 17 | 0 | 0 | 2.788 | 5.682 | 1.746 | 7.684209 | 0.003349 | 0.057212 | unkown |
| 417 | c32286_g2 | 1 | 1 | 17 | 13 | 12 | 0.427 | 0.476 | 7.9 | 5.131 | 3.695 | 3.530247 | 0.058225 | 0.415142 | unkown |
| 418 | c48955_g1 | 0 | 0 | 17 | 13 | 11 | 0 | 0 | 12.203 | 8.012 | 5.247 | 6.773019 | 0.014985 | 0.171951 | unkown |
| 419 | c50835_g1 | 0 | 0 | 17 | 28 | 12 | 0 | 0 | 24.521 | 35.367 | 11.546 | 7.232628 | 0.007326 | 0.103801 | unkown |
| 420 | c47563_g3 | 0 | 0 | 17 | 30 | 13 | 0 | 0 | 2.178 | 3.192 | 1.1 | 7.301495 | 0.006549 | 0.09599 | unkown |
| 421 | c42012_g1 | 0 | 0 | 16 | 56 | 70 | 0 | 0 | 0.673 | 1.939 | 1.932 | 8.43303 | 0.000548 | 0.01328 | unkown |
| 422 | c69447_g1 | 0 | 1 | 16 | 16 | 31 | 0 | 0.155 | 2.567 | 2.139 | 3.291 | 4.878391 | 0.016359 | 0.183029 | unkown |
| 423 | c24685_g1 | 0 | 0 | 16 | 12 | 24 | 0 | 0 | 4.713 | 2.969 | 4.681 | 7.045939 | 0.008934 | 0.119885 | unkown |
| 424 | c37416_g2 | 0 | 1 | 14.57 | 57.04 | 66.44 | 0 | 0.103 | 1.504 | 4.892 | 4.665 | 5.972634 | 0.002024 | 0.038555 | unkown |
| 425 | c31648_g1 | 0 | 0 | 14 | 24 | 26 | 0 | 0 | 4.892 | 6.544 | 5.555 | 7.335666 | 0.005136 | 0.079944 | unkown |
| 426 | c10223_g1 | 0 | 0 | 14 | 26 | 17 | 0 | 0 | 7.574 | 11.986 | 6.096 | 7.20086 | 0.007228 | 0.102832 | unkown |
| 427 | c39264_g1 | 0 | 1 | 14 | 17 | 14 | 0 | 0.29 | 6.648 | 5.307 | 2.709 | 4.450955 | 0.03278 | 0.292023 | unkown |
| 428 | c53010_g2 | 1 | 0 | 14 | 42 | 24 | 0.142 | 0 | 2.304 | 5.754 | 2.498 | 5.266737 | 0.009483 | 0.124961 | unkown |
| 429 | c1214_g1 | 0 | 0 | 13 | 20 | 20 | 0 | 0 | 1.515 | 1.931 | 1.536 | 7.078373 | 0.008207 | 0.112475 | unkown |
| 430 | c52966_g2 | 1 | 1 | 13 | 15 | 51 | 0.342 | 0.383 | 4.86 | 4.748 | 12.646 | 4.295712 | 0.019511 | 0.206994 | unkown |
| 431 | c46455_g1 | 1 | 0 | 13 | 12 | 56 | 0.1 | 0 | 1.357 | 1.045 | 3.938 | 5.186528 | 0.013495 | 0.160098 | unkown |
| 432 | c13652_g1 | 0 | 0 | 12 | 13 | 39 | 0 | 0 | 0.873 | 0.782 | 1.876 | 7.279596 | 0.007002 | 0.100543 | unkown |
| 433 | c9163_g1 | 1 | 0 | 12 | 12 | 20 | 0.37 | 0 | 4.776 | 4.046 | 5.28 | 4.397419 | 0.035776 | 0.308762 | unkown |
| 434 | c20084_g1 | 0 | 0 | 12 | 33 | 12 | 0 | 0 | 5.102 | 11.89 | 3.38 | 7.212967 | 0.008491 | 0.115253 | unkown |
| 435 | c15198_g1 | 0 | 1 | 11 | 25 | 11 | 0 | 0.093 | 1.052 | 1.764 | 0.695 | 4.513195 | 0.033495 | 0.296997 | unkown |
| 436 | c46467_g1 | 0 | 0 | 11 | 57 | 28 | 0 | 0 | 8.363 | 37.235 | 14.133 | 7.919848 | 0.00237 | 0.043725 | unkown |
| 437 | c28298_g1 | 1 | 0 | 11 | 17 | 22 | 0.071 | 0 | 0.831 | 1.061 | 1.092 | 4.571328 | 0.027773 | 0.263483 | unkown |
| 438 | c43461_g2 | 0 | 0 | 1023 | 719 | 230 | 0 | 0 | 120.975 | 71.381 | 17.61 | 12.40988 | 3.11E-12 | 5.23E-10 | unnamed protein product |
| 439 | c23368_g1 | 1 | 1 | 664 | 400 | 169 | 0.057 | 0.072 | 45.981 | 22.966 | 7.738 | 8.461337 | 1.90E-09 | 1.95E-07 | unnamed protein product |
| 440 | c42369_g1 | 1 | 0 | 356 | 90 | 48 | 0.114 | 0 | 45.918 | 9.576 | 4.01 | 8.056179 | 1.06E-05 | 0.000453 | unnamed protein product |
| 441 | c31891_g1 | 1 | 0 | 221 | 236 | 203 | 0.171 | 0 | 41.626 | 37.155 | 25.307 | 8.336939 | 1.63E-08 | 1.40E-06 | unnamed protein product |
| 442 | c42979_g4 | 0 | 0 | 177 | 116 | 152 | 0 | 0 | 10.225 | 6.951 | 5.498 | 10.18321 | 1.89E-07 | 1.28E-05 | unnamed protein product |
| 443 | c28028_g1 | 0 | 1 | 154 | 134 | 349 | 0 | 0.083 | 9.92 | 6.847 | 13.648 | 8.194126 | 7.81E-08 | 5.80E-06 | unnamed protein product |
| 444 | c45259_g2 | 1 | 0 | 125 | 317 | 55 | 0.085 | 0 | 7.364 | 16.231 | 2.474 | 7.956047 | 4.11E-06 | 0.000199 | unnamed protein product |
| 445 | c28777_g1 | 0 | 0 | 67 | 23 | 55 | 0 | 0 | 10.982 | 3.144 | 5.967 | 8.573681 | 0.000368 | 0.009505 | unnamed protein product |
| 446 | c78866_g1 | 0 | 0 | 60 | 138 | 12 | 0 | 0 | 10.288 | 19.751 | 1.358 | 9.140573 | 0.000247 | 0.006793 | unnamed protein product |
| 447 | c36792_g1 | 0 | 0 | 49 | 136 | 36 | 0 | 0 | 2.861 | 6.576 | 1.391 | 9.174988 | 6.89E-05 | 0.002291 | unnamed protein product |
| 448 | c21371_g1 | 1 | 0 | 39 | 102 | 42 | 0.128 | 0 | 5.765 | 12.569 | 4.107 | 6.480669 | 0.000443 | 0.011146 | unnamed protein product |
| 449 | c21914_g1 | 0 | 0 | 39 | 65 | 16 | 0 | 0 | 3.356 | 4.948 | 1.043 | 8.326124 | 0.000949 | 0.020871 | unnamed protein product |
| 450 | c15157_g1 | 0 | 1 | 31 | 60 | 51 | 0 | 0.466 | 14.223 | 23.373 | 15.516 | 6.069797 | 0.001062 | 0.022915 | unnamed protein product |
| 451 | c45318_g1 | 0 | 0 | 18 | 14 | 13 | 0 | 0 | 8.174 | 4.365 | 4.099 | 6.898648 | 0.011963 | 0.147422 | unnamed protein product |
| 452 | c69198_g1 | 0 | 1 | 15 | 25 | 13 | 0 | 0.238 | 3.577 | 5.003 | 2.054 | 4.693982 | 0.023958 | 0.237507 | unnamed protein product |
| 453 | c44489_g3 | 1 | 1 | 12 | 46 | 278 | 0.028 | 0.031 | 0.326 | 1.037 | 5.029 | 6.293747 | 0.000635 | 0.014973 | unnamed protein product |
| 454 | c43809_g4 | 1 | 0 | 70 | 153 | 28 | 0.1 | 0 | 8.205 | 15.377 | 2.248 | 6.978463 | 0.000175 | 0.005052 | UPF0326 protein At4g17486 |
| 455 | c30354_g2 | 1 | 0 | 20 | 32 | 31 | 0.157 | 0 | 3.356 | 4.477 | 3.436 | 5.317428 | 0.007039 | 0.100914 | UPF0326 protein At4g17486 |
| 456 | c45654_g8 | 0 | 0 | 245 | 384 | 87 | 0 | 0 | 22.207 | 30.268 | 5.498 | 10.9071 | 2.50E-08 | 2.06E-06 | UPF0497 membrane protein 14 |
| 457 | c41032_g1 | 0 | 1 | 130 | 244 | 161 | 0 | 0.041 | 5.376 | 8.339 | 4.398 | 8.002792 | 2.19E-07 | 1.46E-05 | UPF0503 protein At3g09070 |
| 458 | c32581_g1 | 0 | 0 | 15 | 21 | 36 | 0 | 0 | 2.262 | 2.458 | 4.366 | 7.479786 | 0.00406 | 0.066791 | uridine cytidine kinase I |
| 459 | c25629_g1 | 0 | 0 | 107 | 113 | 74 | 0 | 0 | 11.477 | 10.502 | 5.506 | 9.598669 | 4.63E-06 | 0.00022 | uridine kinase-like protein 5 |
| 460 | c47925_g3 | 0 | 0 | 81 | 314 | 16 | 0 | 0 | 3.871 | 12.417 | 0.501 | 10.09224 | 2.94E-05 | 0.001107 | vacuolar amino acid transporter |
| 461 | c39434_g1 | 1 | 0 | 288 | 306 | 205 | 0.242 | 0 | 51.209 | 48.598 | 31.452 | 8.630314 | 2.70E-09 | 2.68E-07 | vinorine synthase-like |
| 462 | c39012_g1 | 1 | 1 | 45 | 40 | 75 | 0.085 | 0.072 | 3.45 | 2.498 | 4.027 | 5.380834 | 0.001067 | 0.022995 | wee1-like protein kinase-like |
| 463 | c30985_g1 | 0 | 0 | 82 | 325 | 115 | 0 | 0 | 11.982 | 39.573 | 11.117 | 10.38484 | 3.41E-07 | 2.18E-05 | WRKY transcription factor protein |
| 464 | c39947_g1 | 1 | 1 | 49 | 227 | 56 | 0.028 | 0.031 | 1.725 | 6.607 | 1.302 | 6.471612 | 8.44E-05 | 0.002721 | xanthine/uracil permease family |
| 465 | c39981_g1 | 1 | 0 | 673 | 509 | 542 | 0.057 | 0 | 38.702 | 23.701 | 20.844 | 9.732144 | 1.42E-13 | 3.00E-11 | ZF-HD homeobox protein |
| 466 | c36833_g1 | 0 | 0 | 69 | 157 | 57 | 0 | 0 | 9.121 | 16.463 | 4.892 | 9.527442 | 1.17E-05 | 0.000497 | ZF-HD homeobox protein |
| 467 | c32496_g1 | 1 | 0 | 34 | 16 | 49 | 0.114 | 0 | 4.45 | 1.748 | 4.245 | 5.566051 | 0.004837 | 0.07629 | zinc finger family protein |
| 468 | c38352_g2 | 0 | 0 | 54 | 101 | 13 | 0 | 0 | 6.017 | 9.361 | 0.962 | 8.822563 | 0.000423 | 0.010738 | zinc finger protein 7-like |
| 469 | c34349_g1 | 0 | 0 | 25 | 32 | 22 | 0 | 0 | 1.809 | 2.179 | 1.706 | 7.68988 | 0.002591 | 0.046817 | zinc finger protein ZAT4 |
