# Supplementary material for: Differential Gene Expression between Leaf and Rhizome in Atractylodes lancea: A Comparative Transcriptome Analysis
Source: Front Plant Sci. 2016 Mar 30;7:348. doi: 10.3389/fpls.2016.00348 (PMC4811964; doi:10.3389/fpls.2016.00348)
Supplement: Supplementary file 7 [file Table7.docx]

**Supplementary Table 7** DEGs annotated with KEGG metabolic pathways.

| NO. | Pathway | Pathway ID | DEGs with  pathway annotation | All genes with  pathway annotation | P-Value | Corrected P-Value |
| --- | --- | --- | --- | --- | --- | --- |
| 1 | Glycolysis / Gluconeogenesis | ko00010 | 9 | 257 | 0.538016 | 0.855866 |
| 2 | Pentose phosphate pathway | ko00030 | 4 | 98 | 0.448748 | 0.817543 |
| 3 | Pentose and glucuronate interconversions | ko00040 | 9 | 100 | 0.011154 | 0.284438 |
| 4 | Fructose and mannose metabolism | ko00051 | 3 | 89 | 0.600938 | 0.877955 |
| 5 | Galactose metabolism | ko00052 | 12 | 94 | 0.000229 | 0.011704 |
| 6 | Ascorbate and aldarate metabolism | ko00053 | 1 | 71 | 0.914824 | 0.993519 |
| 7 | Fatty acid elongation | ko00062 | 3 | 38 | 0.158084 | 0.691715 |
| 8 | Fatty acid degradation | ko00071 | 2 | 95 | 0.841484 | 0.967604 |
| 9 | Cutin, suberine and wax biosynthesis | ko00073 | 1 | 20 | 0.512017 | 0.855866 |
| 10 | Steroid biosynthesis | ko00100 | 5 | 39 | 0.015492 | 0.316046 |
| 11 | Purine metabolism | ko00230 | 6 | 199 | 0.689493 | 0.936335 |
| 12 | Pyrimidine metabolism | ko00240 | 6 | 165 | 0.514463 | 0.855866 |
| 13 | Alanine, aspartate and glutamate metabolism | ko00250 | 5 | 89 | 0.208619 | 0.691715 |
| 14 | Glycine, serine and threonine metabolism | ko00260 | 3 | 78 | 0.514432 | 0.855866 |
| 15 | Cysteine and methionine metabolism | ko00270 | 4 | 108 | 0.521409 | 0.855866 |
| 16 | Valine, leucine and isoleucine degradation | ko00280 | 2 | 79 | 0.760673 | 0.967604 |
| 17 | Valine, leucine and isoleucine biosynthesis | ko00290 | 2 | 21 | 0.179738 | 0.691715 |
| 18 | Arginine and proline metabolism | ko00330 | 3 | 121 | 0.790648 | 0.967604 |
| 19 | Histidine metabolism | ko00340 | 1 | 36 | 0.717655 | 0.956632 |
| 20 | Phenylalanine metabolism | ko00360 | 6 | 108 | 0.184956 | 0.691715 |
| 21 | Phenylalanine, tyrosine and tryptophan biosynthesis | ko00400 | 1 | 69 | 0.908781 | 0.993519 |
| 22 | beta-Alanine metabolism | ko00410 | 1 | 61 | 0.880019 | 0.981004 |
| 23 | Taurine and hypotaurine metabolism | ko00430 | 1 | 16 | 0.440525 | 0.816973 |
| 24 | Cyanoamino acid metabolism | ko00460 | 3 | 47 | 0.235538 | 0.691715 |
| 25 | Glutathione metabolism | ko00480 | 2 | 108 | 0.887986 | 0.984506 |
| 26 | Starch and sucrose metabolism | ko00500 | 18 | 256 | 0.005383 | 0.183014 |
| 27 | Other glycan degradation | ko00511 | 2 | 35 | 0.354092 | 0.768455 |
| 28 | Amino sugar and nucleotide sugar metabolism | ko00520 | 11 | 173 | 0.046609 | 0.691715 |
| 29 | Streptomycin biosynthesis | ko00521 | 1 | 26 | 0.602518 | 0.877955 |
| 30 | Butirosin and neomycin biosynthesis | ko00524 | 1 | 15 | 0.421074 | 0.800793 |
| 31 | Glycerolipid metabolism | ko00561 | 2 | 94 | 0.837262 | 0.967604 |
| 32 | Inositol phosphate metabolism | ko00562 | 5 | 93 | 0.23303 | 0.691715 |
| 33 | Glycerophospholipid metabolism | ko00564 | 6 | 147 | 0.408444 | 0.800793 |
| 34 | Ether lipid metabolism | ko00565 | 3 | 63 | 0.382559 | 0.780421 |
| 35 | Arachidonic acid metabolism | ko00590 | 1 | 27 | 0.61588 | 0.884785 |
| 36 | Linoleic acid metabolism | ko00591 | 2 | 24 | 0.216732 | 0.691715 |
| 37 | alpha-Linolenic acid metabolism | ko00592 | 2 | 51 | 0.535274 | 0.855866 |
| 38 | Glycosphingolipid biosynthesis - globo series | ko00603 | 1 | 13 | 0.380123 | 0.780421 |
| 39 | Pyruvate metabolism | ko00620 | 4 | 175 | 0.855321 | 0.967604 |
| 40 | Polycyclic aromatic hydrocarbon degradation | ko00624 | 1 | 7 | 0.239087 | 0.691715 |
| 41 | Aminobenzoate degradation | ko00627 | 1 | 9 | 0.289341 | 0.700802 |
| 42 | Butanoate metabolism | ko00650 | 2 | 33 | 0.329435 | 0.738513 |
| 43 | C5-Branched dibasic acid metabolism | ko00660 | 1 | 6 | 0.212645 | 0.691715 |
| 44 | One carbon pool by folate | ko00670 | 2 | 30 | 0.292001 | 0.700802 |
| 45 | Methane metabolism | ko00680 | 4 | 123 | 0.620908 | 0.88577 |
| 46 | Carbon fixation in photosynthetic organisms | ko00710 | 2 | 162 | 0.97563 | 0.999991 |
| 47 | Carbon fixation pathways in prokaryotes | ko00720 | 1 | 56 | 0.857609 | 0.967604 |
| 48 | Thiamine metabolism | ko00730 | 1 | 13 | 0.380123 | 0.780421 |
| 49 | Vitamin B6 metabolism | ko00750 | 2 | 16 | 0.120953 | 0.691715 |
| 50 | Pantothenate and CoA biosynthesis | ko00770 | 2 | 33 | 0.329435 | 0.738513 |
| 51 | Folate biosynthesis | ko00790 | 1 | 24 | 0.574386 | 0.857834 |
| 52 | Porphyrin and chlorophyll metabolism | ko00860 | 1 | 73 | 0.920467 | 0.993519 |
| 53 | Terpenoid backbone biosynthesis | ko00900 | 10 | 75 | 0.000552 | 0.022506 |
| 54 | Limonene and pinene degradation | ko00903 | 1 | 27 | 0.61588 | 0.884785 |
| 55 | Diterpenoid biosynthesis | ko00904 | 2 | 18 | 0.143889 | 0.691715 |
| 56 | Brassinosteroid biosynthesis | ko00905 | 1 | 11 | 0.33628 | 0.745664 |
| 57 | Zeatin biosynthesis | ko00908 | 1 | 22 | 0.544266 | 0.855866 |
| 58 | Sesquiterpenoid and triterpenoid biosynthesis | ko00909 | 2 | 23 | 0.20431 | 0.691715 |
| 59 | Nitrogen metabolism | ko00910 | 4 | 68 | 0.222517 | 0.691715 |
| 60 | Sulfur metabolism | ko00920 | 1 | 34 | 0.697662 | 0.936335 |
| 61 | Phenylpropanoid biosynthesis | ko00940 | 9 | 132 | 0.048434 | 0.691715 |
| 62 | Flavonoid biosynthesis | ko00941 | 3 | 22 | 0.050086 | 0.691715 |
| 63 | Stilbenoid, diarylheptanoid and gingerol biosynthesis | ko00945 | 2 | 16 | 0.120953 | 0.691715 |
| 64 | Aminoacyl-tRNA biosynthesis | ko00970 | 1 | 90 | 0.955598 | 0.999991 |
| 65 | Drug metabolism - other enzymes | ko00983 | 2 | 27 | 0.254307 | 0.691715 |
| 66 | Biosynthesis of ansamycins | ko01051 | 1 | 10 | 0.31321 | 0.734425 |
| 67 | Carbon metabolism | ko01200 | 12 | 433 | 0.818622 | 0.967604 |
| 68 | 2-Oxocarboxylic acid metabolism | ko01210 | 3 | 72 | 0.463259 | 0.836074 |
| 69 | Biosynthesis of amino acids | ko01230 | 15 | 373 | 0.324791 | 0.738513 |
| 70 | ABC transporters | ko02010 | 1 | 43 | 0.777792 | 0.967604 |
| 71 | Ribosome | ko03010 | 2 | 414 | 0.999991 | 0.999991 |
| 72 | RNA transport | ko03013 | 1 | 255 | 0.999849 | 0.999991 |
| 73 | RNA degradation | ko03018 | 2 | 211 | 0.994341 | 0.999991 |
| 74 | RNA polymerase | ko03020 | 1 | 49 | 0.819045 | 0.967604 |
| 75 | DNA replication | ko03030 | 4 | 95 | 0.426243 | 0.800793 |
| 76 | Spliceosome | ko03040 | 4 | 300 | 0.992216 | 0.999991 |
| 77 | PPAR signaling pathway | ko03320 | 2 | 67 | 0.678556 | 0.935306 |
| 78 | Base excision repair | ko03410 | 1 | 56 | 0.857609 | 0.967604 |
| 79 | Nucleotide excision repair | ko03420 | 1 | 100 | 0.968494 | 0.999991 |
| 80 | Mismatch repair | ko03430 | 2 | 69 | 0.69368 | 0.936335 |
| 81 | Homologous recombination | ko03440 | 2 | 76 | 0.742032 | 0.967604 |
| 82 | Fanconi anemia pathway | ko03460 | 2 | 92 | 0.828513 | 0.967604 |
| 83 | MAPK signaling pathway | ko04010 | 6 | 105 | 0.17003 | 0.691715 |
| 84 | ErbB signaling pathway | ko04012 | 2 | 35 | 0.354092 | 0.768455 |
| 85 | MAPK signaling pathway - fly | ko04013 | 2 | 17 | 0.132307 | 0.691715 |
| 86 | Chemokine signaling pathway | ko04062 | 3 | 41 | 0.183021 | 0.691715 |
| 87 | NF-kappa B signaling pathway | ko04064 | 2 | 31 | 0.304524 | 0.72236 |
| 88 | HIF-1 signaling pathway | ko04066 | 4 | 116 | 0.576094 | 0.857834 |
| 89 | Phosphatidylinositol signaling system | ko04070 | 3 | 85 | 0.570671 | 0.857834 |
| 90 | Plant hormone signal transduction | ko04075 | 29 | 269 | 3.17E-07 | 6.46E-05 |
| 91 | Cell cycle | ko04110 | 10 | 199 | 0.166653 | 0.691715 |
| 92 | Cell cycle - yeast | ko04111 | 4 | 160 | 0.804903 | 0.967604 |
| 93 | Meiosis - yeast | ko04113 | 3 | 125 | 0.808095 | 0.967604 |
| 94 | Oocyte meiosis | ko04114 | 5 | 135 | 0.508049 | 0.855866 |
| 95 | p53 signaling pathway | ko04115 | 4 | 71 | 0.244248 | 0.691715 |
| 96 | Ubiquitin mediated proteolysis | ko04120 | 1 | 213 | 0.999355 | 0.999991 |
| 97 | SNARE interactions in vesicular transport | ko04130 | 1 | 53 | 0.842204 | 0.967604 |
| 98 | Protein processing in endoplasmic reticulum | ko04141 | 15 | 352 | 0.253515 | 0.691715 |
| 99 | Lysosome | ko04142 | 1 | 102 | 0.970584 | 0.999991 |
| 100 | Endocytosis | ko04144 | 4 | 228 | 0.954631 | 0.999991 |
| 101 | Phagosome | ko04145 | 10 | 123 | 0.014306 | 0.316046 |
| 102 | Peroxisome | ko04146 | 2 | 120 | 0.91936 | 0.993519 |
| 103 | mTOR signaling pathway | ko04150 | 2 | 47 | 0.493225 | 0.855866 |
| 104 | PI3K-Akt signaling pathway | ko04151 | 8 | 161 | 0.208758 | 0.691715 |
| 105 | Apoptosis | ko04210 | 2 | 30 | 0.292001 | 0.700802 |
| 106 | Vascular smooth muscle contraction | ko04270 | 3 | 40 | 0.174593 | 0.691715 |
| 107 | Wnt signaling pathway | ko04310 | 2 | 78 | 0.754592 | 0.967604 |
| 108 | Dorso-ventral axis formation | ko04320 | 2 | 17 | 0.132307 | 0.691715 |
| 109 | TGF-beta signaling pathway | ko04350 | 3 | 68 | 0.427875 | 0.800793 |
| 110 | Axon guidance | ko04360 | 3 | 48 | 0.244546 | 0.691715 |
| 111 | VEGF signaling pathway | ko04370 | 3 | 41 | 0.183021 | 0.691715 |
| 112 | Osteoclast differentiation | ko04380 | 3 | 37 | 0.150021 | 0.691715 |
| 113 | Hippo signaling pathway | ko04390 | 1 | 76 | 0.928238 | 0.996635 |
| 114 | Hippo signaling pathway - fly | ko04391 | 2 | 52 | 0.545405 | 0.855866 |
| 115 | Focal adhesion | ko04510 | 3 | 67 | 0.418897 | 0.800793 |
| 116 | Adherens junction | ko04520 | 3 | 49 | 0.253607 | 0.691715 |
| 117 | Tight junction | ko04530 | 1 | 59 | 0.871512 | 0.976859 |
| 118 | Gap junction | ko04540 | 10 | 43 | 9.00E-06 | 0.000919 |
| 119 | Antigen processing and presentation | ko04612 | 7 | 124 | 0.152038 | 0.691715 |
| 120 | Toll-like receptor signaling pathway | ko04620 | 3 | 47 | 0.235538 | 0.691715 |
| 121 | NOD-like receptor signaling pathway | ko04621 | 5 | 52 | 0.042032 | 0.691715 |
| 122 | Plant-pathogen interaction | ko04626 | 4 | 229 | 0.955671 | 0.999991 |
| 123 | Natural killer cell mediated cytotoxicity | ko04650 | 3 | 33 | 0.119225 | 0.691715 |
| 124 | T cell receptor signaling pathway | ko04660 | 2 | 38 | 0.390442 | 0.780885 |
| 125 | B cell receptor signaling pathway | ko04662 | 3 | 43 | 0.200187 | 0.691715 |
| 126 | Fc epsilon RI signaling pathway | ko04664 | 3 | 32 | 0.111932 | 0.691715 |
| 127 | Fc gamma R-mediated phagocytosis | ko04666 | 4 | 101 | 0.470967 | 0.836074 |
| 128 | TNF signaling pathway | ko04668 | 2 | 25 | 0.229217 | 0.691715 |
| 129 | Leukocyte transendothelial migration | ko04670 | 1 | 21 | 0.528417 | 0.855866 |
| 130 | Circadian rhythm | ko04710 | 1 | 20 | 0.512017 | 0.855866 |
| 131 | Circadian rhythm - plant | ko04712 | 2 | 55 | 0.574863 | 0.857834 |
| 132 | Circadian entrainment | ko04713 | 2 | 17 | 0.132307 | 0.691715 |
| 133 | Long-term potentiation | ko04720 | 2 | 55 | 0.574863 | 0.857834 |
| 134 | Synaptic vesicle cycle | ko04721 | 2 | 91 | 0.823982 | 0.967604 |
| 135 | Neurotrophin signaling pathway | ko04722 | 5 | 80 | 0.157223 | 0.691715 |
| 136 | Retrograde endocannabinoid signaling | ko04723 | 3 | 24 | 0.060667 | 0.691715 |
| 137 | Glutamatergic synapse | ko04724 | 2 | 73 | 0.722164 | 0.956632 |
| 138 | Cholinergic synapse | ko04725 | 2 | 20 | 0.167631 | 0.691715 |
| 139 | Serotonergic synapse | ko04726 | 2 | 21 | 0.179738 | 0.691715 |
| 140 | GABAergic synapse | ko04727 | 2 | 37 | 0.378421 | 0.780421 |
| 141 | Long-term depression | ko04730 | 2 | 40 | 0.414162 | 0.800793 |
| 142 | Regulation of actin cytoskeleton | ko04810 | 5 | 99 | 0.271015 | 0.700802 |
| 143 | Insulin signaling pathway | ko04910 | 3 | 128 | 0.820362 | 0.967604 |
| 144 | GnRH signaling pathway | ko04912 | 2 | 63 | 0.646486 | 0.915856 |
| 145 | Progesterone-mediated oocyte maturation | ko04914 | 7 | 98 | 0.063432 | 0.691715 |
| 146 | Estrogen signaling pathway | ko04915 | 7 | 121 | 0.139745 | 0.691715 |
| 147 | Melanogenesis | ko04916 | 2 | 43 | 0.448847 | 0.817543 |
| 148 | Prolactin signaling pathway | ko04917 | 2 | 32 | 0.317006 | 0.734878 |
| 149 | Adipocytokine signaling pathway | ko04920 | 2 | 38 | 0.390442 | 0.780885 |
| 150 | Type II diabetes mellitus | ko04930 | 6 | 50 | 0.010949 | 0.284438 |
| 151 | Type I diabetes mellitus | ko04940 | 1 | 21 | 0.528417 | 0.855866 |
| 152 | Aldosterone-regulated sodium reabsorption | ko04960 | 2 | 14 | 0.099056 | 0.691715 |
| 153 | Endocrine and other factor-regulated calcium reabsorption | ko04961 | 1 | 42 | 0.770056 | 0.967604 |
| 154 | Vasopressin-regulated water reabsorption | ko04962 | 3 | 32 | 0.111932 | 0.691715 |
| 155 | Pancreatic secretion | ko04972 | 3 | 32 | 0.111932 | 0.691715 |
| 156 | Carbohydrate digestion and absorption | ko04973 | 1 | 26 | 0.602518 | 0.877955 |
| 157 | Fat digestion and absorption | ko04975 | 1 | 7 | 0.239087 | 0.691715 |
| 158 | Bile secretion | ko04976 | 4 | 50 | 0.107207 | 0.691715 |
| 159 | Mineral absorption | ko04978 | 1 | 18 | 0.477491 | 0.839725 |
