# Supplementary figures and images for: Differential Gene Expression between Leaf and Rhizome in Atractylodes lancea: A Comparative Transcriptome Analysis
Source: Front Plant Sci. 2016 Mar 30;7:348. doi: 10.3389/fpls.2016.00348 (PMC4811964; doi:10.3389/fpls.2016.00348)

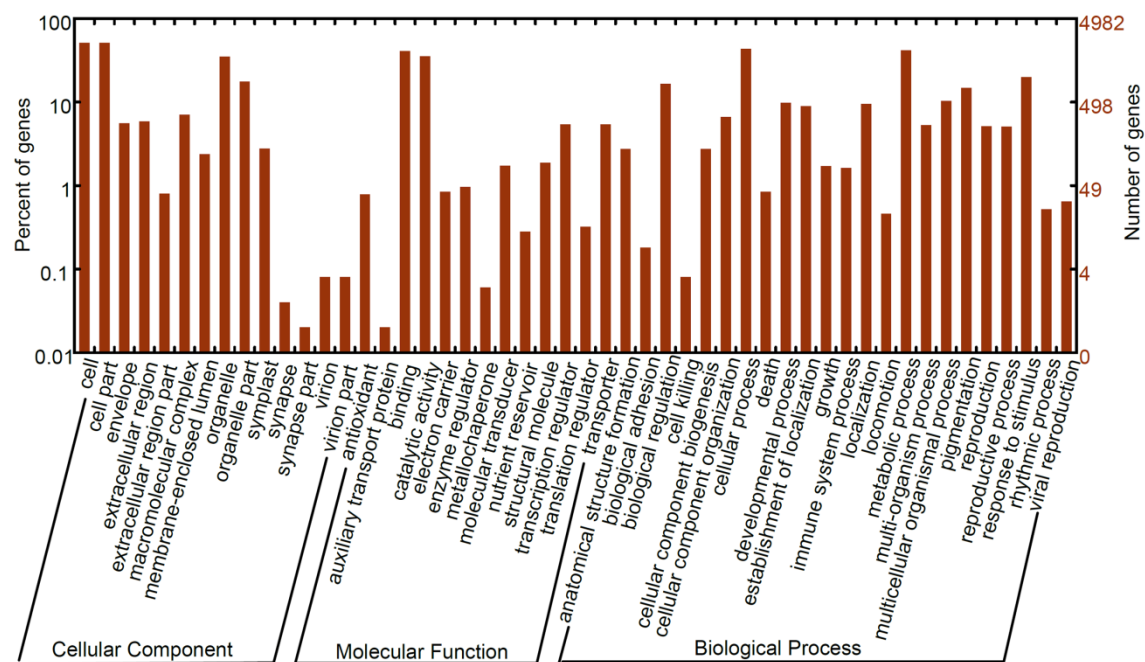

**Supplementary Figure S1** GO classification of DEGs.

Supplement: Supplementary file 8 [file Image1.pdf]
