# Supplementary material for: Differential Gene Expression between Leaf and Rhizome in Atractylodes lancea: A Comparative Transcriptome Analysis
Source: Front Plant Sci. 2016 Mar 30;7:348. doi: 10.3389/fpls.2016.00348 (PMC4811964; doi:10.3389/fpls.2016.00348)
Supplement: Supplementary file 9 [file Image2.pdf]

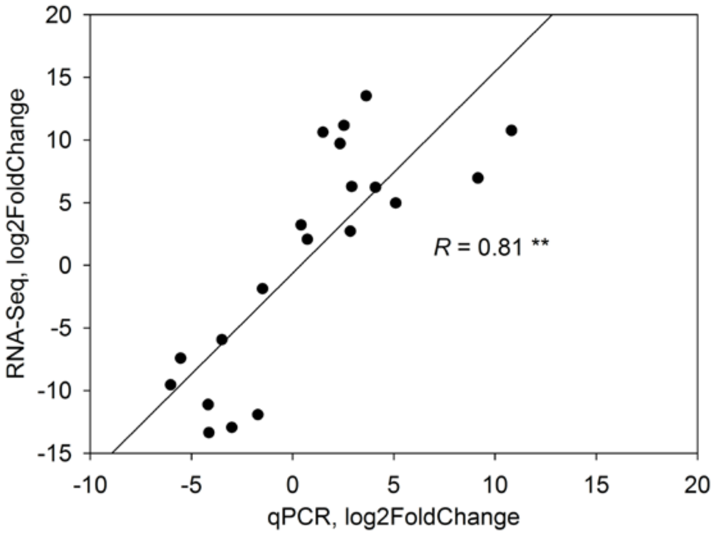

Supplementary Figure S2 Scatter plot of the log2FoldChange values for 20 selected genes as estimated by RNA-seq and qPCR analyses. The correlation coefficient (R) is shown (**$p < 0.01$).
